# Synthesis of a peripherally conjugated 5-6-7 nanographene†
†Electronic supplementary information (ESI) available: Experimental procedures, spectra of new compounds, and details of computational studies. See DOI: 10.1039/c5sc03280f


**DOI:** 10.1039/c5sc03280f

**Published:** 2015-09-29

**Authors:** Marika Żyła, Elżbieta Gońka, Piotr J. Chmielewski, Joanna Cybińska, Marcin Stępień

**Affiliations:** a Wydział Chemii , Uniwersytet Wrocławski , ul. F. Joliot-Curie 14 , 50-383 Wrocław , Poland . Email: marcin.stepien@chem.uni.wroc.pl; b Department of Nanotechnology , Wrocław Research Centre EIT+ , ul. Stabłowicka 147 , 54-066 Wrocław , Poland

## Abstract

A heteroaromatic nanographene containing a unique assembly of five-, six- and seven-membered rings is synthesized using oxidative coupling of an indole-containing precursor. Near-infrared absorption and emission properties of the nanographene core are enhanced by peripheral expansion and ring fusion at all oxidation levels. The dicationic state shows distinct aromaticity originating from a peripheral π-conjugated circuit. A partially coupled intermediate, trapped in the synthesis of the 5-6-7 nanographene, is explored as a reference system, showing an unexpected reduction of the optical band gap due to intramolecular charge transfer.

## Abstract 2

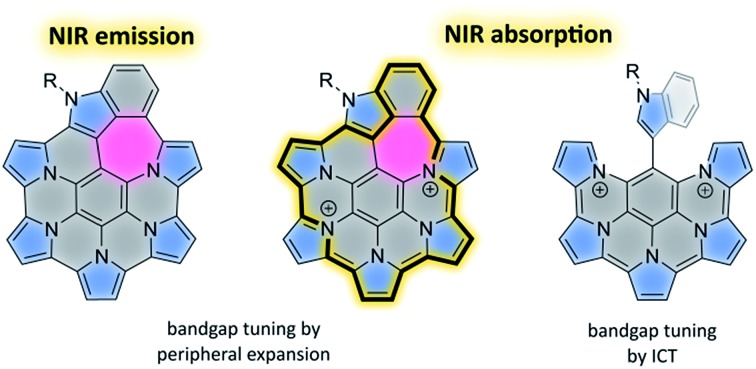
Expansion of heteroaromatic nanographenes *via* 5-6-7 fusion enhances their NIR absorption and emission properties.

## Introduction

Doping large aromatic structures with heteroatoms is one of the key exploration areas in current nanographene research.^1^,^2^ While structurally precise doping of graphene sheets is yet to be achieved, bottom-up approaches developed for the synthesis of extended polycyclic aromatic hydrocarbons (PAHs) have been investigated as potential routes to doped nanographenes and nanoribbons. Heteroaromatic five-membered (N,^3^–^12^ P,^13^ S^14^–^23^) and six-membered rings (B,^18^,^24^–^26^ N,^24^–^38^ O,^29^,^30^ S^29^,^30^) have recently been incorporated into large PAH-like structures, yielding systems with distinct electronic and optical properties. In nanoribbons, atomically precise introduction of nitrogens has been found to produce band shifts corresponding to n-type doping, and enabled charge transport tuning and the fabrication of heterojunctions and heterostructures.^39^–^41^ At present, knowledge of the site-specific effects of heteroatom doping is limited, and such influences are best explored by preparing structurally new nanographene molecules. However, established synthetic routes to PAHs are often incompatible with heterocyclic precursors, and need to be refined or replaced in order to encompass new structural designs.

**Scheme 1:**
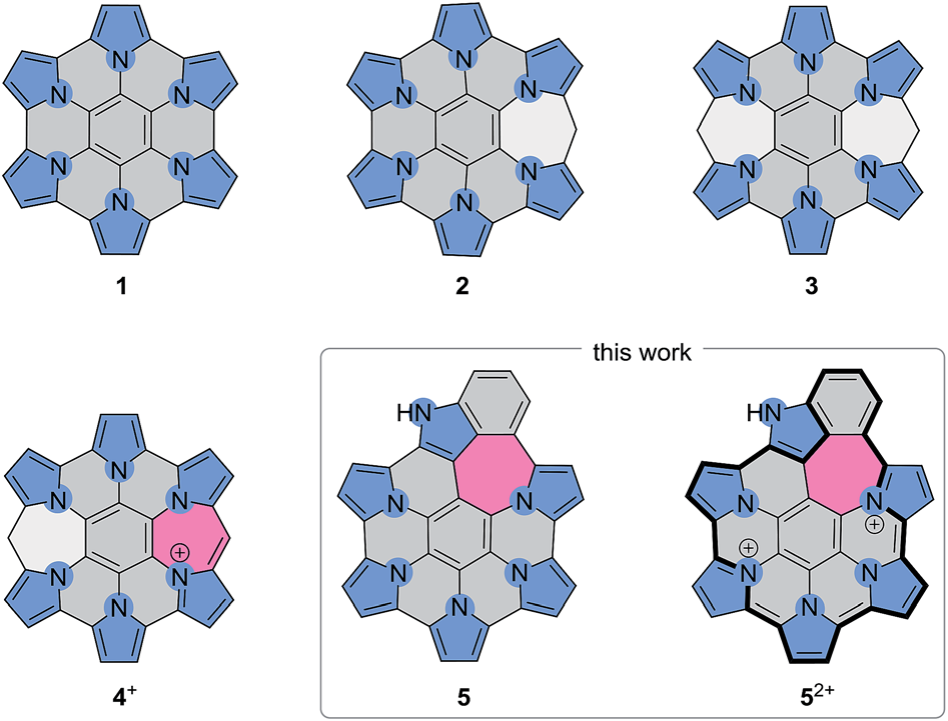
Pyrrole-fused azacoronene systems.

An elegant route to heteroatom-doped coronene derivatives involves the oxidative coupling of star-shaped aromatic precursors containing heterocyclic groups.^17^,^27^,^34^,^36^ Such reactions have been modeled after the classical synthesis of hexa-*peri*-hexabenzocoronenes (*p*-HBCs).^42^ A remarkable example of this approach is provided by the FeCl_3_-mediated coupling of hexapyrrolylbenzenes,^43^ which was found to produce hexapyrrolohexaazabenzocoronenes (HPHACs, **1**, Scheme 1).^3^,^44^ HPHACs and the related HPHAC–HBC hybrids^4^ are fluorescent in their neutral oxidation states and can be converted into strongly NIR-absorbing cations by reversible multistep oxidation. The work on hybrid systems showed, however, that the effectiveness of coupling is dependent on the precursor design, notably on the choice of subunits and peripheral substitution.^4^

We have recently reported HPHAC analogues containing non-conjugated bridges on the periphery (**2–3**).^11^ Up to four electrons could be removed reversibly from these systems, leading to cationic species with extended NIR absorptions. In these oxidations, the benzylidene bridges were found to be remarkably resistant to dehydrogenation, and complete peripheral π conjugation could not be achieved. Stereospecific oxidation of a single bridge could however be induced in **3**, to provide the monocation **4^+^**, the first example of a heteroaromatic nanographene structure with a conjugated 7-membered ring. The inclusion of 7-membered rings in PAH and related structures is known to affect the curvature and electronic conjugation of fused frameworks, however, the construction of such systems is still a non-routine task.^45^–^49^ In conjunction with heteroatom doping, non-benzenoid fusion is an attractive method of increasing structural diversity in nanographenes. The rich chemistry of peripherally expanded azacoronenes **2–3** inspired us to seek synthetic designs that would produce systems with fully conjugated and non-trivial ring fusion patterns. Here we show that heteroaromatic nanographene structures containing a combination of 5-, 6-, and 7-membered rings (“5-6-7 nanographenes”) can be obtained by the direct oxidative coupling of star-shaped precursors containing pyrrole and indole arms. The electronic structure of the new nanographene is compared with a partially coupled reference system. We demonstrate for the first time how the absorption and emission properties of such nanographenes can be tailored by peripheral expansion and ring fusion.

## Results and discussion

**Scheme 2:**
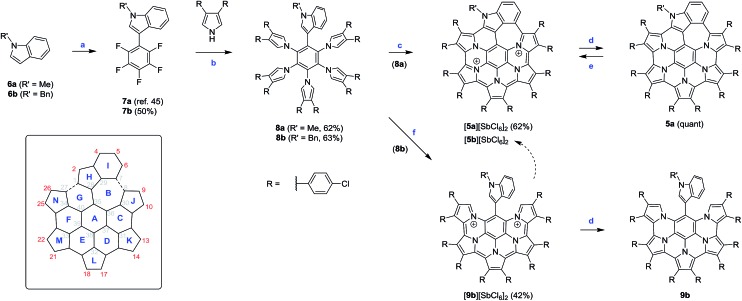
Synthetic work and the labeling scheme for **5** and **9**. Reagents and conditions: (a) Ag_2_CO_3_ (1.5 equiv.), Pd(OAc)_2_ (cat.), AcOH (1 equiv.), pentafluorobenzene (3 equiv.), DMF/DMSO, 120 °C. (b) 1. NaH (5 equiv.), diarylpyrrole (5 equiv.), DMF, ice bath, 2. **7a** or **7b**, 50 °C, overnight. (c) BAHA (12 equiv.), Et_2_O/THF, RT. (d) Zn amalgam or Zn powder, DCM or CDCl_3_, quant. (e) BAHA, (NO)[SbF_6_], or DDQ; DCM or MeCN, quant. (f) BAHA (10 equiv.), Et_2_O/THF, RT, 42%.

The requisite indole-containing precursors **8a**–**b** were prepared in two steps from *N*-substituted indoles **6a**–**b** (Scheme 2). **6a**–**b** were coupled with pentafluorobenzene, using Pd/Ag-mediated direct double C–H activation,^50^ to yield C_6_F_5_-substituted derivatives **7a**–**b**. The latter intermediates were subjected to quintuple nucleophilic substitution with β-substituted pyrroles,^9^,^51^,^52^ to yield the star-shaped **8a**–**b** in up to 63% yield. In contrast to our previous work, which had used *p*-butoxyphenyl substituents on the periphery of nanographenes **2–3**,^11^*p*-chlorophenyl groups were introduced to the structures of **8a**–**b**. The electron-withdrawing character of the latter substituents and their limited solubilizing effect in nonpolar solvents provided better stability and easier isolation, respectively, of the subsequent coupling products.

Oxidative coupling reactions are well suited for the rapid development of complex aromatic molecules; however, their use is often limited by reactivity and selectivity problems.^53^ In dichloromethane solutions, **8a** was not reactive towards DDQ, but it was easily oxidized by ionic reagents such as FeCl_3_,^3^,^4^,^11^ Fe(ClO_4_)_3_, Ag[SbF_6_], or (NO)[SbF_6_]. Disappointingly, all these oxidants produced mixtures of products that could not be separated without decomposition. It was subsequently found that **8a** was cleanly oxidized by tris(4-bromophenyl)ammoniumyl hexachloroantimonate (BAHA),^54^ a one-electron, radical-cation reagent with preparative applications in oligopyrrole chemistry.^55^–^57^ The oxidation, performed with 12 equiv. of BAHA in a diethyl ether/THF mixture, led directly to the dicationic species **5a^2+^**, which was isolated in the form of the dark-brown hexachloroantimonate salt in 62% yield. The isolation was particularly convenient because [**5a**][SbCl_6_]_2_ precipitated from the solution, and after an additional crystallization from dichloromethane/hexane was found to be free of the oxidant byproducts. The reaction with BAHA strongly favors the formation of **5a^2+^** and small amounts of the salt could be isolated even when 2 equiv. of the oxidant were used. [**5a**][SbCl_6_]_2_ was quantitatively converted to the neutral species **5a** by reduction with zinc amalgam.

Interestingly, when the benzyl-substituted derivative **8b** was subjected to the same BAHA-induced oxidation, the outcome of the reaction was different. The green precipitate that formed was found to contain mainly the partly coupled product [**9b**][SbCl_6_]_2_. Under optimized conditions, involving oxidation with 10 equiv. of BAHA in THF/diethyl ether, the above salt was isolated in 42% yield. In analogy to [**5a**][SbCl_6_]_2_, compound [**9b**][SbCl_6_]_2_ was cleanly reduced by zinc amalgam, to yield the corresponding neutral species **9b**. A benzene-containing molecule, similar to **9b**, was previously reported by Takase *et al.*^4^ but the accessibility of higher oxidized states was not disclosed for that system. Dication **9b^2+^** is moderately stable in solution, undergoing gradual conversion to **5b^2+^** in the presence of air.

The extent of oxidative coupling and the charge of the products was determined using ESI mass spectrometry. In the mass spectra recorded for the oxidation products of **8a**–**b**, peaks corresponding to singly (**5a^+^**, **5b^+^**) and doubly charged species (**5a^2+^**, **5b^2+^**) could be identified on the basis of the observed *m*/*z* ratio and isotope patterns. The partially coupled dication **9b^2+^** produced only peaks corresponding to **5b^2+^** and **5b^+^**, indicative of instantaneous dehydrogenation in the ESI source.

**Fig. 1:**
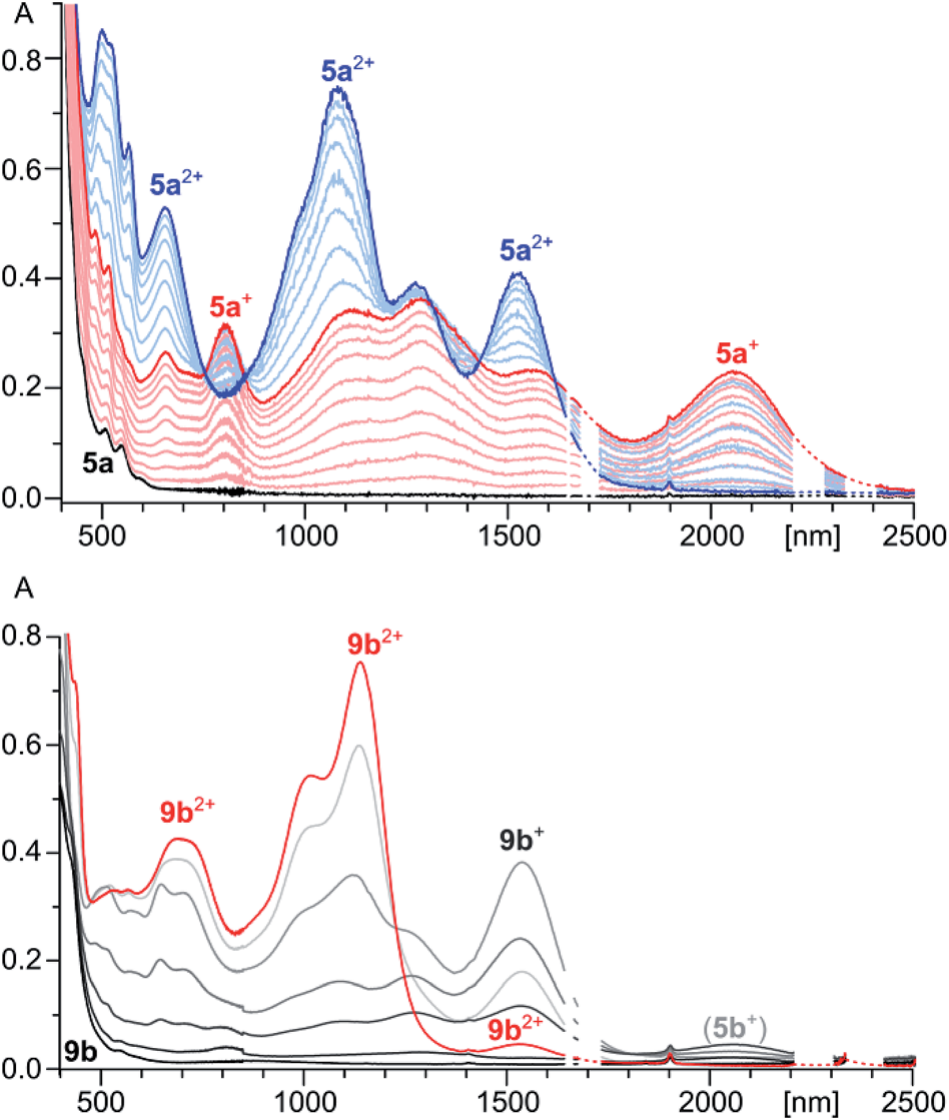
Top: vis-NIR spectra of **5a^+^** (red) and **5a^2+^** (blue) obtained by spectrophotometric titration of **5a** (black) with BAHA (dichloromethane, 293 K). Bottom: vis-NIR spectrum of **9b^2+^** (red trace, SbCl_6_ salt, dichloromethane). Gray traces correspond to stepwise *in situ* reduction of **9b^2+^** with Zn powder. Apart from **9b^+^** and **9b**, small amounts of **5b^+^** are also formed in contact with air.

The spectroscopic features of **5a^2+^** are consistent with extensive π-electron conjugation. In dichloromethane solutions, the dication has an intense brown color, with electronic absorptions covering the entire visible range and reaching beyond 1800 nm in the near infrared (Fig. 1). With the lowest-energy absorption at 1525 nm, the NIR part of the spectrum of **5a^2+^** shows an overall red shift relative to the reported spectrum of an aryl substituted HPHAC dication (**1^2+^**),^3^ correlating with the more extended π-conjugation in the indole-containing nanographene. In the spectrum of **9b^2+^**, major NIR absorptions are located in the 1000–1150 nm range. However, a weak maximum at 1530 nm, observed consistently for various samples of **9b^2+^**, indicated that the optical HOMO–LUMO gaps of the two dications are almost identical (0.81 eV). This unexpected result was rationalized using TD-DFT calculations (*vide infra*).

**Fig. 2:**
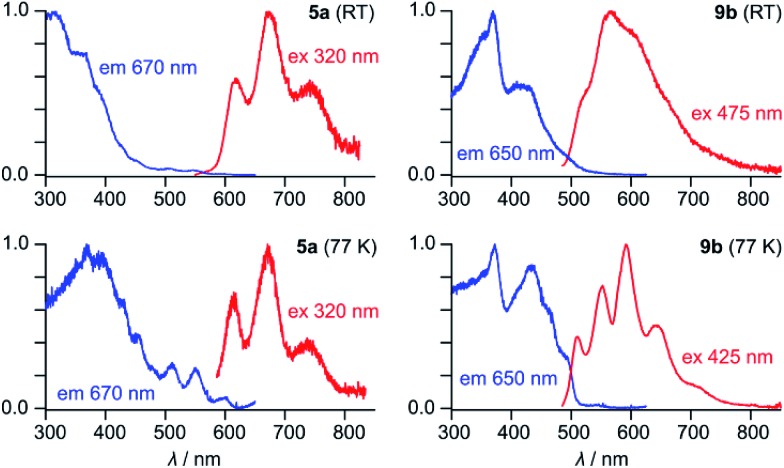
Fluorescence emission and excitation spectra of **5a** and **9b** recorded in dichloromethane.

**5a** has an orange color in solution and displays noticeable orange fluorescence. With several weak bands in the 500–600 nm range (*ε* = 900–2700 M^–1^ cm^–1^), the absorption spectrum of **5a** resembles the optical spectra of other neutral HPHAC systems, although a red shift of the lowest energy transition is clearly observable.^3^,^4^,^11^ The fluorescence emission spectrum of **5a** displays a vibronically resolved pattern with major maxima at 620, 675 and 740 nm (Δ*E* ∼ 1300 cm^–1^, Fig. 2). The fluorescence excitation spectrum of **5a** accurately reproduces the absorption profile above 400 nm, including the three weak bands with *λ* > 500 nm. The emission quantum yield of **5a** (*ca.* 0.5%) is in the range reported for related nanographene systems (0.1–1.8%) and the small radiative rate constants *k*_f_ = *Φ*_F_/*τ*_F_ ≈ 10^6^ s^–1^ are also consistent with the semi-allowed character of the S_0_ → S_1_ transitions.^4^ The moderate Stokes shift in **5a** (*ca.* 680 cm^–1^) indicates a relatively small reorganization of the nanographene chromophore in the excited state. In spite of the small Stokes shift, the fluorescence of **5a** is noticeably red-shifted in comparison with previously reported HPHAC-like systems.^3^,^4^ In DCM solutions, **9b** has a yellow color and shows yellow fluorescence corresponding to a broadened emission (*λ*_max_ ≈ 565 nm), which becomes well-resolved at low temperatures. The *λ*_0–0_ peak (510 nm at 77 K in frozen DCM solution) corresponds to a Stokes shift of *ca.* 900 cm^–1^. Neither **5a** nor **9b** exhibited detectable phosphorescence in frozen dichloromethane at 77 K.

**Fig. 3:**
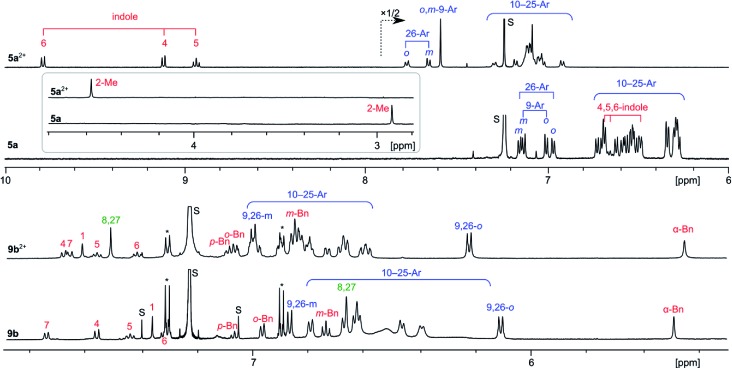
^1^H NMR spectra (CDCl_3_, 600 MHz) recorded for [**5a**][SbCl_6_]_2_, **5a** (300 K), [**9b**][SbCl_6_]_2_, and **9b** (260 K). Signal assignments are based on correlation spectroscopy (see the ESI†).

The ^1^H NMR spectra of **5a** and **5a^2+^** (Fig. 3) each contain a large number of signals, consistent with the low effective molecular symmetry (*C*_s_) of the nanographene. Because of the severe overlap of the aryl signals, only partial spectral assignment could be achieved with the use of ^1^H COSY and ROESY spectra. The differences of the chemical shifts reveal a drastic change in the magnetic properties of **5a** attendant upon oxidation, indicative of a considerable increase of the aromatic character of the core. In the spectrum of neutral **5a**, the indole signals 4-H, 5-H, and 6-H were found at 6.69, 6.65, and 6.49 ppm, respectively, obscured by the numerous *p*-chlorophenyl peaks clustered in the 7.2–6.2 ppm range. With the exception of the 9- and 26-Ar groups adjacent to the indole, the aryl signals of **5a** are shifted slightly upfield relative to their positions in **8a**, an effect caused by steric crowding of the substituents. In **5a^2+^**, the indole signals are shifted by up to 3.3 ppm towards the lower field relative to **5a**, whereas smaller relocations are observed for the Me group (+1.6 ppm) and aryl signals (up to +0.8 ppm). Such a distance dependence of the deshielding effect is consistent with a marked increase of the diatropic ring current in the oxidized nanographene core. This diatropic effect is clearly reproduced by GIAO-DFT calculations of proton shifts, performed for **5a^2+^** and **5a** (Fig. S26–S28†). The molecular structure of **5a^2+^** requires 75 carbon signals (including 51 quaternary sp^2^ centers), which were identified in the ^13^C NMR spectrum, and assigned into chemically distinct groups using correlation spectroscopy and GIAO-DFT calculations (Fig. S9–S10†). In particular, the six non-equivalent carbons of the inner ring A were identified in a markedly upfield region (98.7–109.7 ppm). ^1^H and ^13^C shifts obtained from the spectroscopic analyses correlate very well with the GIAO predictions.

The ^1^H NMR spectra of **9b^2+^** and **9b** reveal the chemical equivalence of 9-Ar and 26-Ar substituents, which is only possible in the case of a non-fused indole unit. In both species, signals corresponding to the intact 3-(1-benzyl)-indolyl group and the unique signal of pyrrole 8,27-α protons were identified using correlation spectroscopy (Fig. 3). These features and the absence of other α-pyrrole signals clearly confirm that the oxidative coupling occurred only between adjacent pyrrole units. In contrast to the **5a**/**5a^2+^** pair, the ^1^H shifts of **9b** and **9b^2+^** are similar, indicating that the oxidation of the fused core does not lead to any significant increase of overall diatropicity.

**Fig. 4:**
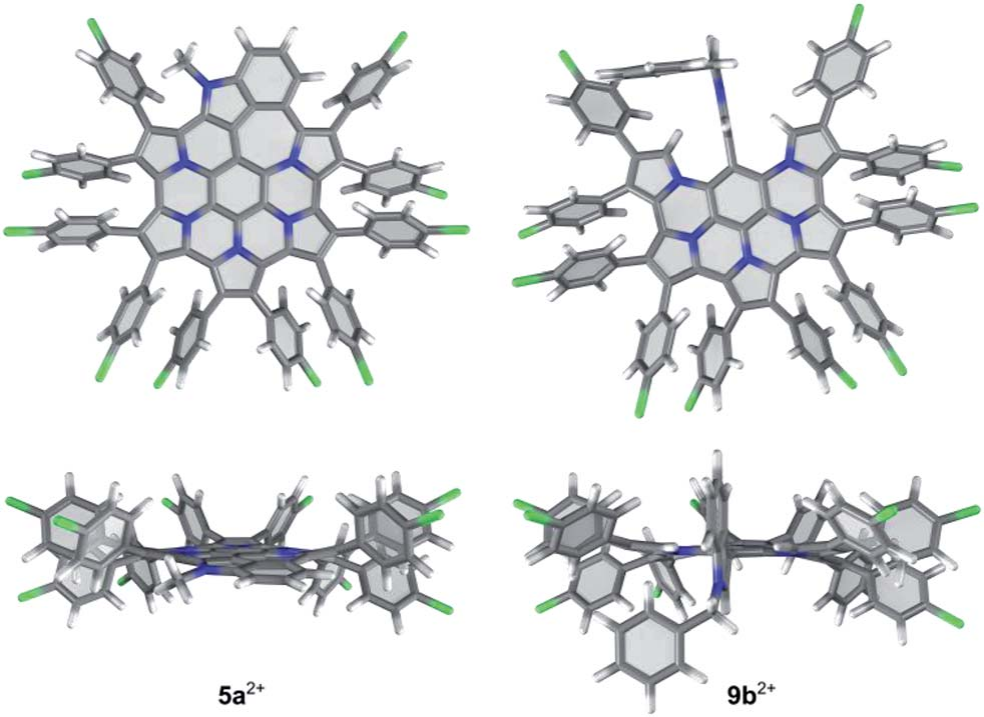
DFT-optimized geometries of **5a^2+^** and **9b^2+^**. The lowest-energy conformer found for **5a^2+^** is shown.

DFT-optimized geometries of the indole-containing nanographene are similar in all oxidation states (**5a**, **5a^+^** and **5a^2+^**). As shown in Fig. 4 for the dication **5a^2+^**, the fused core is relatively planar, with slight out-of-plane distortions along the periphery. In contrast to substituted HPHAC's, in which the rotation axes of aryl groups are coplanar with the core, in **5a^2+^**, the substituents are bent away from the central plane and tilted, to reduce steric interactions. Out of eight distinct conformers differing in the relative arrangement of aryl groups, the majority have relative energies within a range of only 1 kcal mol^–1^. The lowest-energy structure is characterized by an alternating up-and-down arrangement of consecutive pyrrole units. The inter-subunit C–C bonds in **5a^2+^** (those formed in the course of oxidative coupling) are in the range 1.418 to 1.450 Å and are systematically shorter in comparison to the corresponding distances in **5a** (1.436 to 1.470 Å). In contrast to the parent HPHAC system **1**, which was predicted by DFT to adopt a bowl shape,^3^ the DFT-optimized structures of the unsubstituted **5**, **5^+^**, and **5^2+^** are completely planar, indicating that the internal strain of the fused framework is partly relieved by the introduction of the 7-membered ring.

The optimized structure of **9b^2+^** shows that the indole moiety is perpendicular to the fused nanographene unit and is confined between the two adjacent pyrrolic α-hydrogens. In **5b^2+^**, the presence of the benzyl substituent has negligible influence on the conformation of the aromatic core, in comparison with **5a^2+^**.

**5a**, obtained by *in situ* reduction of [**5a**][SbCl_6_]_2_ with zinc amalgam, was subjected to a spectrophotometric titration with BAHA (Fig. 1). This experiment revealed the intermediate formation of a light brown radical cation **5a^+^**, characterized by extended NIR absorption, reaching up to 2400 nm. Subsequent addition of BAHA to the solution led to the recovery of **5a^2+^**, however, no further oxidation could be induced with either BAHA or the more strongly oxidizing (NO)[SbF_6_]. The two observable oxidation events are sufficiently well separated to produce approximate isosbestic points. Alternatively, **5a^+^** could be generated by comproportionation of **5a^2+^** and **5a** combined in 1 : 1 molar ratio in a dichloromethane solution. The radical nature of **5a^+^** was confirmed using ESR spectroscopy, which yielded a signal at *g* = 2.0026 with no resolvable hyperfine structure (Fig. S15†). Trace amounts of the **5a^+^** radical form spontaneously in solutions of both **5a** and **5a^2+^**, leading to a gradual broadening of the ^1^H NMR signals. This effect was suppressed by the addition of a small amount of BAHA or zinc amalgam to the solutions of **5a^2+^** and **5a**, respectively, to enable the recording of well-resolved NMR spectra. In the case of **5a^2+^** the differential broadening of the indole signals (4,5,6-H) caused by the radical admixture was found to correlate well with the calculated spin density distribution (Fig. S24 and S25†).

The chemical oxidation of **8a** with BAHA, followed spectrophotometrically in dilute DCM solutions, revealed that, even with a substoichiometric amount of the oxidant, the reaction leads directly to **5a^2+^**, without the formation of the radical cation **5a^+^**. This observation, which contrasts with the behavior of **5a**, indicates that either (a) **5a^2+^** is the direct product of the coupling or (b) the small amount of **5a**, produced progressively during the reaction, is the first species to consume any excess of the introduced oxidant. The analogous oxidation of **8b** revealed the initial formation of **9b^2+^**, which was gradually replaced with **5b^2+^** as the titration progressed. It seems that, because of the steric hindrance introduced by the benzyl group, the conversion of **9b^2+^** into **5b^2+^** is slowed down sufficiently to enable the isolation of the former species by precipitation of its SbCl_6_ salt. However, when **9b^2+^** is not precipitated from the solution, it is oxidized to **5b^2+^** with observable kinetics.

Voltammetric experiments performed on [**5a**][SbCl_6_]_2_ revealed two electrochemically reversible redox events at –0.17 and –0.02 V (*vs.* Fc/Fc^+^), corresponding respectively to the **5a^0^**/**5a^+^** and **5a^+^**/**5a^2+^** couples (Fig. S16†). The observed potential difference corresponds to a comproportionation constant *K*_c_ of *ca.* 3.5 × 10^2^, sufficiently high to make the radical cation an observable species in solution. At 0.80 and 1.04 V, two additional oxidations were observed, likely associated with the formation of higher cationic species. The large gap between the second and third oxidation (0.82 V) is thought to result from the aromatic stabilization of **5a^2+^**. Interestingly, when precursor **8a** was electrolyzed in tetrabutylammonium perchlorate/dichloromethane at a constant potential of 1.4 V *vs.* Fc/Fc^+^, the formation of **5a^2+^** was observed spectrophotometrically (Fig. S17–S18†). The **5a^2+^** thus formed yielded an electrochemical signature matching that of the chemically generated dication.

TD-DFT calculations performed for the differently oxidized states of **5a** and **9b** yielded results in very good agreement with the experiment (Table 2 and S2–S7†). In particular, an excellent linear correlation was found between the experimental and theoretical HLGs (Fig. S29†). At the PCM(CH_2_Cl_2_)/TD-B3LYP/6-31G(d,p) level of theory, a number of partially allowed transitions (*f* < 0.09) are predicted for **5a** (400–570 nm), engaging excitations between three highest occupied and four lowest unoccupied MO levels. Similarly weak absorptions in the visible region are found in the TD spectrum of **9a**. In contrast, excitations to levels higher than LUMO provide no significant contributions to the calculated vis-NIR transitions of **5a^2+^** and other cationic species. **5a^2+^**, **9b^+^**, and **9b^2+^** are shown to have very similar optical band-gaps with a lower oscillator strength predicted for the latter species, as experimentally observed. Additionally, a significantly smaller HLG is correctly predicted for **5a^+^**.

**Table 1:** Optical properties of **5a**, **9b** and their oxidized states

	Absorption^*a*^		Emission^*a*^			
	*λ*_max_ (*ε*) nm (10^3^ M^–1^ cm^–1^)	HLG^*b*^ eV	*λ*_0–0_ nm	Stokes shift cm^–1^	*Φ*_F_ ^*c*^	*τ*_F_ ^*d*^ ns
**5a**	595 (0.9), 550 (2.4), 520 (2.7), 450^*e*^, 430 (11.8)	2.08	630 (615)	678	0.005	4.1
**5a^+^**	2050 (5.9), 1560 (6.0), 1285 (9.3), 1120 (8.8), 805 (7.9), 658 (6.8), 516 (10.8), 484 (12.5)	0.60				
**5a^2+^**	1525 (10.3), 1270 (10.3), 1080 (18.3), 656 (12.8), 566 (15.3), 520 (20.0), 498 (20.5)	0.81				
**9b**	475^*e*^	2.61	∼520^*e*^ (510)	926^*f*^	0.006	5.9
**9b^+^**	1540, 1275, 1090, 712, 647, 586	0.81				
**9b^2+^**	1530 (1.5), 1140 (24.0), 1020 (17.4), 705 (13.6), 572 (10.6), 439 (22.8)	0.81				

^*a*^In dichloromethane solutions.

^*b*^Optical HOMO–LUMO gap determined from the lowest energy band.

^*c*^Fluorescence quantum yield.

^*d*^Fluorescence decay time.

^*e*^Shoulder.

^*f*^Determined from emission and excitation spectra at 77 K.

**Table 2:** Geometrical and electronic structure data obtained from DFT calculations

Species^*a*^/parameter		Charge		
		*n* = 0	*n* = 1	*n* = 2
**5a*^n^*^+^**				
Distance [Å]	C_α_–C_β_	1.387–1.402	1.393–1.418	1.405–1.435
	C_β_–C_β_	1.426–1.445	1.418–1.428	1.407–1.417
	C_α_–N	1.376–1.416	1.372–1.416	1.368–1.415
	C_α_–C_α_	1.441–1.456	1.432–1.438	1.418–1.423
HLG,^*b*^ ^,^^*c*^ eV (nm)		2.19 (567)	0.71 (1748)	0.93 (1332)
*f* ^*b*^ ^,^ ^*d*^		0.009	0.106	0.156

**5*^n^*^+^**				
NICS(1)^*e*^ [ppm]	A, B	–8.5, 6.2		–19.7, –10.1
	C–G	–0.9 to 5.5		–12.6 to –14.0
	H, I	–9.9, –8.3		–10.2, –21.1
	J–N	–10.2 to –6.8		–14.7 to –11.1
HLG,^*b*^ ^,^^*c*^ eV (nm)		2.20 (564)	0.78 (1590)	1.00 (1242)
*f* ^*b*^ ^,^ ^*d*^		0.007	0.061	0.092

**9b*^n^*^+^**				
HLG,^*b*^ ^,^^*c*^ eV (nm)		2.62 (472)	0.92 (1348)	0.99 (1255)
*f* ^*b*^ ^,^ ^*d*^		0.006	0.282	0.019

**9*^n^*^+^**				
HLG,^*b*^ ^,^^*c*^ eV (nm)		2.63 (471)	1.04 (1194)	1.05 (1180)
*f* ^*b*^ ^,^ ^*d*^		0.004	0.156	0.017

**1*^n^*^+^**				
HLG,^*b*^ ^,^^*c*^ eV (nm)		2.41 (515)	1.05 (1175)	1.35 (918)
*f* ^*b*^ ^,^ ^*d*^		0.000	0.083	0.123

^*a*^PCM(CHCl_3_)/B3LYP/6-31G(d,p) geometries.

^*b*^PCM(DCM)/TD-B3LYP/6-31G(d,p).

^*c*^HOMO–LUMO gap.

^*d*^Oscillator strength.

^*e*^GIAO-B3LYP/6-31G(d,p).

Because of the different substitution patterns found in **5a** and the reported derivatives of **1**,^3^,^4^ the influence of 5-6-7 fusion and peripheral expansion on the optical bandgaps of pyrrole-based nanographenes cannot be precisely separated from substituent effects. The TD-DFT band gap calculated for the unsubstituted **5^2+^** (1.00 eV) is smaller by 0.35 eV than in **1^2+^** (Table 2). This decrease is noteworthy given that it is caused by the addition of only four centers to a 36-atom π-conjugated system. The HLG differences are progressively smaller between the lower oxidation levels of **5** and **1** (0.27 eV for the radical cation, and 0.21 eV for the neutral state).

**Fig. 5:**
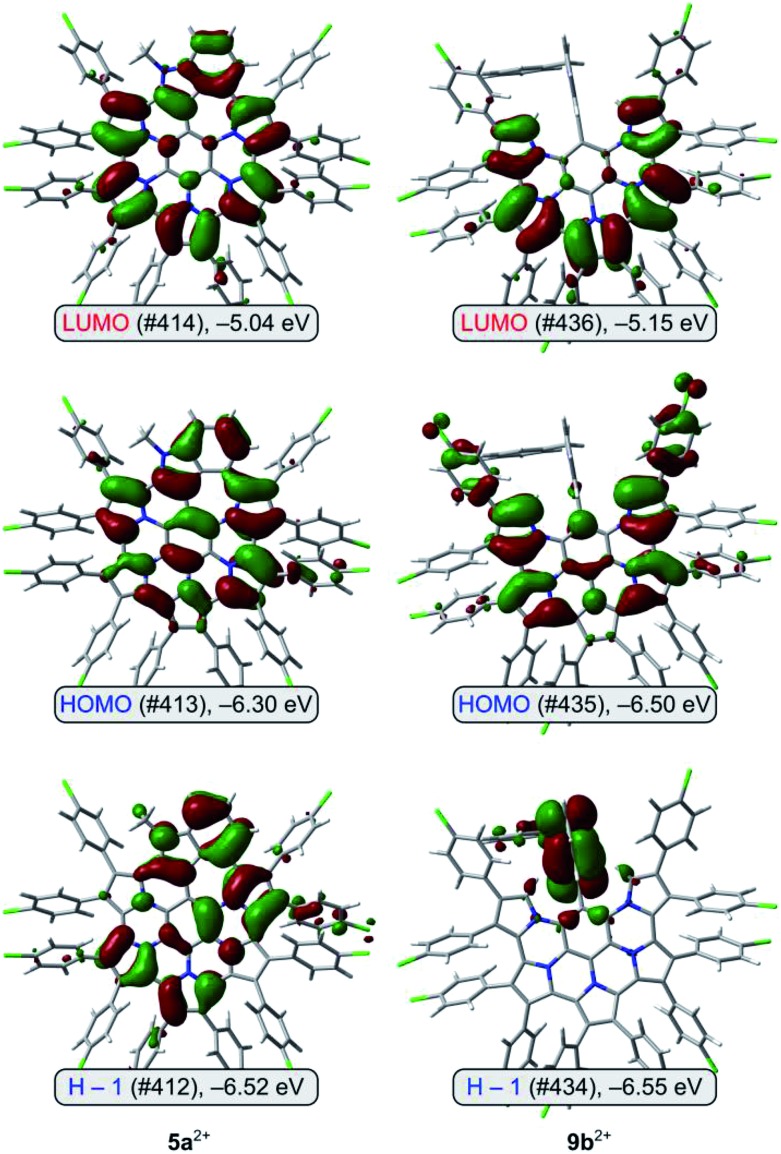
Frontier molecular orbitals for **5a^2+^** and **9b^2+^** (PCM(DCM)/TD-B3LYP/6-31G(d,p)).

Molecular orbital diagrams obtained for **9b^2+^** reveal that the highest occupied levels, HOMO and H–1, are very close in energy (Fig. 5). The HOMO orbital is localized on the nanographene core and 9,26-Ar groups, whereas H–1 has the highest coefficients on the non-fused indole unit. No such HOMO localization is seen in the contiguously conjugated **5a^2+^**. The lowest energy transition predicted by TD-DFT for **9b^2+^** is found to consist almost exclusively of the H–1 to LUMO excitation (for a similar case explored by TD-DFT see ref. 58). Because the latter orbital is localized on the nanographene, the transition has a considerable intramolecular charge-transfer (ICT) character,^59^ leading to the unexpectedly low optical band gap of **9b^2+^**.

**Fig. 6:**
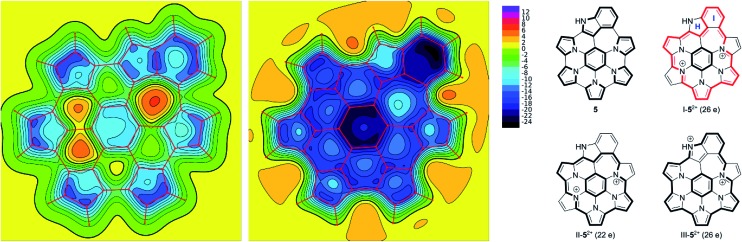
XY NICS(1) maps evaluated for **5** and **5^2+^** (left and right, respectively). GIAO shieldings (shown with negative values) were calculated at the B3LYP/6-31G(d,p) level of theory 1 Å above the molecular plane.

The effect of oxidation on the magnetic properties of **5** was visualized by probing GIAO shieldings 1 Å above the molecular plane (Fig. 6). The resulting NICS(1) maps^60^,^61^ show that in the neutral **5**, the overall shielding can be described as a superposition of the ring currents induced in the seven constituent subunits (five pyrroles, one benzene, and one indole). The outer subunits create a deshielding zone in the interior of the nanographene (rings B–G), partly cancelling the shielding effect above the central benzene ring A. In the dication, the NICS(1) scan revealed strongly negative values above the entire ring system, in line with the experimentally observed enhancement of diatropicity. The shielding magnitude is particularly large above the central ring A and the indole ring I, and noticeably weaker above rings B and H. Such a variation of shielding values indicates that the peripheral conjugation in **5^2+^** is dominated by 26-π-electron circuits, represented by the valence structure I-**5^2+^** (Fig. 6). These circuits include the indole ring I, while circumventing the peripheral nitrogen (ring H). Alternative circuit types II and III, which respectively exclude and include the entire indole moiety, apparently provide smaller contributions to the outer conjugation in **5^2+^**. The conjugation model proposed here is analogous to the typical description of macrocyclic aromaticity in oligopyrroles.^62^ In analogy to the trends found in the latter family of compounds, it may be proposed that the reduction of optical band gap, observed for **5a^2+^**, is linked to the expansion of the peripheral circuit in **5^2+^** (26 electrons) in comparison with the analogous circuit in **1^2+^** (22 electrons). The closest structural analogy for this change can be found in cyclo[6]- and cyclo[7]pyrrole,^63^ which contain conjugation pathways isoelectronic with those in **1^2+^** (22 e) and **5^2+^** (26 e), respectively. In these cyclopyrroles, peripheral expansion was observed to reduce the optical band gap from 1.57 to 1.32 eV.

The diatropic ring current associated with conjugation pathway I should produce significant downfield shifts of peripheral protons (notably those attached to ring I), as observed experimentally for **5a^2+^**. It can also be proposed that the upfield ^13^C shifts of ring-A carbons originate in part from the diatropic shielding contribution. By altering the effective bond orders, peripheral contributions such as I–III are expected to increase pyrrolic C_α_–C_β_ distances while reducing the C_β_–C_β_, C_α_–N and interpyrrolic C_α_–C_α_ bond lengths. Such changes are indeed consistently observed in the DFT-optimized geometries of **5a** and **5a^2+^**, with intermediate values of bond lengths observed for **5a^+^** (Table 1 and Fig. S21–S23†).

## Conclusions

The synthesis of the 5-6-7 nanographene presented herein shows that complex heteroaromatic fusion patterns can be accessed by direct oxidative coupling of judiciously designed precursors. The extension of the HPHAC motif, comprising a 7-membered ring and a peripheral benzo unit, results in a considerable red shift of fluorescence emission in the neutral state and an analogous shift of the NIR absorption in the cationic states. The NIR emission of expanded nanographenes, coupled with their redox activity, might become an attractive feature in the context of potential applications, *e.g.* in the design of fluorescence probes,^64^,^65^ provided that these systems can be tuned to provide substantially higher fluorescence quantum yields. The changes of optical properties can be linked to the aromatic conjugation along the edge of the nanographene, which is specifically expanded by the addition of peripheral rings, and which produces a distinct ring current in the dicationic state. These observations clearly indicate that expansion of electron-rich heteroaromatic nanographenes is an efficient strategy for designing low-bandgap chromophores and fluorophores.

## Supplementary Material

Supplementary information

Supplementary information
