# Supplementary material for: Synthesis of a peripherally conjugated 5-6-7 nanographene
Source: Chem Sci. 2015 Sep 29;7(1):286–94. doi: 10.1039/c5sc03280f (PMC5952525; doi:10.1039/c5sc03280f)
Supplement: Supplementary file 2 [file SC-007-C5SC03280F-s002.pdf]

Electronic Supplementary Material (ESI) for Chemical Science.
This journal is © The Royal Society of Chemistry 2015

Synthesis of a Peripherally Conjugated 5-6-7 Nanographene

by Marika Żyła, Elżbieta Gońka, Piotr J. Chmielewski, Joanna Cybińska, and Marcin Stępień*

Supporting Information

Table of contents

Experimental details........page S2

Synthetic procedures........page S4

Additional figures........page S10

Additional tables........page S34

^{1}H and ^{13}C NMR spectra........page S44

Mass spectra........page S53

Experimental

General. Tetrahydrofuran and N,N-dimethylformamide were dried using a commercial solvent purification system. Dichloromethane was distilled from calcium hydride when used as a reaction solvent. All other solvents and reagents were used as received. ^{1}H NMR spectra were recorded on high-field spectrometers (^{1}H frequency 500.13 or 600.13 MHz), equipped with broadband inverse gradient probeheads. Spectra were referenced to the residual solvent signals (chloroform-*d*, 7.24 ppm, toluene-d_8 2.09 ppm, dichloromethane-d_2 5.32 ppm). Two-dimensional NMR spectra were recorded with 2048 data points in the t_2 domain and up to 2048 points in the t_1 domain, with a 1 s recovery delay. All 2D spectra were recorded with gradient selection, with the exception of NOESY and ROESY. NOESY mixing time and ROESY spinlock time were 500 ms and 300 ms, respectively. ^{13}C NMR spectra were recorded with ^{1}H broadband decoupling and referenced to solvent signals ($^{13}CDCl_3$, 77.0 ppm, $^{13}CD_2Cl_2$, 53.5 ppm). In several systems exhibiting conformational dynamics, some signals were too broad to be observed in ^{13}C NMR spectra. High resolution mass spectra were recorded using ESI ionization in the positive mode. Electrochemical measurements (CH_2Cl_2, 0.1M TBAP) were performed on an EA9C Multifunctional Electrochemical Analyzer using a glassy-carbon working electrode, platinum wire as the auxiliary electrode, and silver wire as a reference electrode. The voltammograms were referenced against the halfwave potential of Fc/Fc^{+}. ESR spectra were obtained at room temperature with 9.76 GHz microwave radiation frequency (X-band) and recorded by means of 0.5-0.7 G signal modulation amplitude and 100 kHz modulation frequency.

Photoluminescence. Photoluminescence excitation (PLE) spectra as well as decay kinetics (DEC) were taken with the FSL980-sm Fluorescence Spectrometer from Edinburgh Instruments Ltd. A 450 W Xenon arc lamp (PL and PLE) and a Super Continuum Fianium laser were used as excitation sources. Emission spectra were corrected for the recording system efficiency and excitation spectra were corrected for the incident light intensity. Quantum yield measurements were performed by using an Edinburgh Instruments integrating sphere equipped with a small elliptical mirror and a baffle plate for beam steering and shielding against directly detected light. For the measurement, the integrating sphere replaces the standard sample holder inside the sample chamber. Calculations of quantum yields were made using the software provided by Edinburgh Instruments.

Computational methods. Density functional theory (DFT) calculations were performed using Gaussian 09.[1] DFT geometry optimizations were carried out in unconstrained C_1 symmetry, using molecular mechanics or semiempirical models as starting geometries. DFT geometries were refined to meet standard convergence criteria, and the existence of a local minimum was verified by a normal mode frequency calculation. DFT calculations were performed using the hybrid functional B3LYP,[2–4] the 6-31G(d,p) basis set. PCM solvation[5] was included in the calculations performed for aryl substituted structures (chloroform or dichloromethane). ^{1}H and ^{13}C shieldings were calculated using the GIAO approach and referenced to the absolute TMS shieldings calculated at the same level of theory (31.7532 ppm for ^{1}H and 191.8646 ppm for ^{13}C). The calculated shifts were averaged to the observed spectral symmetry before being compared with the experimental values. NICS calculations were performed for the geometry-optimized structures **5** and **5**$^{2+}$ using in-vacuo isotropic GIAO shieldings. Shielding values were probed using an array of Bq atoms set over a square grid with 151 × 151 points. The grid was placed 1 Å above the molecular plane. Electronic transitions were calculated by means of time-dependent DFT (TD-DFT),[1] using the B3LYP functional and dichloromethane

solvation. Up to 50 transitions were calculated, providing full coverage of the NIR and visible regions, and, in many cases, also of the UV region.

Synthesis

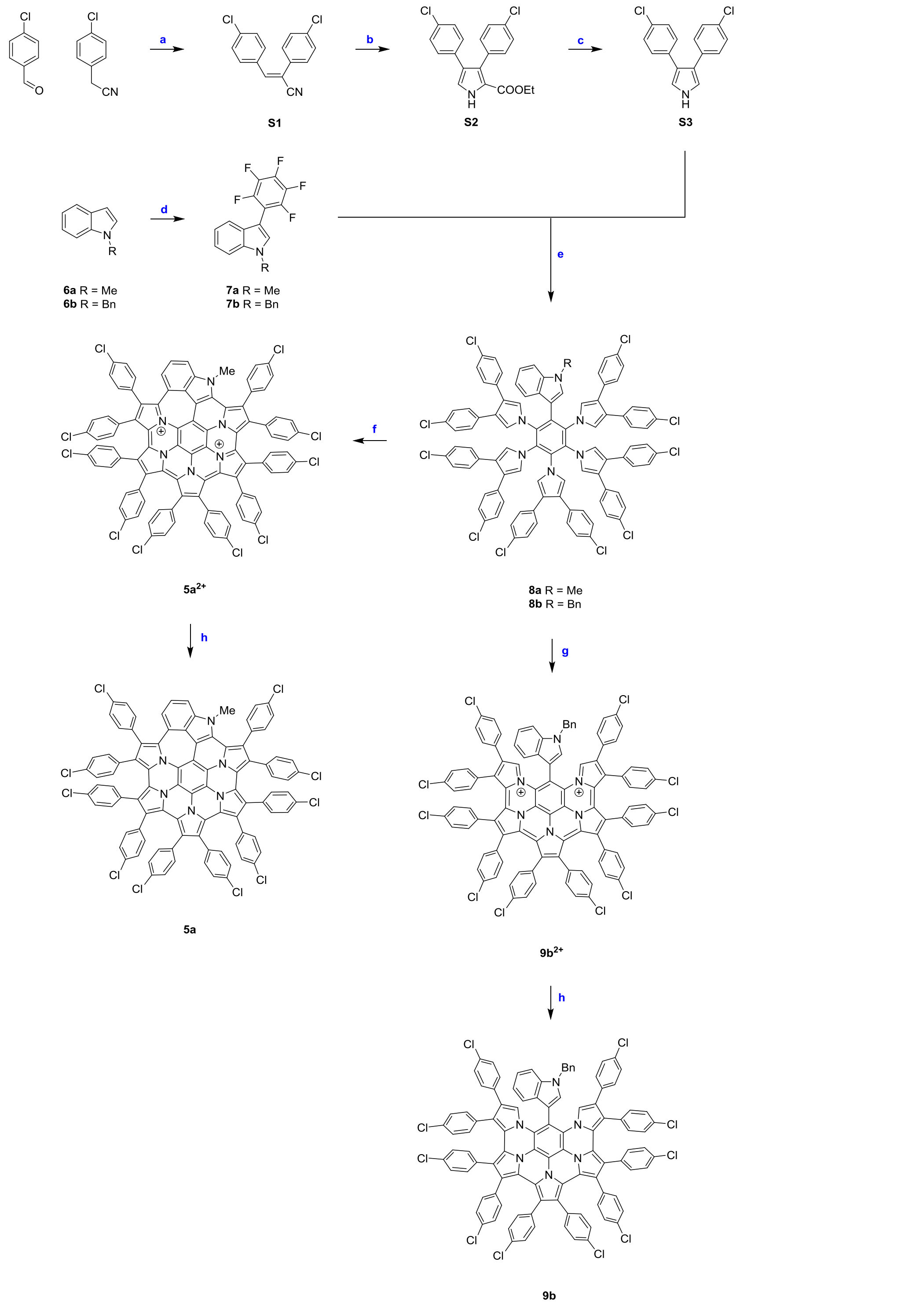

Scheme S1. Synthesis of **5a** and **9b**. Reagents and conditions: **(a)** EtONa (1.1 ml/mmol), EtOH; **(b)** $CNCH_2COOEt$ (1.0 equiv), *t*-BuOK (1.8 equiv), THF; **(c)** KOH (2.0 equiv), ethylene glycol; **(d)** Ag_2CO_3 (1.5 equiv), $Pd(OAc)_2$ (cat.), AcOH (1.0 equiv), pentafluorobenzene (3.0 equiv), DMF/DMSO; **(e)** NaH (5.0 equiv), DMF; **(f)** tris(4-bromophenyl)ammoniumyl hexachloroantimonate (BAHA, 12.0 equiv), diethyl ether – THF; **(g)** BAHA, (10.0 equiv), diethyl ether – THF; **(h)** zinc amalgam or Zn powder, CH_2Cl_2 or $CDCl_3$.

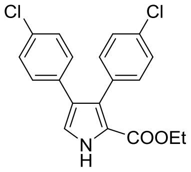

Ethyl 3,4-bis(4-chlorophenyl)-1*H*-pyrrole-2-carboxylate (S2). In a 100 mL round bottomed flask equipped with a magnetic stirring bar, (*Z*)-2,3-bis(4-chlorophenyl)acrylonitrile[6] (**S1**, 3.00 g, 10.7 mmol) and potassium *tert*-butoxide (2.17 g, 19.3 mmol) were dissolved in tetrahydrofuran (21.0 mL) and the mixture was stirred and cooled to 0 °C under nitrogen. Ethyl isocyanoacetate (1.17 mL, 10.7 mmol) was added and the mixture was allowed to warm to room temperature. The mixture was stirred under nitrogen for 5 days, and was subsequently treated with excess water and extracted with dichloromethane. The combined organic extracts were dried over sodium sulfate, filtered and evaporated under reduced pressure. The residue was subjected to column chromatography (silica gel, dichloromethane). The desired fraction was collected and stripped of solvent under reduced pressure, yielding an orange oil (1.68 g, 43%). **^{1}H NMR** (500 MHz, chloroform-*d*, 300 K): δ 9.22 (s, 1H), 7.25 (dt, 2H, ^3J = 8.6 Hz), 7.16 (m, 4H), 7.05 (d, 1H, ^3J = 3.0 Hz), 6.98 (dt, 2H, ^3J = 8.4 Hz), 4.18 (q, 2H, ^3J = 7.1 Hz), 1.16 (t, 3H, ^3J = 7.0 Hz). **^{13}C NMR** (125 MHz, chloroform-*d*, 300 K): δ 161.23, 133.17, 132.91, 132.76, 132.35, 132.32, 129.72, 128.66, 128.04, 128.01, 125.75, 120.60, 120.43, 60.67, 14.29. **HRMS** (ESI+): *m/z*: $[M + H]^+$ Calcd. for $C_{19}H_{15}Cl_2NO_2$: 358.0396; Found 358.0398.

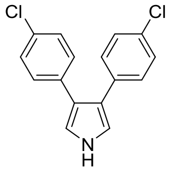

3,4-Bis(4-chlorophenyl)-1*H*-pyrrole (S3). Compound **S2** (1.68 g, 4.7 mmol), potassium hydroxide (0.53 g, 9.3 mmol) and ethylene glycol (4.0 mL) were placed in a 50 mL round bottomed flask equipped with a magnetic stirring bar. The mixture was purged with nitrogen for 1 h. The mixture was heated to 190 °C and stirred under nitrogen for 4 h. Subsequently, the mixture was allowed to cool to room temperature, treated with excess water and brine, and extracted with dichloromethane. The combined organic extracts were dried over sodium sulfate, filtered and evaporated under reduced pressure, yielding brown crystals (1.31 g, 96%). **^{1}H NMR** (600 MHz, chloroform-*d*, 300 K): δ 8.32 (s, 1H), 7.21 (d, 4H, ^3J = 8.5 Hz), 7.14 (d, 4H, ^3J = 8.4 Hz), 6.87 (d, 2H, ^3J = 2.6 Hz). **^{13}C NMR** (150 MHz, chloroform-*d*, 300 K): δ 134.17, 131.95, 129.95, 128.67, 122.71, 117.81. **HRMS** (ESI+): *m/z*: $[M + H]^+$ Calcd. for $C_{16}H_{11}Cl_2N$: 286.0185; Found 286.0173.

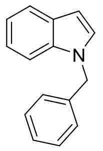

1-Benzyl-1*H*-indole (6b). Obtained according to a literature procedure.[7] **^{1}H NMR** (500 MHz, chloroform *d*, 298 K): δ 7.63 (d, 1H, ^2J = 7.9 Hz), 7.29-7.23 (m, 4H), 7.15 (t, 1H, ^2J = 8.1 Hz), 7.12-7.07 (m, 4H), 6.54 (d, 1H, ^3J = 3.1 Hz), 5.32 (s, 2H).

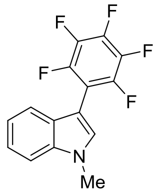

1-Methyl-3-(perfluorophenyl)-1*H*-indole (7a). Obtained according to a literature procedure.[8] **^{1}H NMR** (500 MHz, chloroform-*d*, 298 K): δ 7.50 (d, 1H, ^2J = 8.1 Hz), 7.38 (d, 1H, ^2J = 8.2 Hz),7.30 (t, 1H, ^2J = 7.8 Hz),7.28 (s, 1H),7.20 (t, 1H, ^2J = 7.1 Hz), 3.87 (s, 3H).

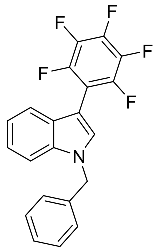

1-Benzyl-3-(perfluorophenyl)-1*H*-indole (7b). In a 20 mL septum-capped pressure tube equipped with magnetic stirring bar were placed **6b** (175.0 mg, 0.827 mmol), silver carbonate (342.2 mg, 1.24 mmol), and palladium(II) acetate (13.9 mg, 0.021 mmol, 2.5 mol%) under N_2, followed by dry N,N-dimethylformamide (10.0 mL), dimethyl sulfoxide (0.50 mL), and glacial acetic acid (50.0 µL, 0.864 mmol). Subsequently, pentafluorobenzene was added to the mixture. The tube was then screw-capped and heated to 120 °C. After stirring overnight, the reaction mixture was filtred through a celite pad, washed out with dichloromethane, and concentrated on a rotary evaporator. The residue was purified using silica gel chromatography to provide the pure product with 50% yield (154.0 mg, yellowish solid). **^{1}H NMR** (600 MHz, chloroform-*d*, 300 K): δ 7.53 (d, 1H, ^2J = 7.9 Hz), 7.36 – 7.16 (m, 9H), 5.38 (s, 2H). **^{13}C NMR** (151 MHz, chloroform-*d*, 300 K): δ 145.05, 143.42, 140.36, 138.81, 138.68, 137.14, 136.52, 136.45, 129.44, 128.94 (x2), 127.97, 126.95 (x2), 126.63, 122.61 (x2), 120.69, 120.43, 110.20, 50.47. **HRMS** (ESI-TOF): *m/z*: $[M + H]^+$ Calcd. for $C_{21}H_{12}F_5N$: 374.0963; Found 374.0968.

General procedure for the synthesis of hexaarylbenzenes 8a and 8b. Compound **S3** (5.0 equiv) and sodium hydride (60% in mineral oil, 5.0 equiv) were placed in a 10 mL round bottomed flask equipped with a magnetic stirring bar. The mixture was cooled to 0 °C and N,N-dimethylformamide was added. After stirring for 45 minutes under nitrogen with further cooling, perfluorophenyl derivative (**8a** or **8b**, 1.0 equiv) was slowly added and the reaction mixture was heated to 50 °C. Stirring was continued overnight under inert atmosphere and then the reaction mixture was quenched diluted with water and small amount of brine, and extracted with dichloromethane. The combined organic layers were dried over anhydrous sodium sulfate, filtred and evaporated under reduced pressure.

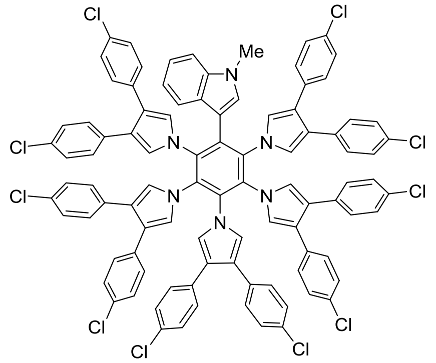

1-Methyl-3-(2,3,4,5,6-pentakis(3,4-bis(4-chlorophenyl)-1*H*-pyrrol-1-yl)phenyl)-1*H*-indole (8a). Prepared *via* general procedure using **7a** (84.0 mg, 0.282 mmol), in N,N-dimethylformamide (3.0 mL), **S3** (407.0 mg, 1.41 mmol), and NaH (56.5 mg, 1.41 mg) in DMF (7.0 mL). The crude product was purified on column chromatography (silica gel, dichloromethane/hexanes 1:1). The first fraction was collected and concentrated under reduced pressure. Product was obtained as pale yellow crystals (289.0 mg, 62%). **^{1}H NMR** (600 MHz, chloroform-*d*, 300 K): δ 7.40 (d, 1H, ^{2}J = 8.4 Hz), 7.26 - 7.23 (m, 1H), 7.19 (d, 5H, ^{2}J = 8.5 Hz), 7.17 (d, 7H, ^{2}J = 8.5 Hz), 7.11 (d, 8H, ^{2}J = 8.4 Hz), 6.98 – 6.88 (m, 14H), 6.73 – 6.71 (m, 9H), 6.50 (s, 2H), 6.47 (s, 4H), 6.42 (s, 1H), 3.77 (s, 3H). **^{13}C NMR** (chloroform-*d*, 151 MHz, 300 K): δ 136.60, 136.15, 132.78, 132.73, 132.71, 132.64, 132.54, 132.51, 132.34, 132.07, 131.80, 129.40 (x3), 128.80, 128.75, 128.53, 128.38, 126.90, 125.11, 124.95, 124.16, 122.84, 121.07, 120.46, 120.39, 120.36, 118.59, 109.29, 106.49, 33.19. **HRMS** (ESI-TOF): *m/z*: $[M]^+$ Calcd. for $C_{95}H_{59}Cl_{10}N_6$: 1633.17; Found 1633.18.

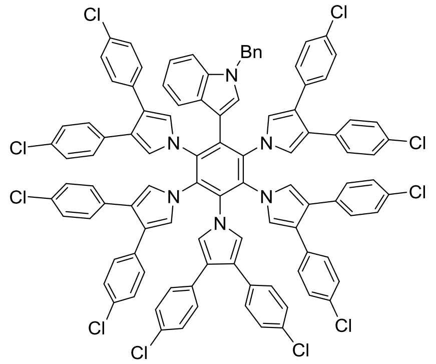

1-Benzyl-3-(2,3,4,5,6-pentakis(3,4-bis(4-chlorophenyl)-1H-pyrrol-1-yl)phenyl)-1H-indole (8b). Prepared *via* general procedure using **7b** (31.4 mg, 0.084 mmol) in 1.50 mL N,N-dimethylformamide and **S3** (121.1 mg, 0.420 mmol), and sodium hydride (16.8 mg, 0.420 mmol) in 1.50 mL of solvent. The crude product was purified on column chromatography (silica gel, dichloromethane/hexanes 1:1). The first fraction was collected and stripped of solvent on rotary evaporator. Subsequently, the product was recrystallized from CH_2Cl_2/*n*-hexane yielding yellow crystals. (91 mg, 63%). **^{1}H NMR** (600 MHz, chloroform-*d*, 300 K): δ 7.33 (d, 1H, ^{2}J = 8.2 Hz), 7.25 (d, 1H, ^{2}J = 7.9 Hz), 7.24 (m, 1H), 7.22 – 7.14 (m, 14H), 7.13 – 7.06 (m, 10H), 7.02 (d, 2H, ^{2}J = 7.2 Hz), 6.98 (d, 4H, ^{2}J = 8.7 Hz), 6.94 (d, 8H, ^{2}J = 8.6 Hz), 6.92 – 6.90 (m, 2H), 6.72 (d, 7H, ^{2}J = 8.4 Hz), 6.51 (s, 2H), 6.48 (s, 4H), 6.46 (s, 4H), 5.27 (s, 2H) **^{13}C NMR** (151 MHz, chloroform-*d*, 300 K): δ 136.47, 136.42, 136.24, 133.06, 132.89, 132.87, 132.81, 132.65, 132.62, 132.50, 132.39, 131.93, 129.52, 129.48 (x2), 129.04, 128.83, 128.79, 128.57, 128.09, 127.75, 127.33, 126.62, 125.34, 125.17, 124.36, 123.12, 121.27, 120.72, 120.53, 120.51, 118.72, 109.95, 107.40, 50.63. **HRMS** (ESI): *m/z*: $[M]^+$ Calcd. for $C_{101}H_{62}Cl_{10}N_6$: 1708.2; Found 1708.1.

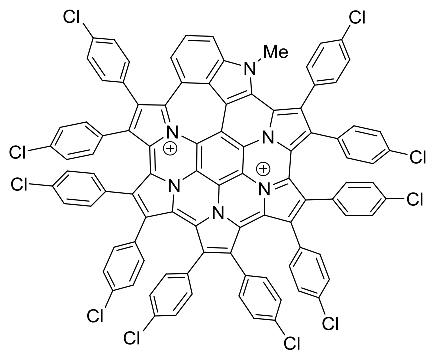

Compound 5a[SbCl$_6$]$_2$. In 5 mL round-bottom flask equipped with a magnetic stirring bar and protected from light, tris(4-bromophenyl)ammoniumyl hexachloroantimonate (59.0 mg, 0.073 mmol) was placed and then dry tetrahydrofuran (20.0 µL) was added. In a pear-shaped flask, compound **8a** (10.0 mg, 0.0061 mmol) was dissolved in Et_2O (3.0 mL) under nitrogen. The solution of **8a** was added to the oxidant in one portion and the mixture was stirred under N_2 for 30 minutes. The dark brown precipitate was formed and isolated. Subsequently, the crude product was recrystallized from DCM/*n*-hexane and rinsed with Et_2O, yielding dark brown crystals (62%, 8.7 mg) **^{1}H NMR** (600 MHz, acetonitrile-d_3, 300 K): δ 9.83 (d, 1H, ^2J = 8.2 Hz), 9.34 (d, 1H, ^2J = 8.2 Hz), 9.00 (t, 1H, ^2J = 7.9 Hz), 7.94 (d, 2H, ^2J = 8.1 Hz), 7.72 (d, 2H, ^2J = 8.4 Hz), 7.70 (d, 2H, ^2J = 8.4 Hz), 7.64 (d, 2H, ^2J = 8.5 Hz), 7.43 (d, 2H, ^2J = 8.0 Hz), 7.27 – 7.24 (m, 9H), 7.23 – 7.20 (m, 5H), 7.18 – 7.14 (m, 6H), 7.13 – 7.10 (m, 8H), 7.07 (d, 2H, ^2J = 8.2 Hz), 4.58 (s, 3H). **^{13}C NMR** (151 MHz, chloroform-*d*, 300 K): δ 144.37, 142.96, 141.29, 140.61, 139.68, 137.97, 137.53, 137.22, 136.92, 136.87, 136.26, 136.24, 136.08, 135.96, 135.60, 135.54, 135.10, 134.8, 134.76, 134.13, 133.29, 133.14, 132.86, 132.76, 132.72, 132.61, 132.57, 132.52, 132.45, 132.38, 132.21, 131.66, 131.27, 131.10, 130.02, 129.63 (x2), 129.59, 129.52, 129.40, 129.07 (x2), 128.72, 128.60, 128.55, 128.33, 128.17, 128.02, 127.21, 126.93, 125.51, 125.28, 123.78, 123.58, 123.50, 122.39, 121.81, 120.95, 120.54, 118.19, 113.79, 110.07, 109.73, 108.27, 103.08, 101.69, 99.76, 98.69, 37.04. **HRMS** (ESI-TOF): *m/z*: $[M]^{2+}$ Calcd. for $C_{95}H_{46}Cl_{10}N_6$: 810.0329; Found 810.0362. **UV-vis** data are given in Table 1.

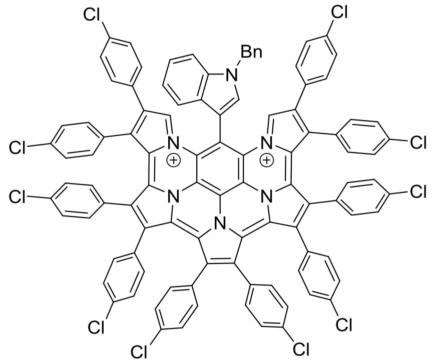

13-(1-Benzyl-1*H*-indol-3-yl)-2,3,4,5,6,7,8,9,10,11-decakis(4-chlorophenyl)-3b^1,5b^1,7b^1,12a,13b-pentaazatricyclopenta[*a*,*cd*,*fg*]-*as*-indaceno[3,4,5-*ijk*]pyrene-12*a*,13*b*-diium bis(hexachloroantimonate) (9b[SbCl$_6$]$_2$). In a 5 mL round-bottom flask equipped with a magnetic stirring bar and protected from light, tris(4-bromophenyl)ammoniumyl hexachloroantimonate (40.2 mg, 0.0492 mmol) was placed and then dry tetrahydrofuran (15.0 µL) was added. In a pear-shaped flask, compound **8b** (8.44 mg, 0.00492 mmol) was dissolved in Et_2O (3.0 mL) under nitrogen. The solution of **8b** was added to the oxidant in one portion. The mixture was stirred under N_2 for 30 minutes. The dark green precipitate was formed and isolated. Subsequently, the crude product was rinsed with *n*-hexane, yielding deep green crystals (42%, 4.9 mg). **^{1}H NMR** (600 MHz, chloroform-*d*,

260 K): δ 7.69 (s, 1H), 7.66 (m, 2H), 7.52 (t, 1H, ^2J = 8.0 Hz), 7.49 (s, 2H), 7.37 (t, 1H, ^2J = 7.7 Hz), 7.10 – 7.06 (m, 3H), 7.02 – 6.59 (m, 38H), 6.21 (d, 4H, ^2J = 8.2 Hz), 5.45 (s, 2H). **^{13}C NMR** (151 MHz, chloroform-*d*, 260 K): δ 145.88, 141.66, 140.65, 136.27, 136.03, 135.88, 135.68, 135.48, 135.29, 134.72, 133.94, 133.53, 132.39, 132.29, 132.09, 131.57, 131.51, 131.30, 131.23, 130.92, 130.40, 129.40, 129.37, 129.07, 129.00, 128.84, 128.74, 128.65, 128.52, 128.39, 128.33, 128.31, 128.25, 128.17, 128.08, 127.51, 127.27, 126.90, 126.60, 125.51, 124.10, 123.75, 115.82, 111.36, 111.31, 110.07, 109.93, 105.23, 51.33. **HRMS** (ESI-TOF): *m/z*: $[M-4H]^+$ (≡ **5b**$^+$) Calcd. for $C_{101}H_{50}Cl_{10}N_6$: 1696.0977; Found 1696.1016. **UV-vis** data are given in Table 1.

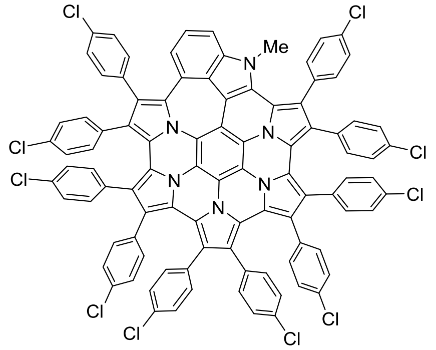

Compound 5a. Compound **5a**$[SbCl_6]_2$ was shaken well with small amount of zinc amalgam in freshly distilled dichlorometane solution until the color changed from chocolate brown to orange. **^{1}H NMR** (600 MHz, chloroform-*d*, 300 K): δ 7.16 (d, 2H, ^2J = 8.4 Hz), 7.13 (d, 2H, ^2J = 8.4 Hz), 7.01 (d, 2H, ^2J = 8.4 Hz), 6.97 (d, 2H, ^2J = 8.4 Hz), 6.74 – 6.47 (m, 23H), 6.36 – 6.26 (m, 12H).

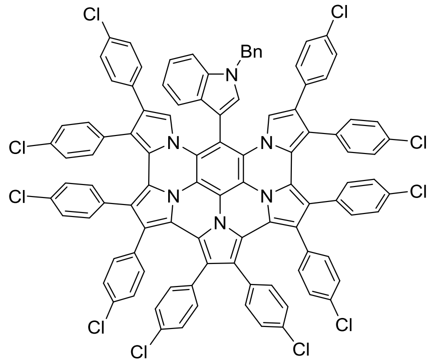

13-(1-Benzyl-1*H*-indol-3-yl)-2,3,4,5,6,7,8,9,10,11-decakis(4-chlorophenyl)-3b^1,5b^1,7b^1,12a,13b-pentaazatricyclopenta[*a*,*cd*,*fg*]-*as*-indaceno[3,4,5-*ijk*]pyrene (9b). Compound **9b$[SbCl_6]_2$** was shaken well with small amount of zinc amalgam in fresh distilled dichlorometane solution until the color changed from deep green to yellow. **^{1}H NMR** (600 MHz, chloroform-*d*, 260 K): δ 7.76 (d, 1H, ^2J = 8.6 Hz), 7.57 (d, 1H, ^2J = 8.6 Hz), 7.45 (t, 1H, ^2J = 7.8 Hz), 7.37 (s, 1H), 7.08 (d, 1H, ^2J = 7.7 Hz),6.98 (d, 2H, ^2J = 7.4 Hz),6.88 (d, 4H, ^2J = 8.6 Hz),6.80 (d, 4H, ^2J = 8.2 Hz),6.75 (d, 2H, ^2J = 7.7 Hz), 6.71 – 6.60 (m, 20H), 6.50 – 6.37 (m, 12H), 6.12 (d, 4H, ^2J = 8.6 Hz), 5.49 (s, 2H).

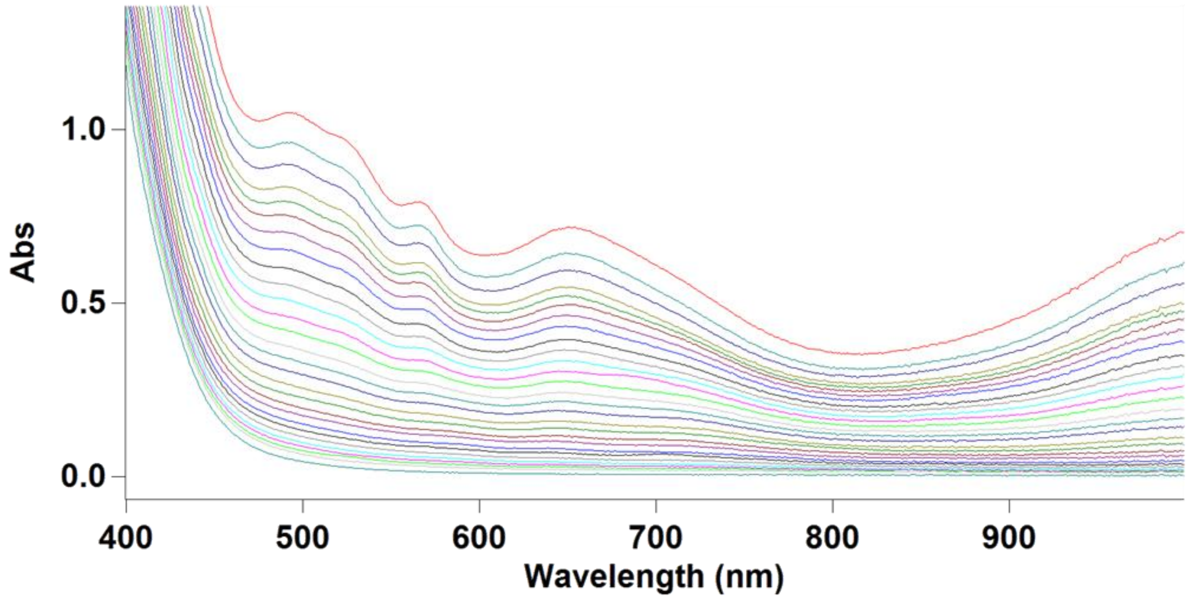

Figure S1. Spectrophotometric titration of **8a** with BAHA (dichloromethane). The increasing absorption corresponds to the formation of **5a**$^{2+}$.

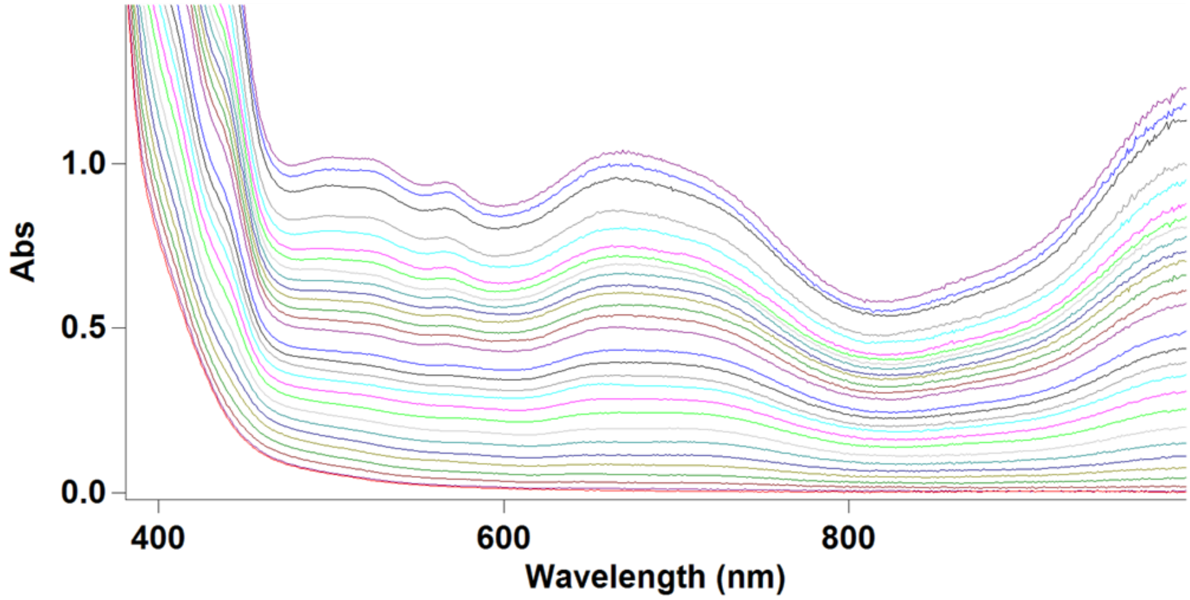

Figure S2. Spectrophotometric titration of **8b** with BAHA (dichloromethane). A mixture of **9b**$^{2+}$ and **5b**$^{2+}$ is formed throughout the titration.

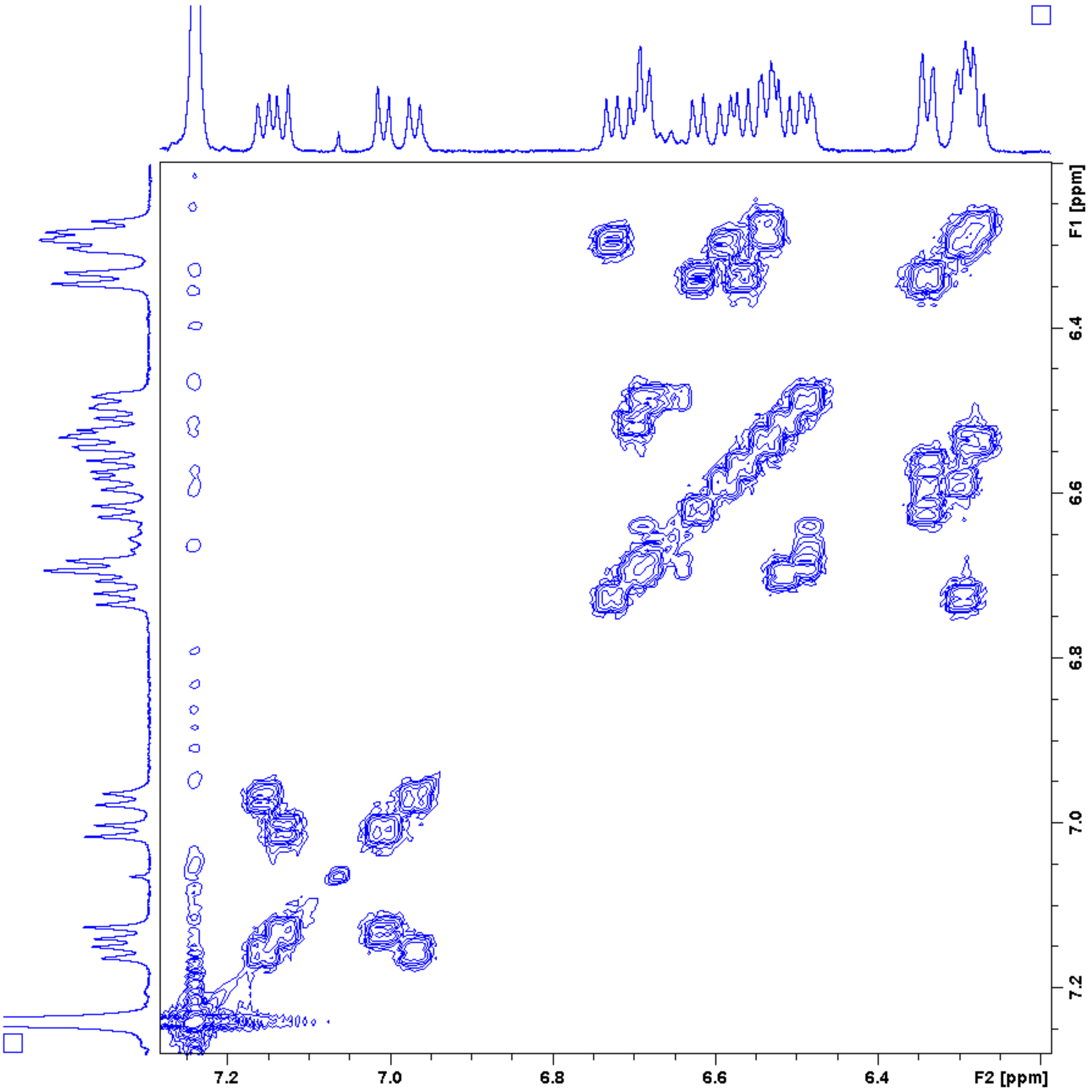

Figure S3. Partial ^{1}H COSY spectrum of **5a** (600 MHz, $CDCl_3$, 300 K).

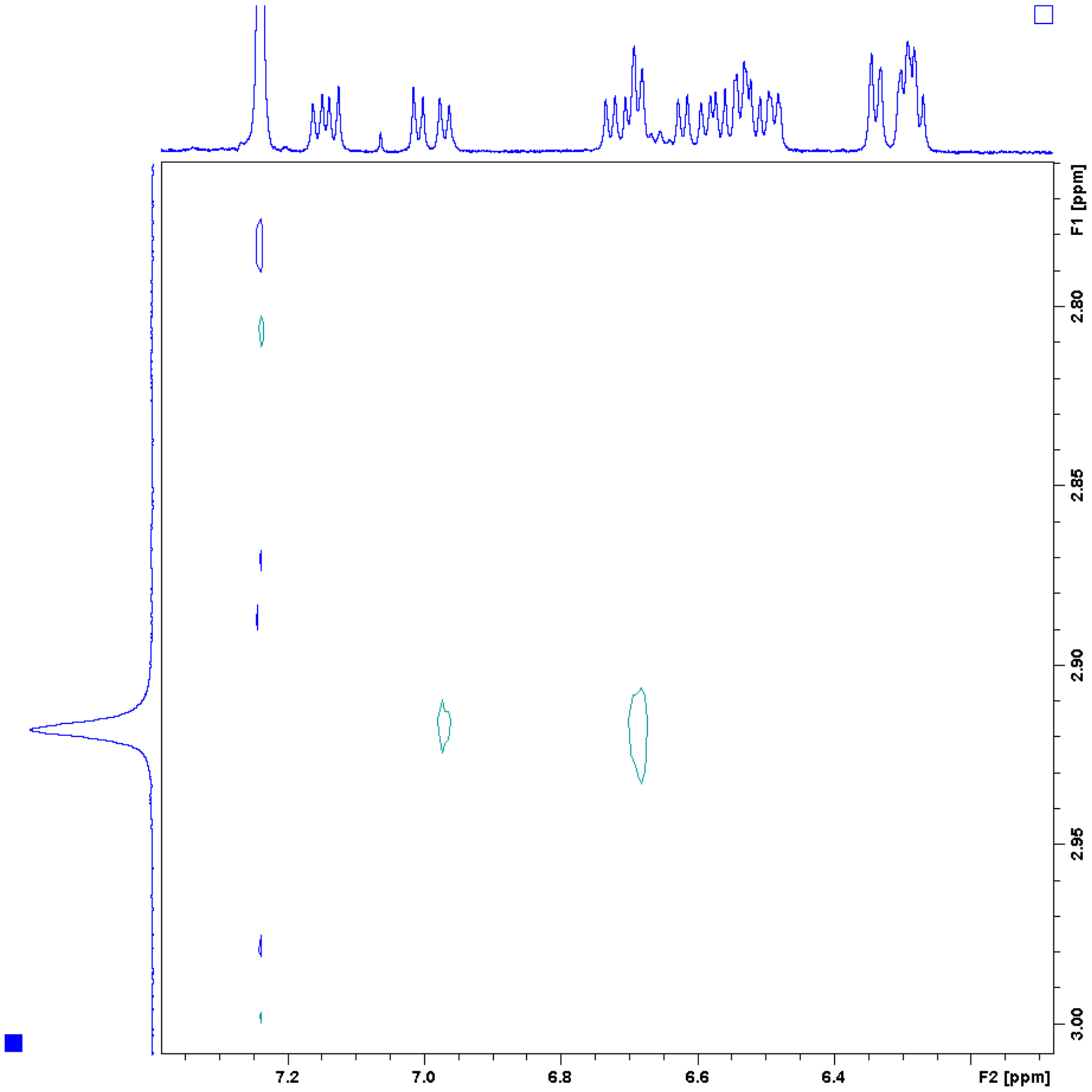

Figure S4. Partial ^{1}H ROESY spectrum of **5a** (600 MHz, $CDCl_3$, 300 K).

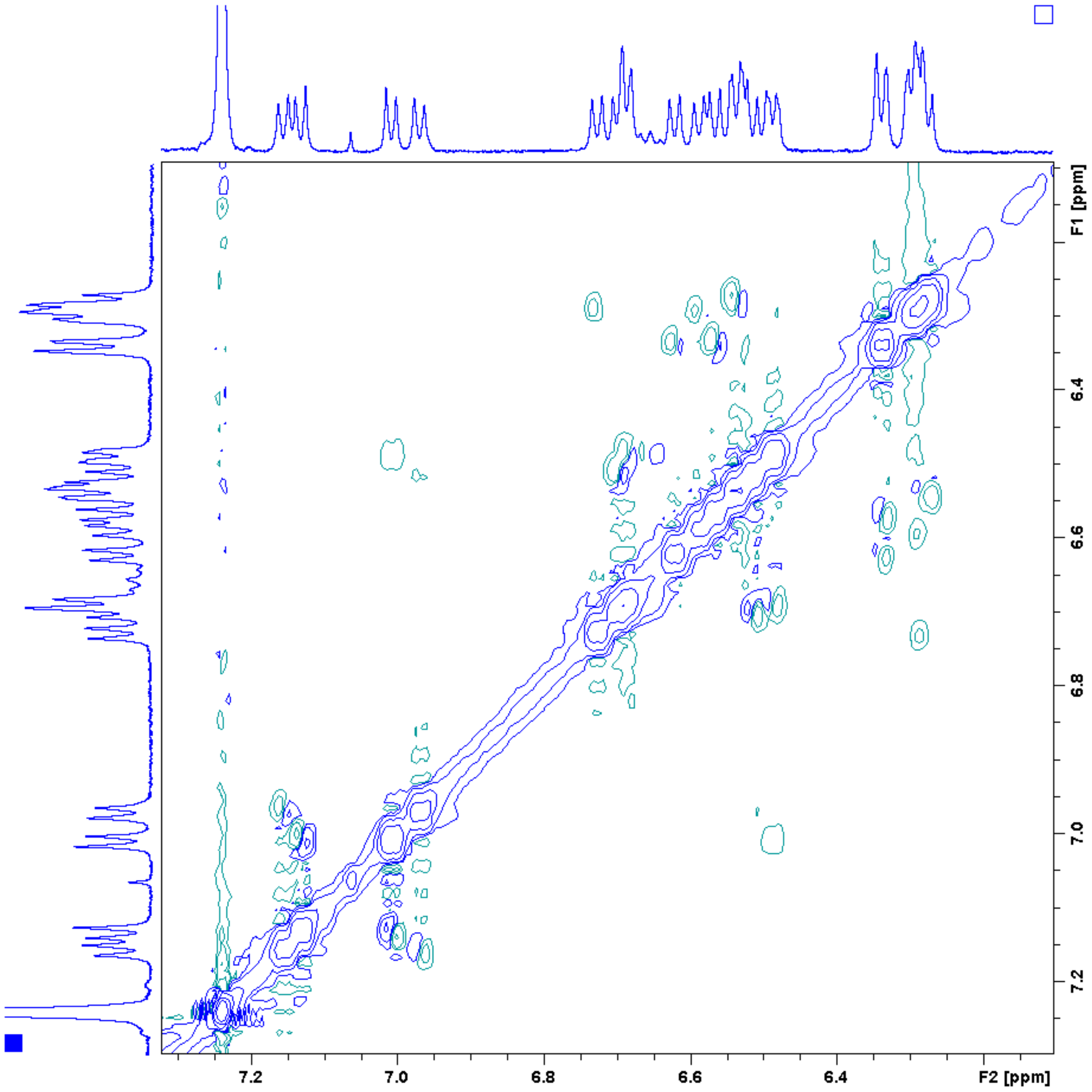

Figure S5. Partial ^{1}H ROESY spectrum of **5a** (600 MHz, $CDCl_3$, 300 K).

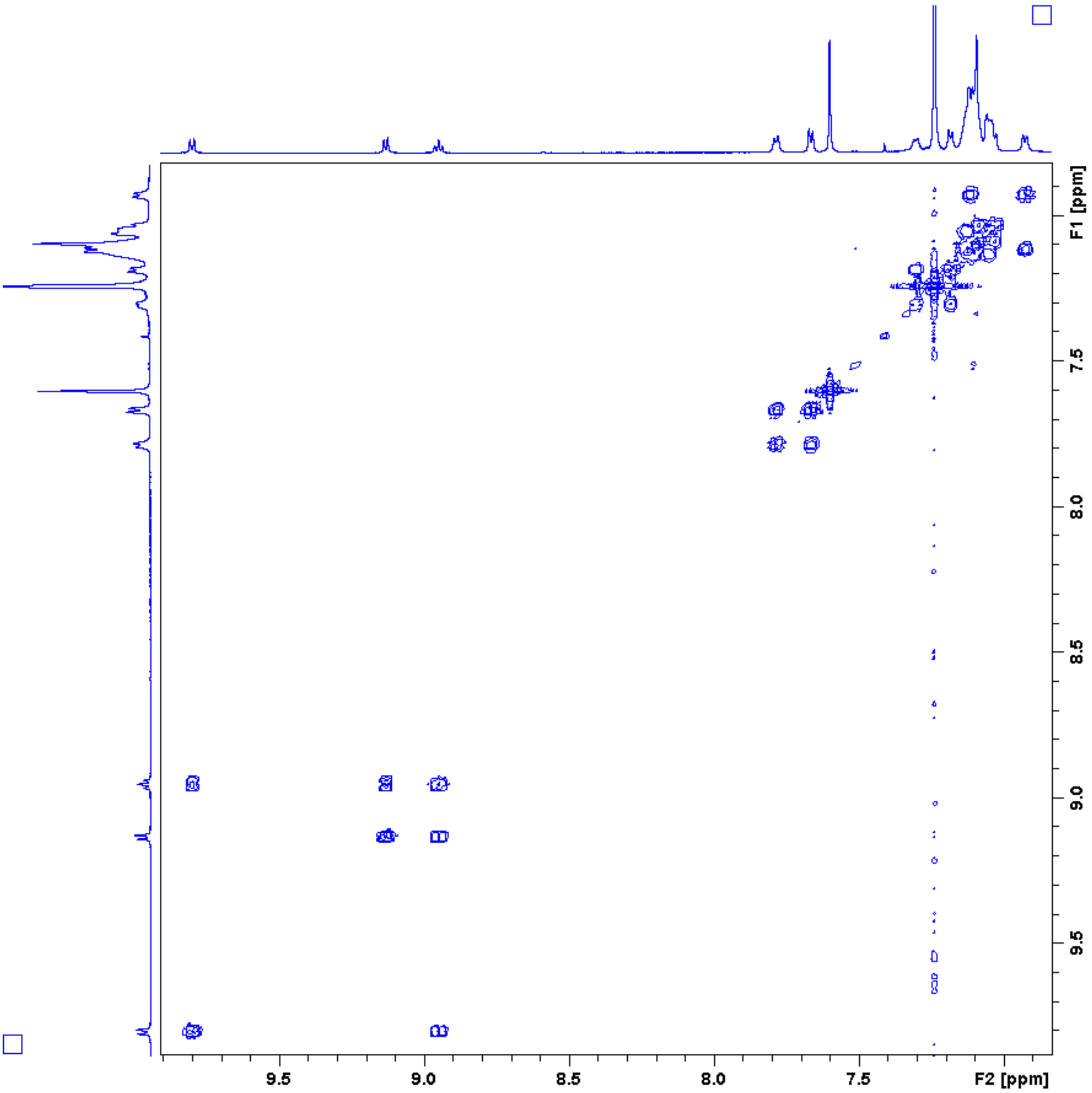

Figure S6. Partial ^{1}H COSY spectrum of **5a**$[SbCl_6]_2$ (600 MHz, $CDCl_3$, 300 K).

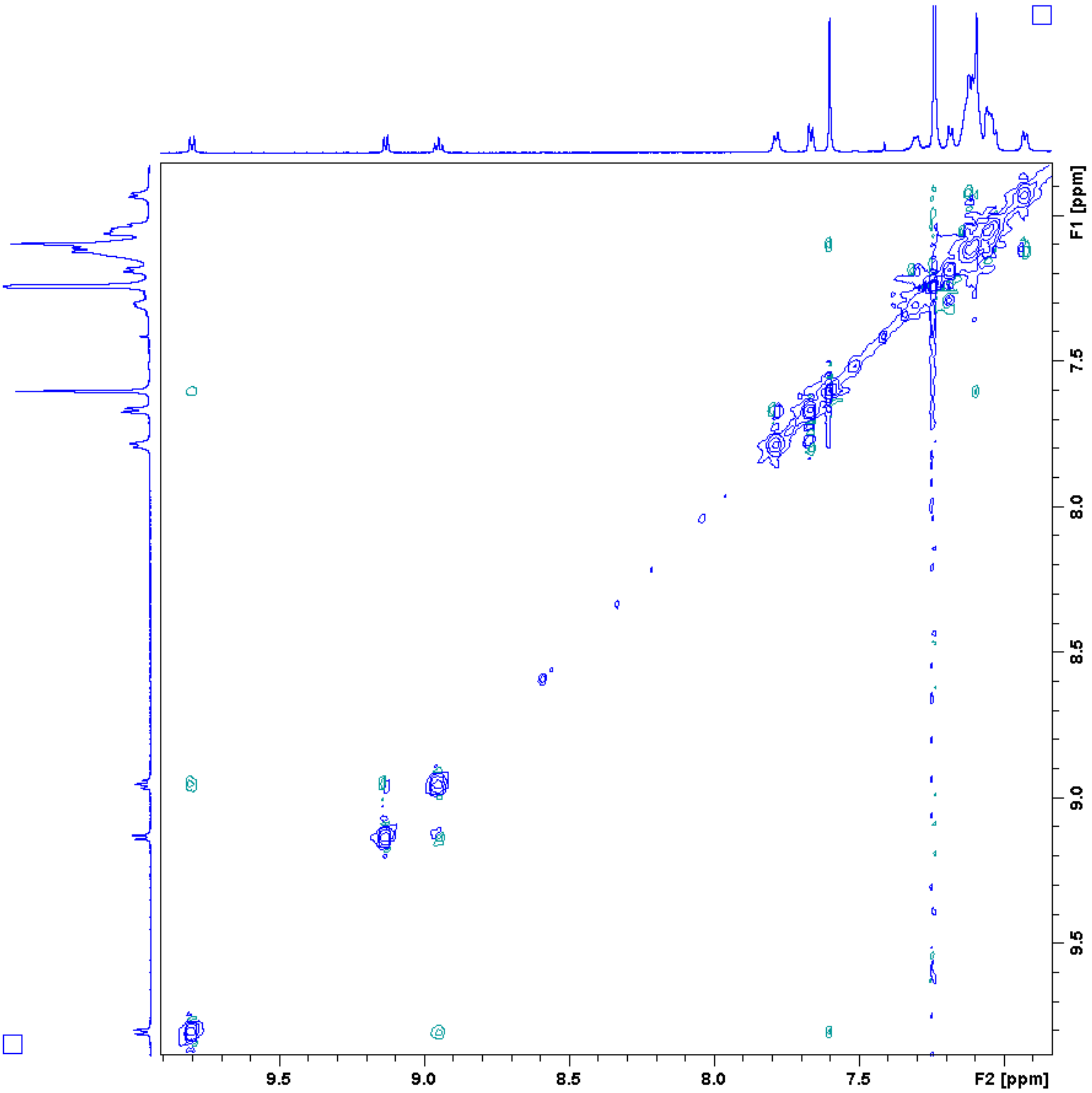

Figure S7. Partial ^{1}H ROESY spectrum of **5a**[SbCl$_6$]$_2$ (600 MHz, $CDCl_3$, 300 K).

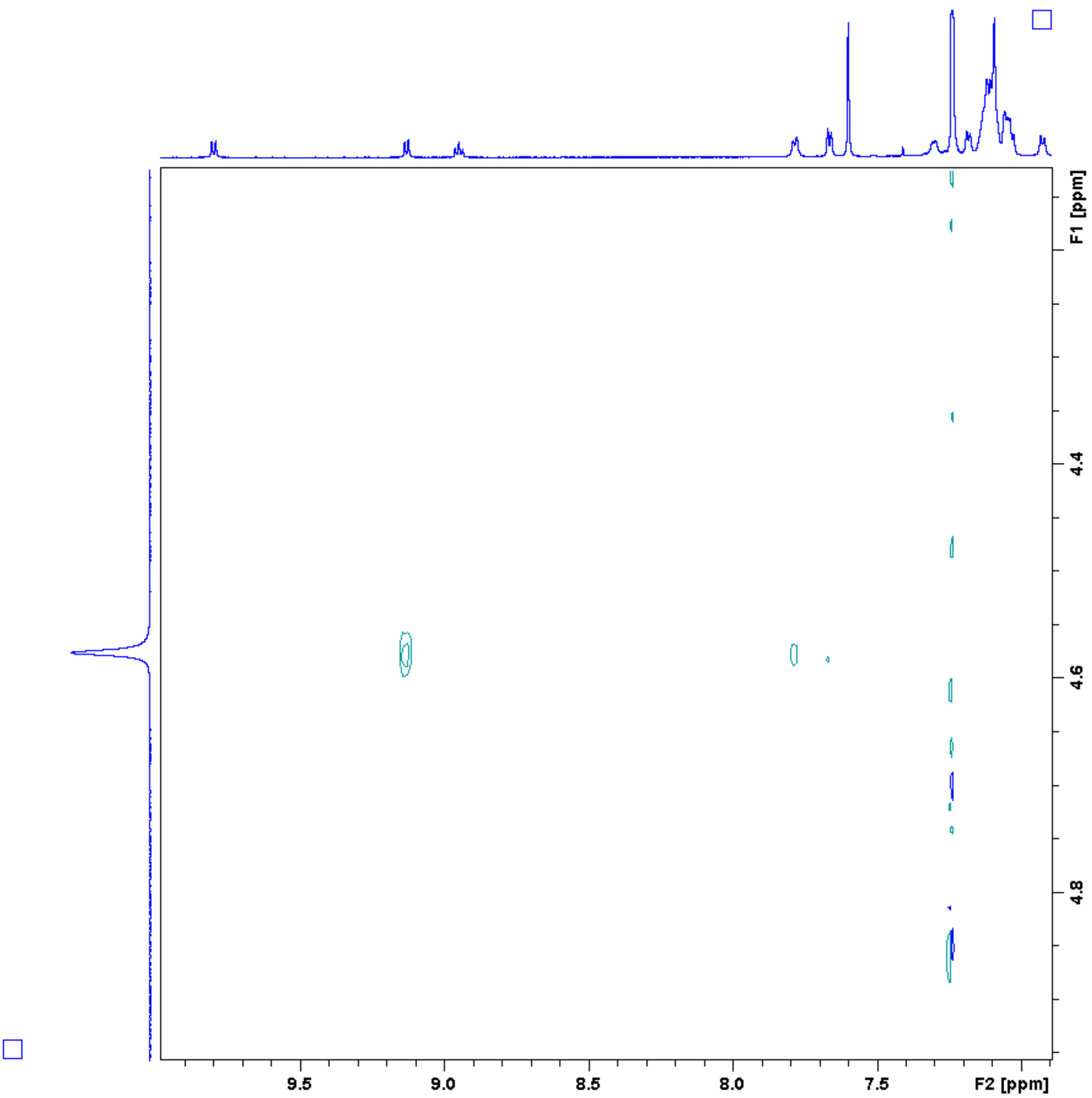

Figure S8. Partial ^{1}H ROESY spectrum of **5a**[SbCl$_6$]$_2$ (600 MHz, CDCl$_3$, 300 K).

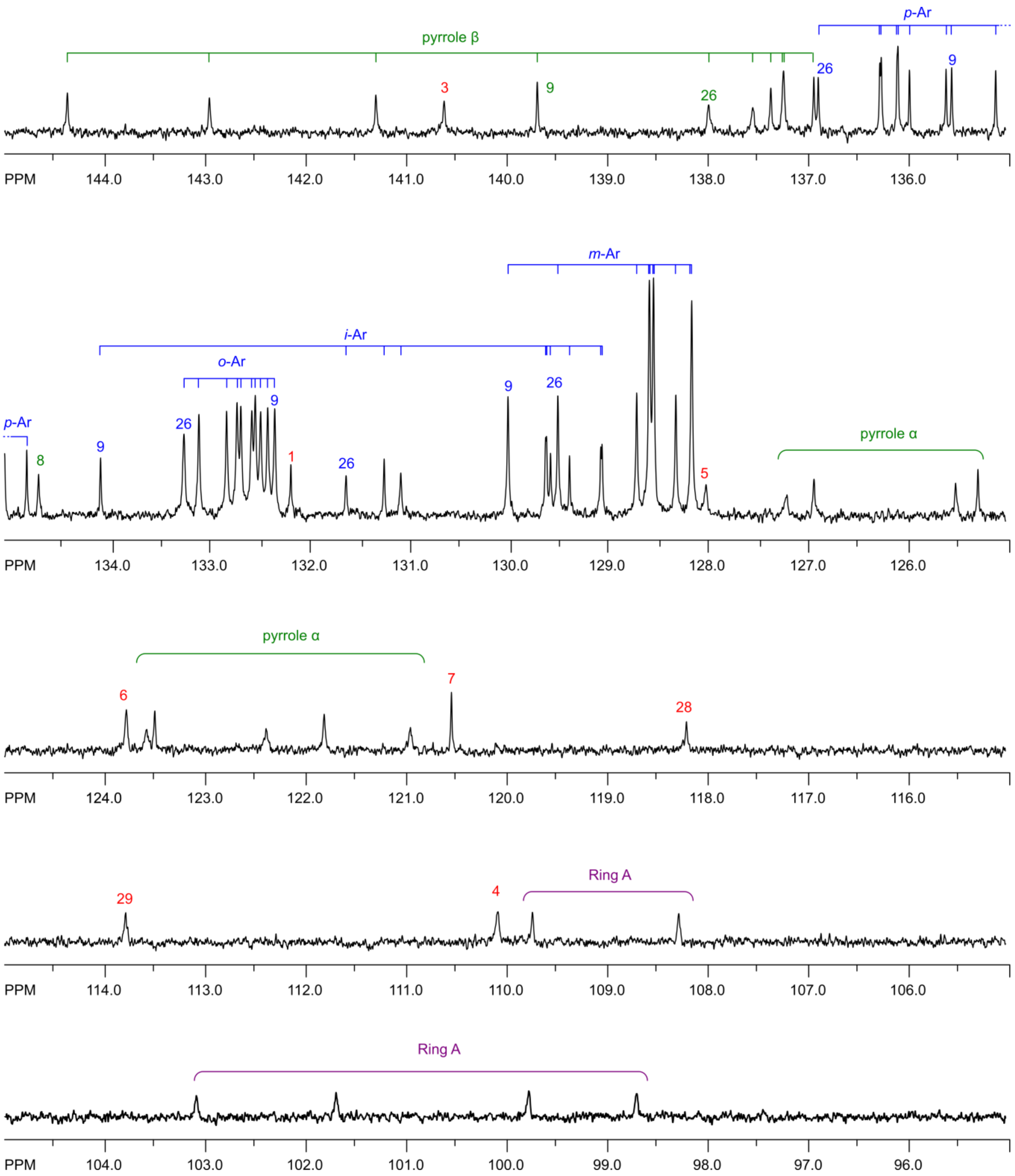

Figure S9. Assigned aromatic region of the ^{13}C NMR spectrum of **5a**$[SbCl_6]_2$ (600 MHz, $CDCl_3$, 300 K).

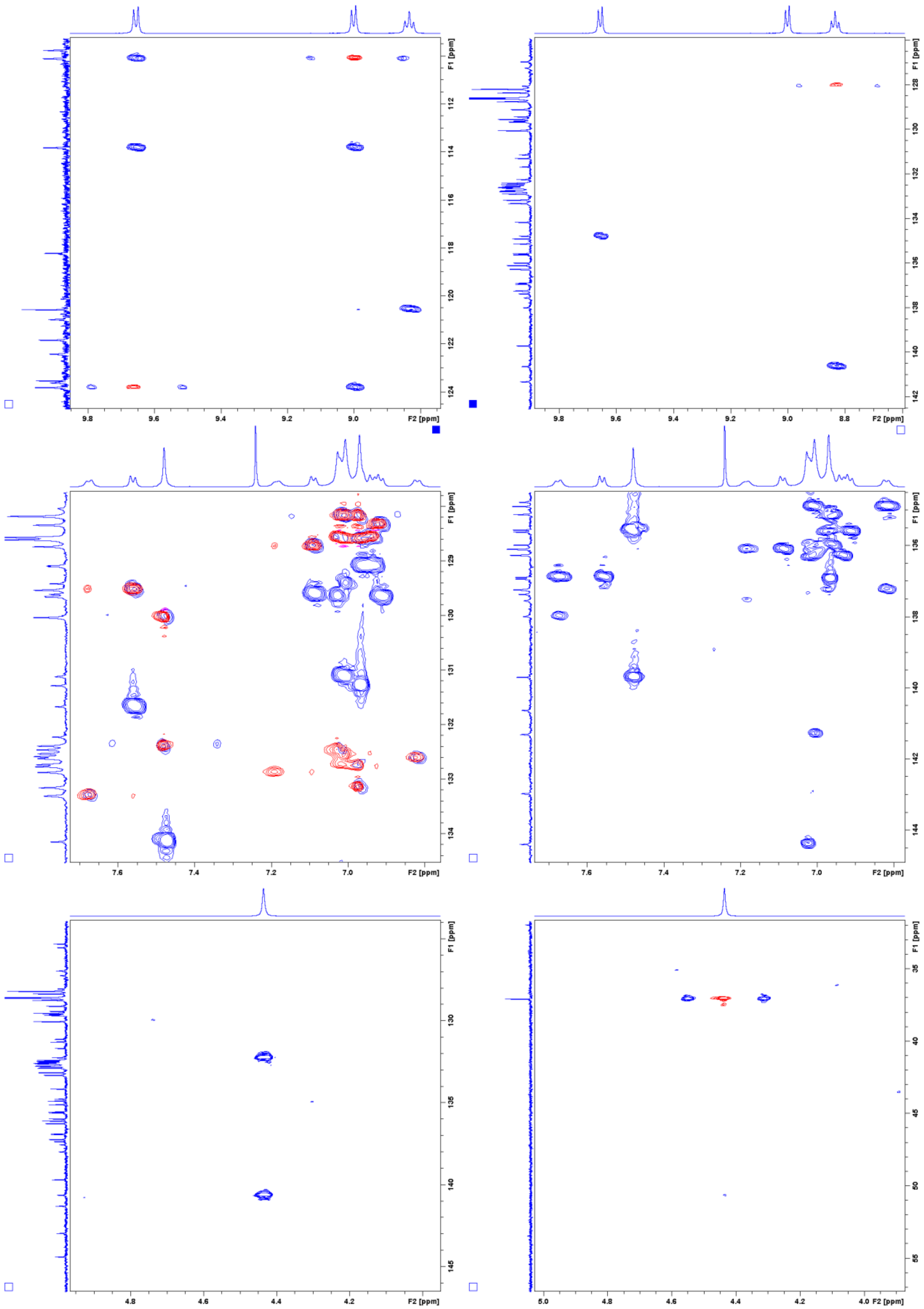

Figure S10. Expansions of ^{1}H HSQC (red) and HMBC (blue) spectra of **5a**$[SbCl_6]_2$ (600 MHz, $CDCl_3$, 300 K).

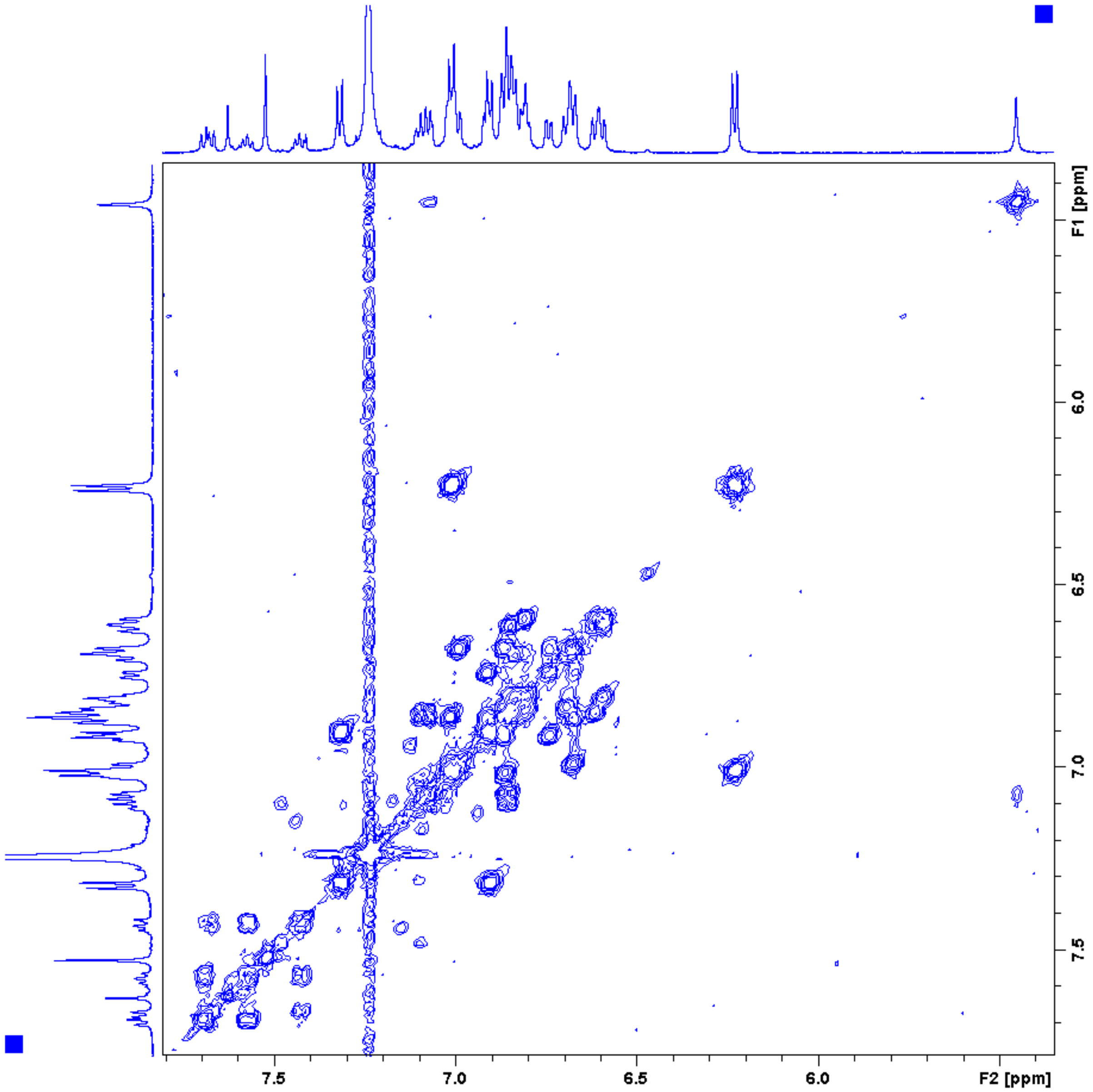

Figure S11. Partial ^{1}H COSY spectrum of **9b**$[SbCl_6]_2$ (600 MHz, $CDCl_3$, 260 K).

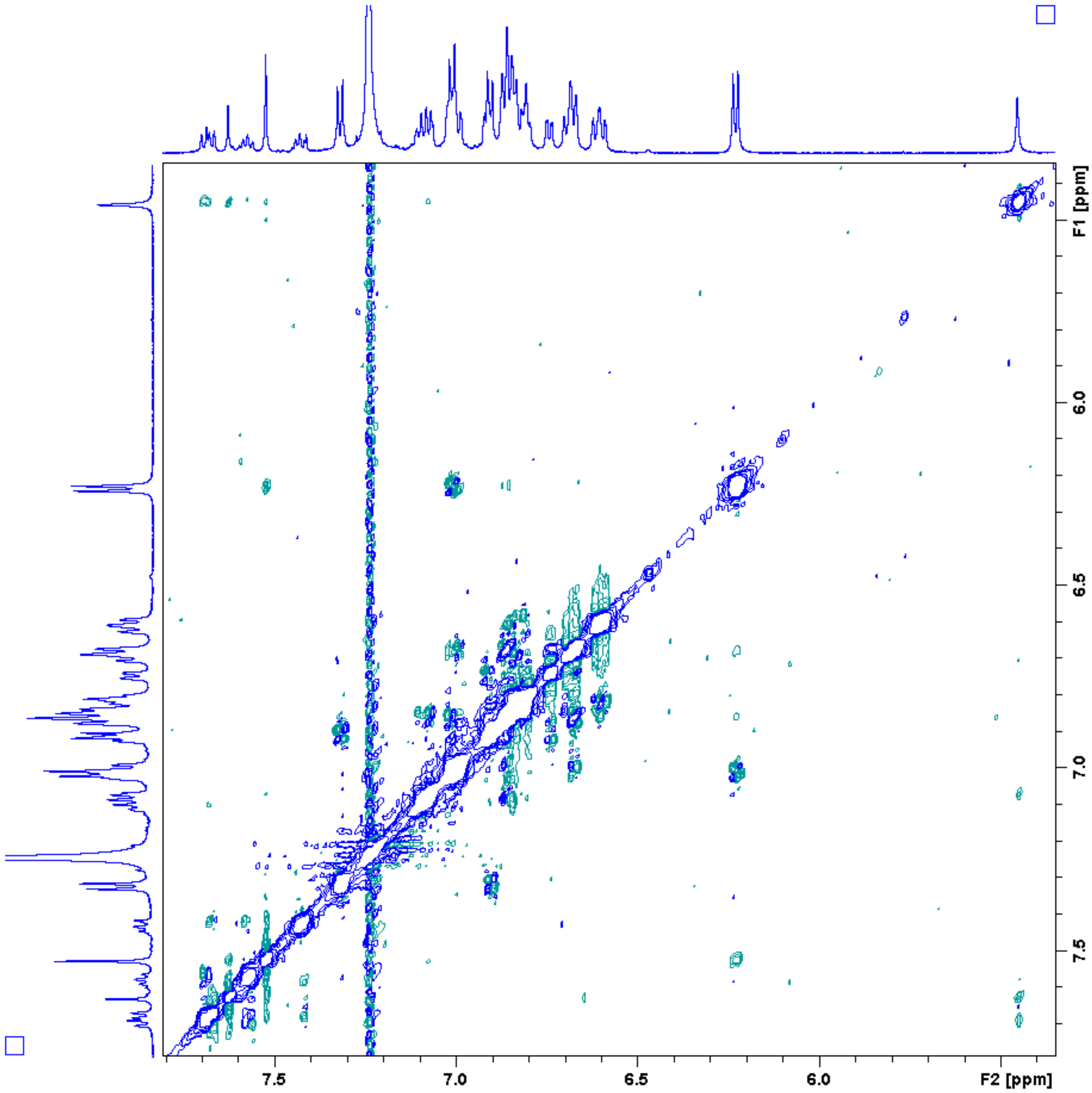

Figure S12. Partial ^{1}H ROESY spectrum of **9b**$[SbCl_6]_2$ (600 MHz, $CDCl_3$, 260 K).

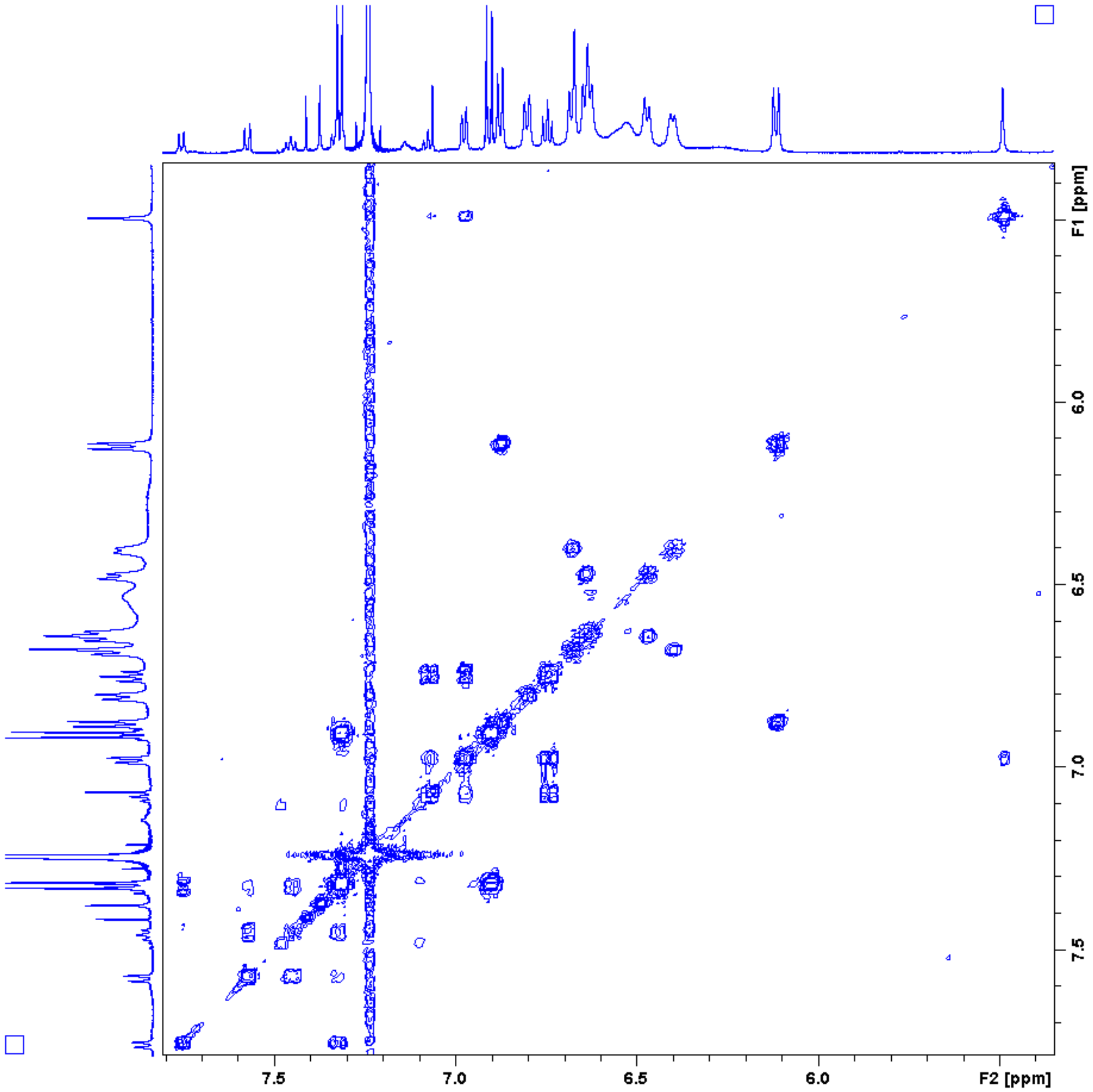

Figure S13. Partial ^{1}H COSY spectrum of **9b** (600 MHz, $CDCl_3$, 260 K).

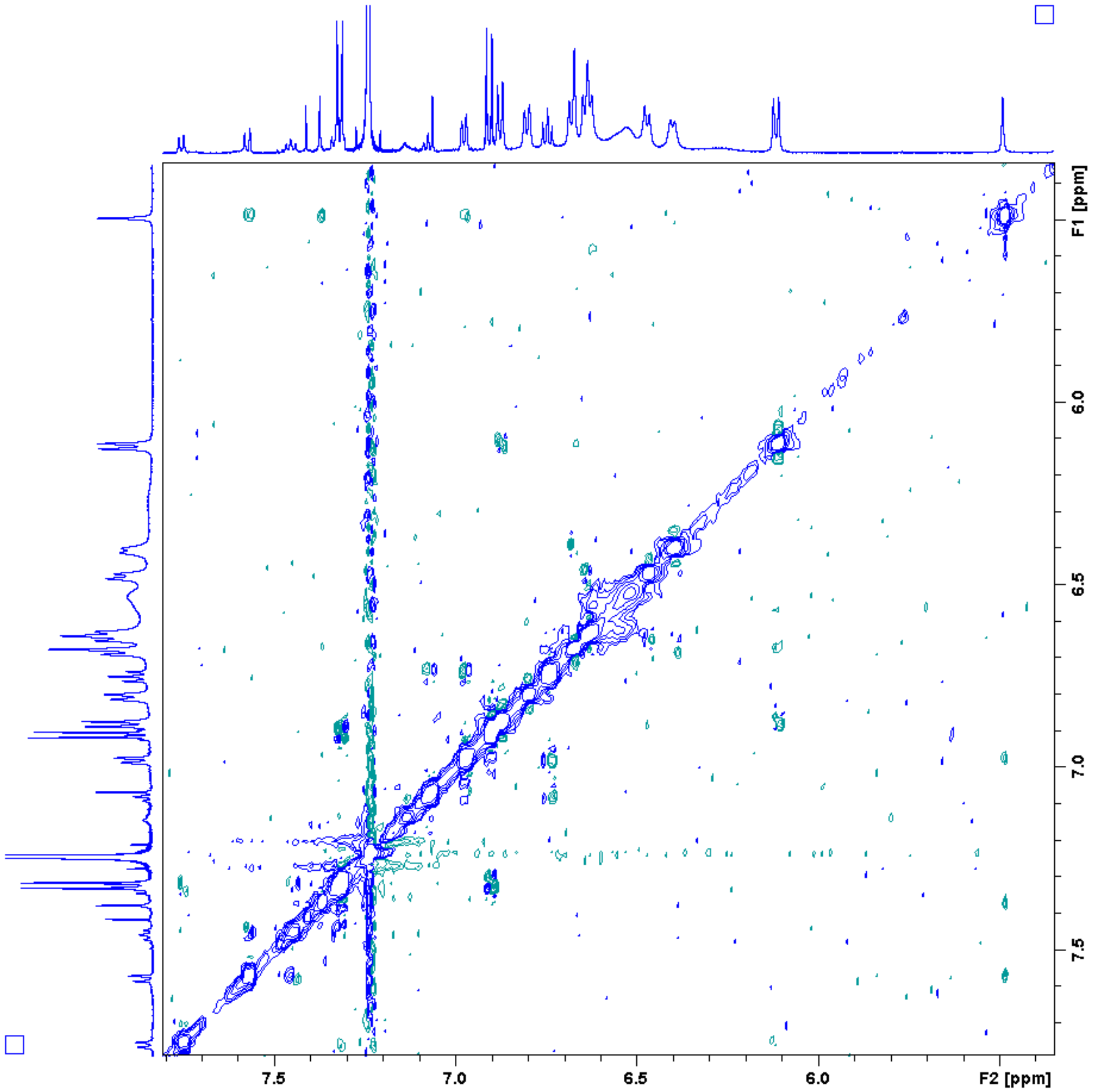

Figure S14. Partial ^1H ROESY spectrum of **9b** (600 MHz, $CDCl_3$, 260 K).

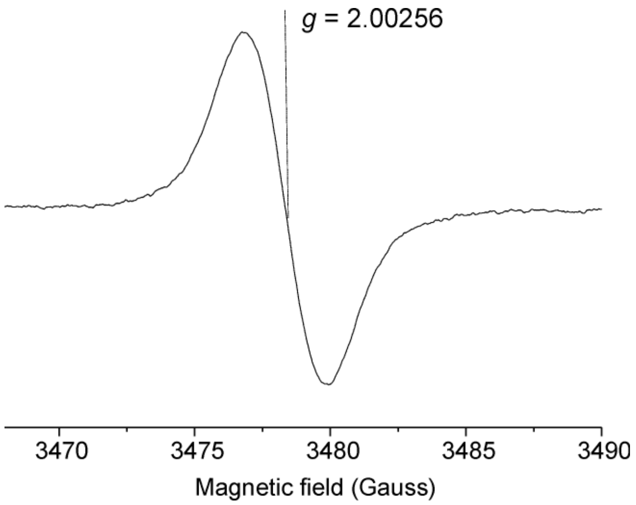

Figure S15. ESR spectrum of a DCM solution of **5a**$[SbCl_6]_2$ recorded at room temperature.

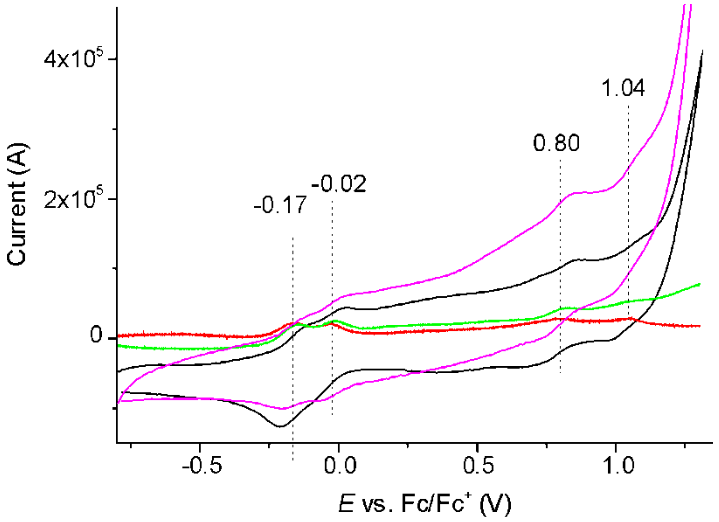

Figure S16. Voltammograms of **5a**$^{2+}$ obtained by chemical oxidation of **8a** with BAHA (cyclic voltammetry, black traces; square wave voltammetry, red trace) and by potential-controlled electrolysis at 1.4 V carried out for 20 minutes (cyclic voltammetry, purple traces; square wave voltammetry, green trace). Conditions: solvent, DCM; supporting electrolyte, $[Bu_4N]ClO_4$; scan rate, 100 mV/s; working electrode, glassy carbon; reference electrode, Ag/AgCl; auxiliary electrode, Pt rod. Potentials were referenced against ferrocene as an internal standard.

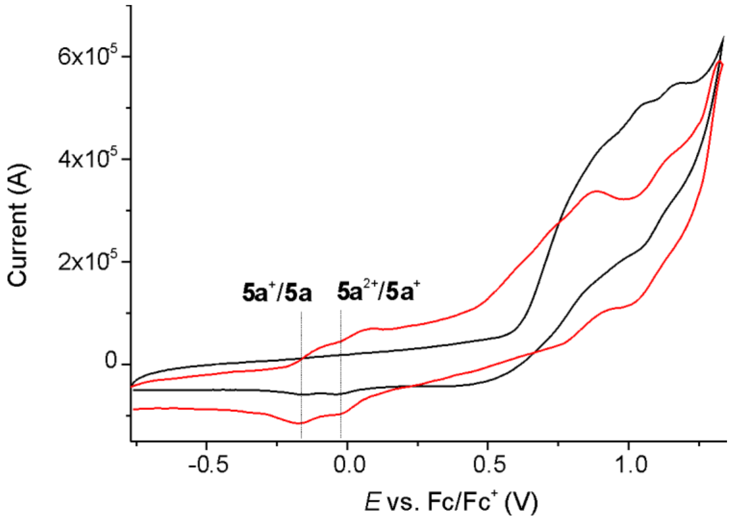

Figure S17. Cyclic voltammogram of **8a** (black traces) and **5a**$^{2+}$ obtained by a potential-controlled electrolysis at 1.4 V carried out for 30 minutes (red traces). Note the presence of reduction waves **5a**$^{2+}$/**5a**$^{+}$ and **5a**$^{+}$/**5a** in the cathodic scan of voltammogram of **8a**, indicative of the formation of **5a**$^{2+}$ in the double layer of the working electrode. Conditions: solvent, DCM; supporting electrolyte, $[Bu_4N]ClO_4$; scan rate, 100 mV/s; working electrode, glassy carbon; reference electrode, Ag/AgCl; auxiliary electrode, Pt rod. Potentials were referenced against ferrocene as an internal standard.

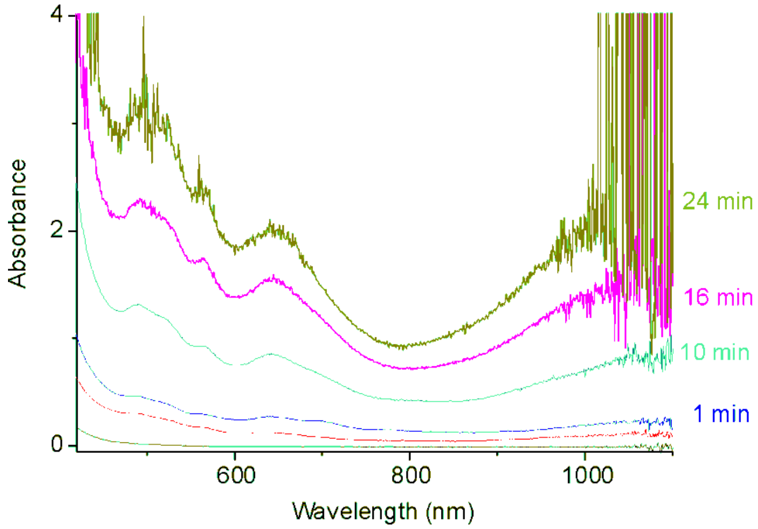

Figure S18. Spectral changes observed upon electroconversion of **8a** into **5a**$^{2+}$ in dichloromethane solution of $[Bu_4N]ClO_4$ (0.08 M). Black trace represents long-wavelength range of the optical spectrum of **8a**, red trace is the spectrum of the same solution after one scan of an *in situ* cyclic voltammetry in the –0.3-1.4 V range, the other spectra were recorded after 1, 10, 16, and 24 minutes of *in situ* electrolysis conducted at the potential of 0.8 V (vs. ferrocene). Conditions: optical path, 1 cm; working electrode, Pt gauze; reference electrode Ag/Ag$^+$; auxiliary electrode, Pt wire.

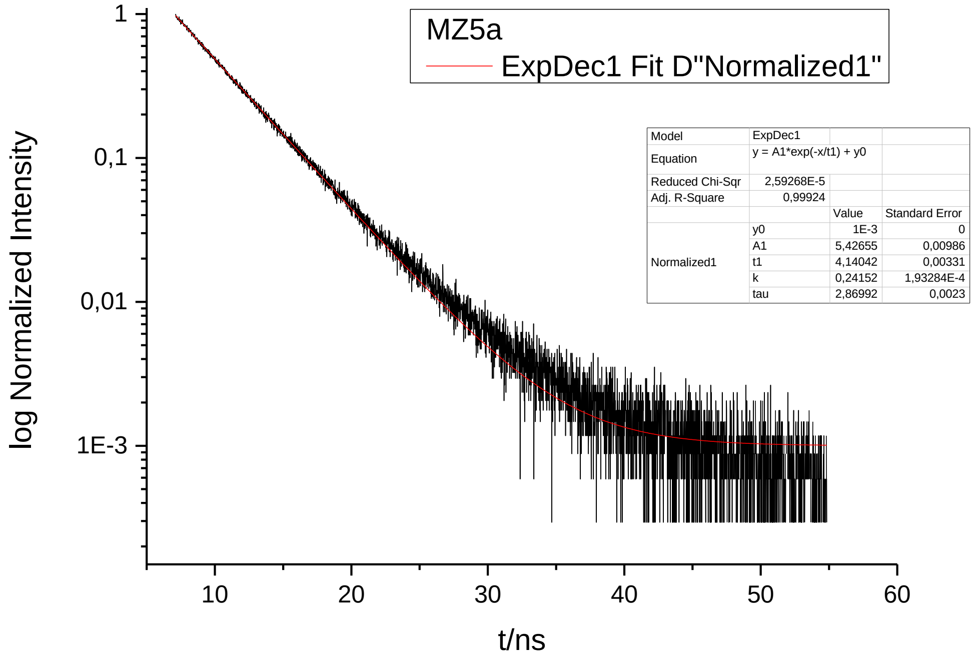

Figure S19. Fluorescence decay time measured for **5a** (dichloromethane, RT).

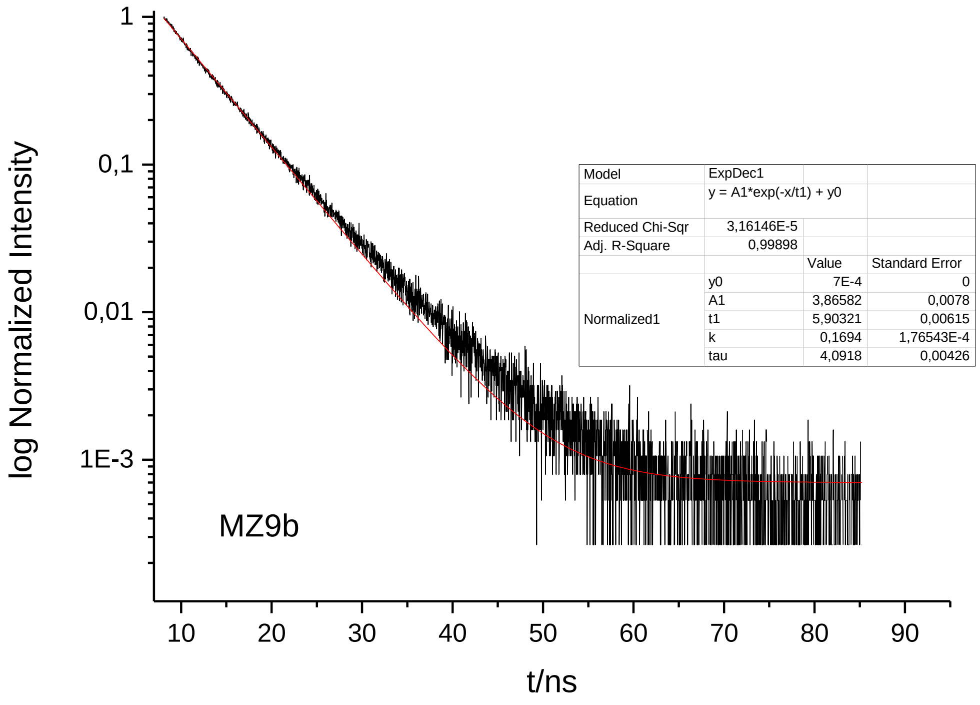

Figure S20. Fluorescence decay time measured for **9b** (dichloromethane, RT).

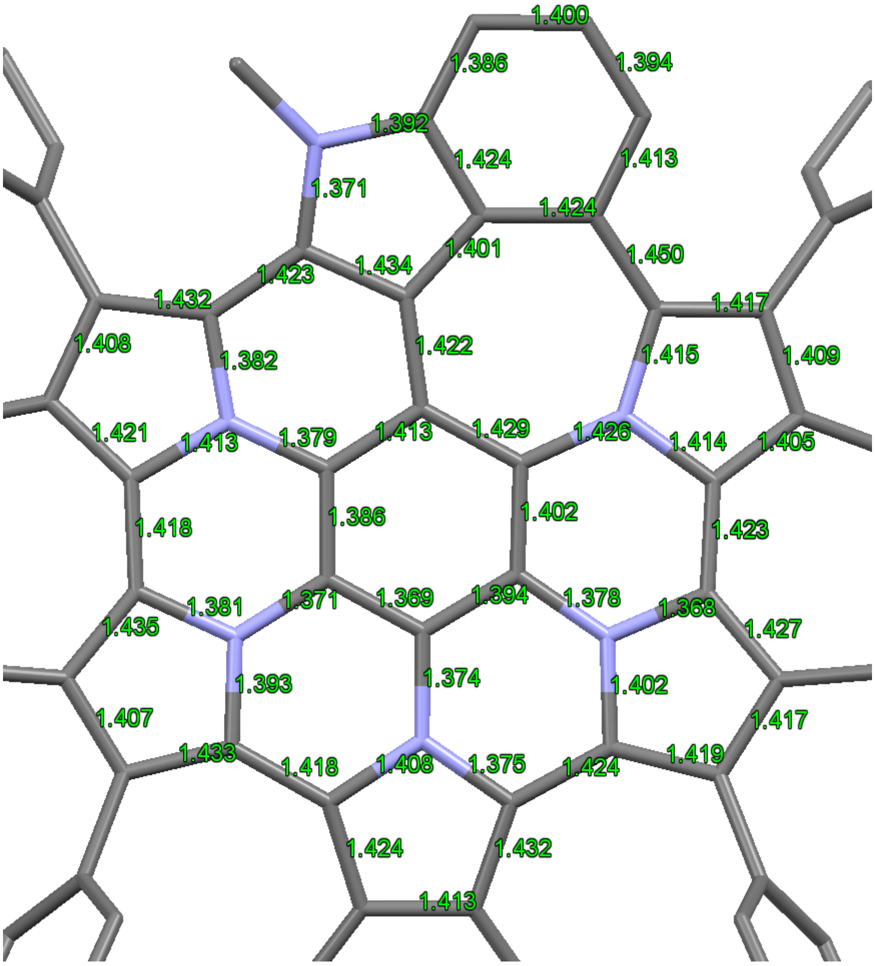

Figure S21. Selected bond lengths in the optimized geometry of **5a**$^{2+}$ (PCM($CHCl_3$)/B3LYP/6-31G(d,p)).

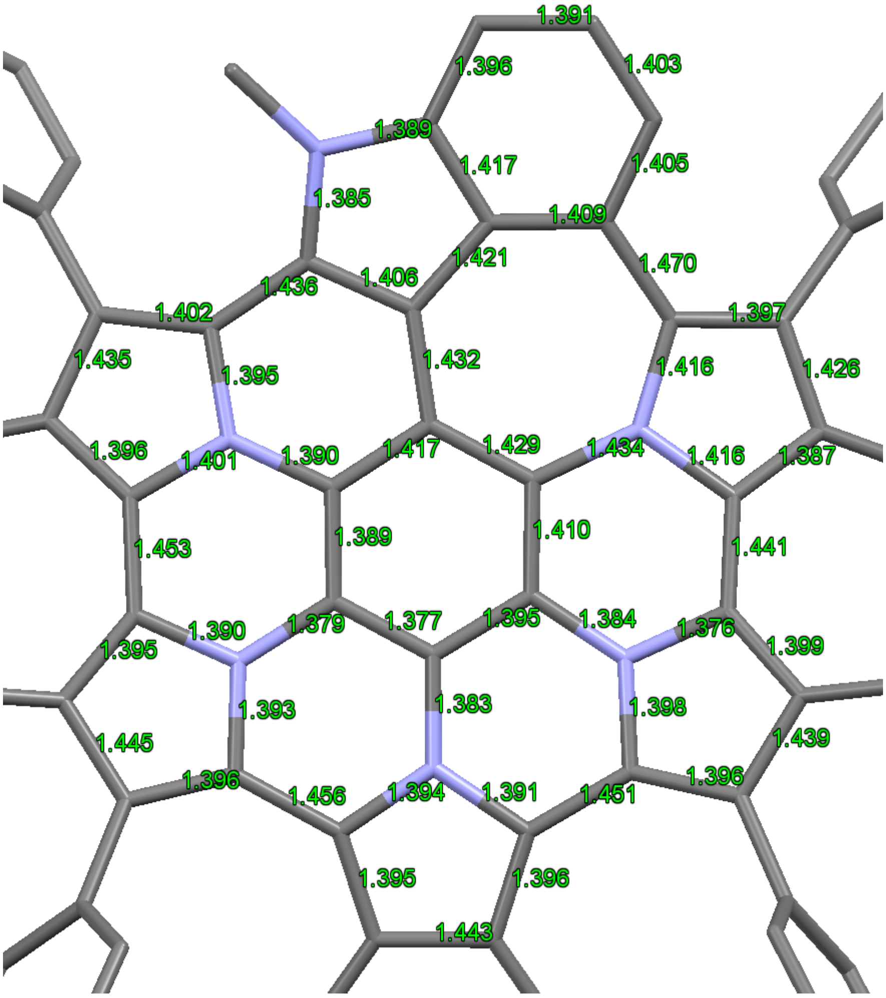

Figure S22. Selected bond lengths in the optimized geometry of **5a** (PCM($CHCl_3$)/B3LYP/6-31G(d,p)).

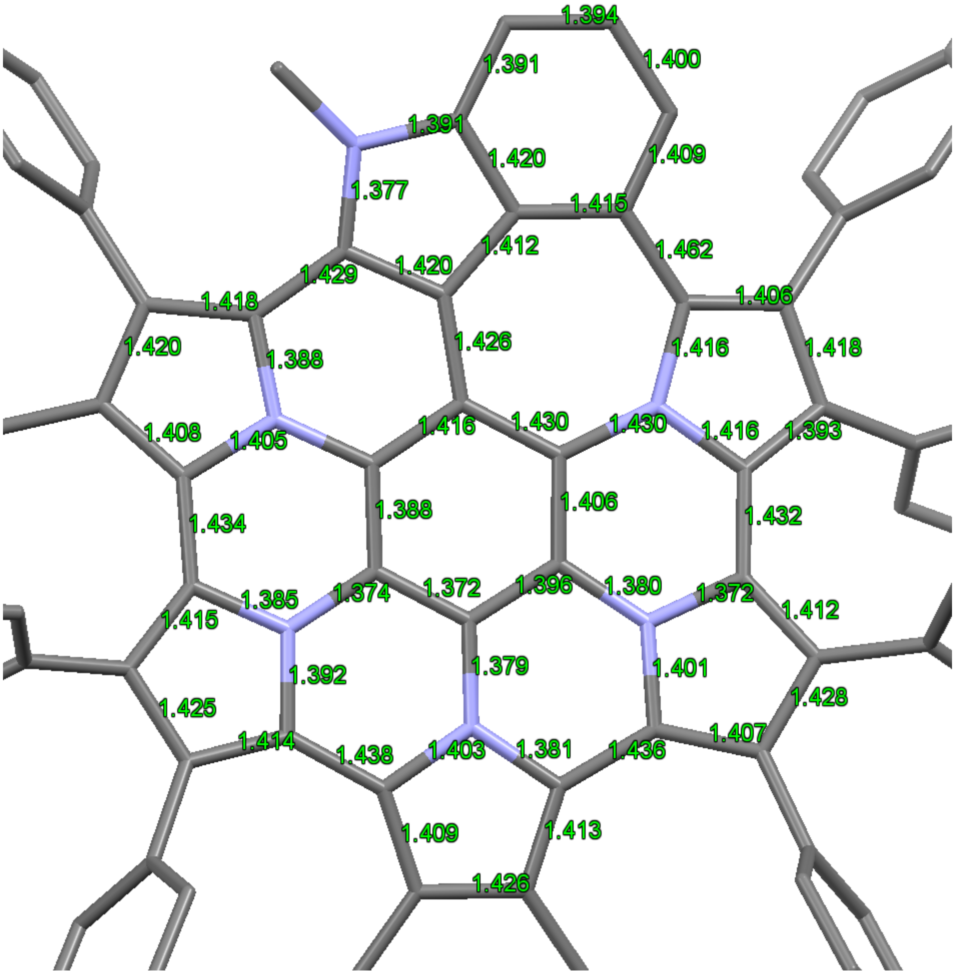

Figure S23. Selected bond lengths in the optimized geometry of **5a**$^+$ (PCM($CHCl_3$)/B3LYP/6-31G(d,p)).

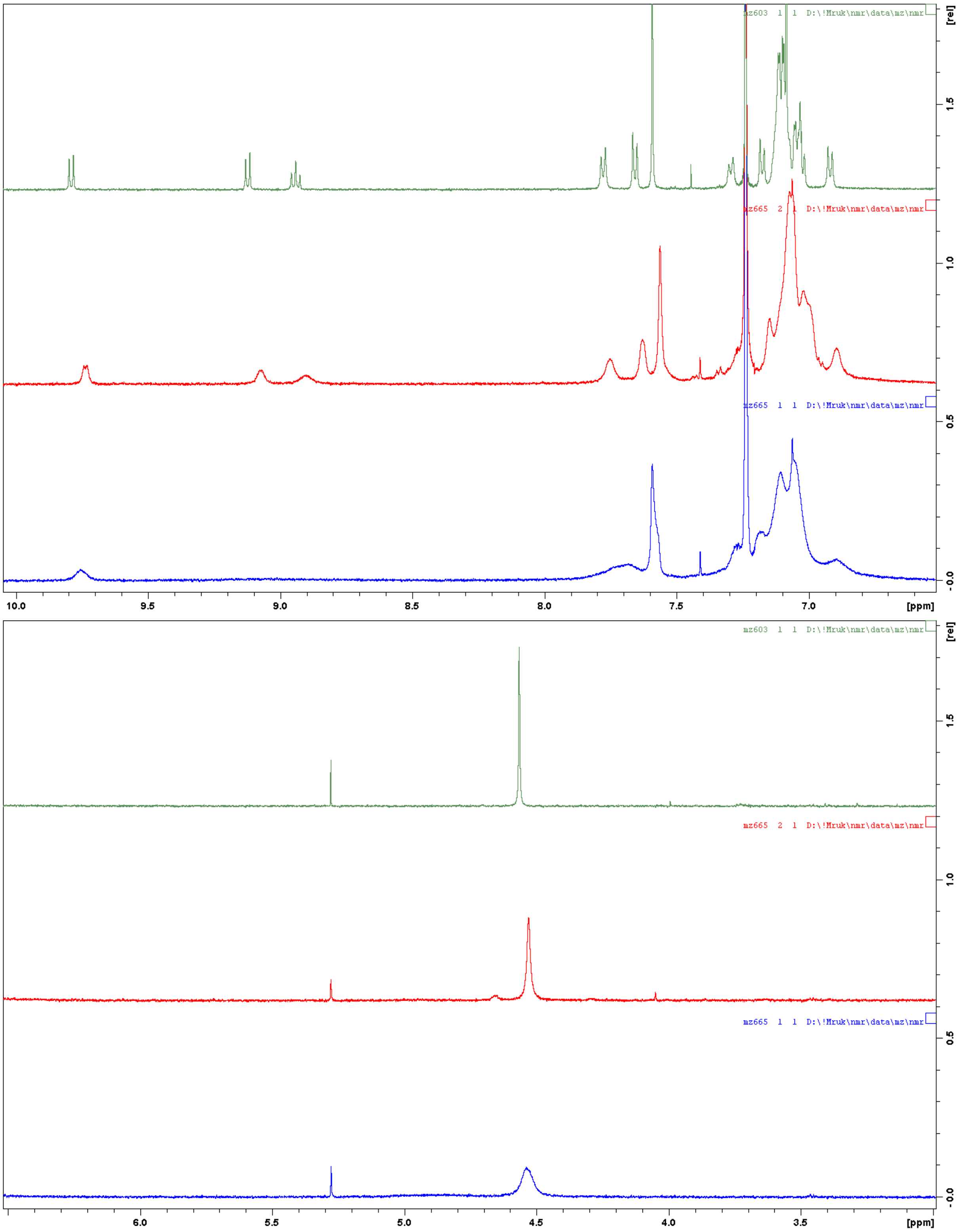

Figure S24. Changes of the ^{1}H NMR spectrum of **5a**$^{2+}$, containing increasing amounts of the **5a**$^{+}$ radical (from top to bottom, $CDCl_3$, 500 MHz, 300 K).

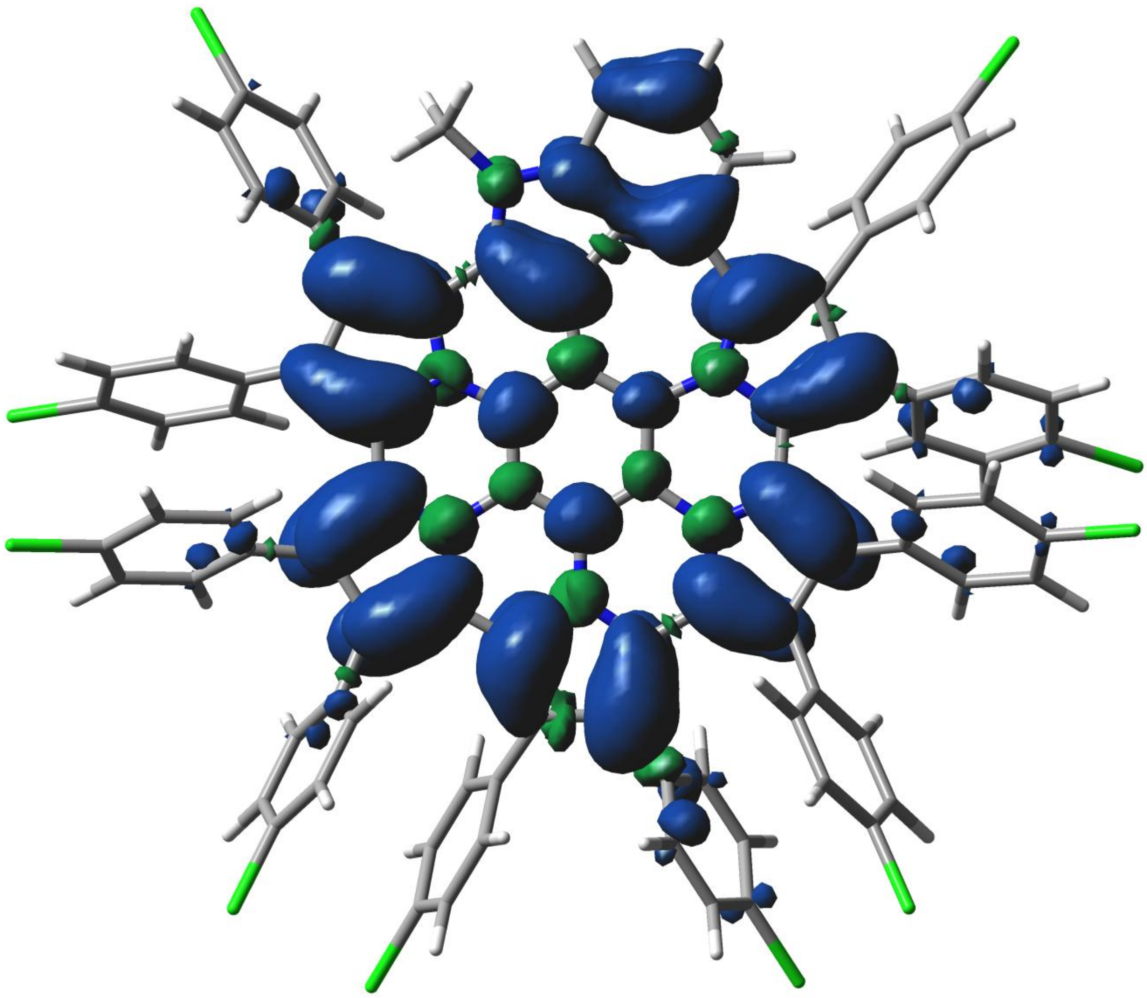

Figure S25. Total spin density calculated for **5a**$^+$ at the PCM(CH_2Cl_2)/B3LYP/6-31G(d,p) level of theory.

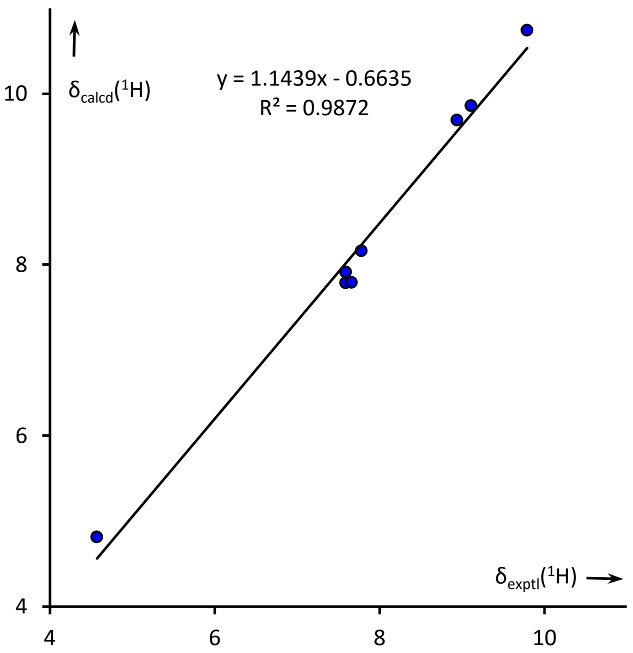

Figure S26. Correlation between ^{1}H NMR chemical shifts of **5a**$^{2+}$ derived from experiment (δ_{exptl}, $SbCl_6$ salt, 500 MHz, chloroform-*d*, 300 K) and theory (δ_{calcd}, PCM($CHCl_3$)/GIAO-B3LYP/6-31G(d,p)). Signals in the experimental spectrum were assigned on the basis of 2D NMR data. Only signals of the indole unit, 9-Ar, and 26-Ar substituents are included in the plot.

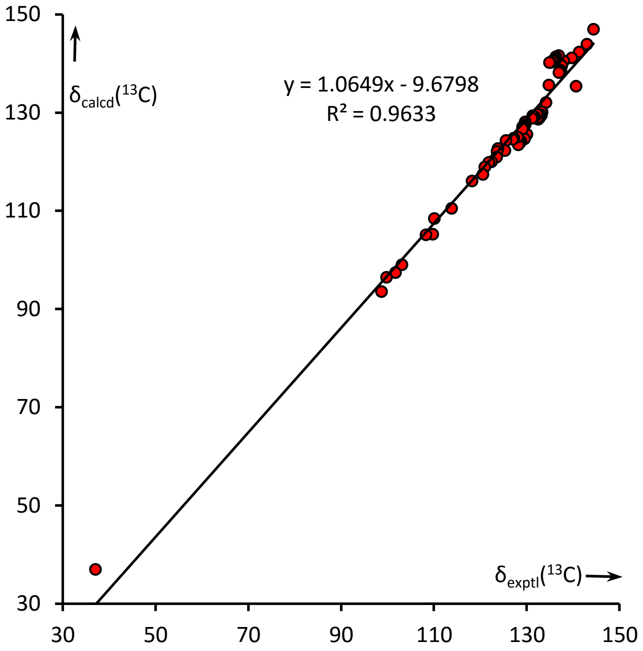

Figure S27. Correlation between ^{13}C NMR chemical shifts of **5a**$^{2+}$ derived from experiment (δ_{exptl}, $SbCl_6$ salt, 150 MHz, chloroform-*d*, 300 K) and theory (δ_{calcd}, PCM($CHCl_3$)/GIAO-B3LYP/6-31G(d,p)). Signals in the experimental spectrum were assigned into chemically distinct groups on the basis of 2D NMR data. Within each group (e.g. *o*-Ar, *m*-Ar), the assignment was arbitrary (differences in chemical shifts were assumed to be smaller than the accuracy of the method).

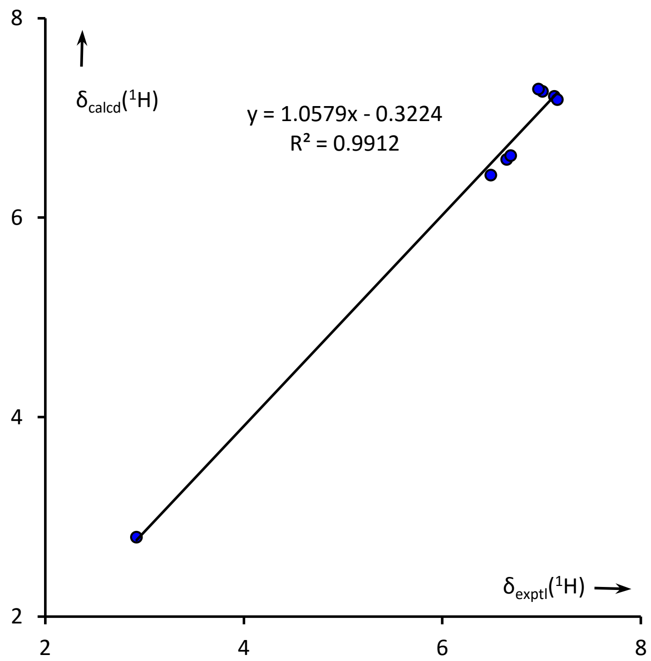

Figure S28. Correlation between ^{1}H NMR chemical shifts of **5a** derived from experiment (δ_{exptl}, 500 MHz, chloroform-*d*, 300 K) and theory (δ_{calcd}, PCM($CHCl_3$)/GIAO-B3LYP/6-31G(d,p)). Signals in the experimental spectrum were assigned on the basis of 2D NMR data. Only signals of the indole unit, 9-Ar, and 26-Ar substituents are included in the plot.

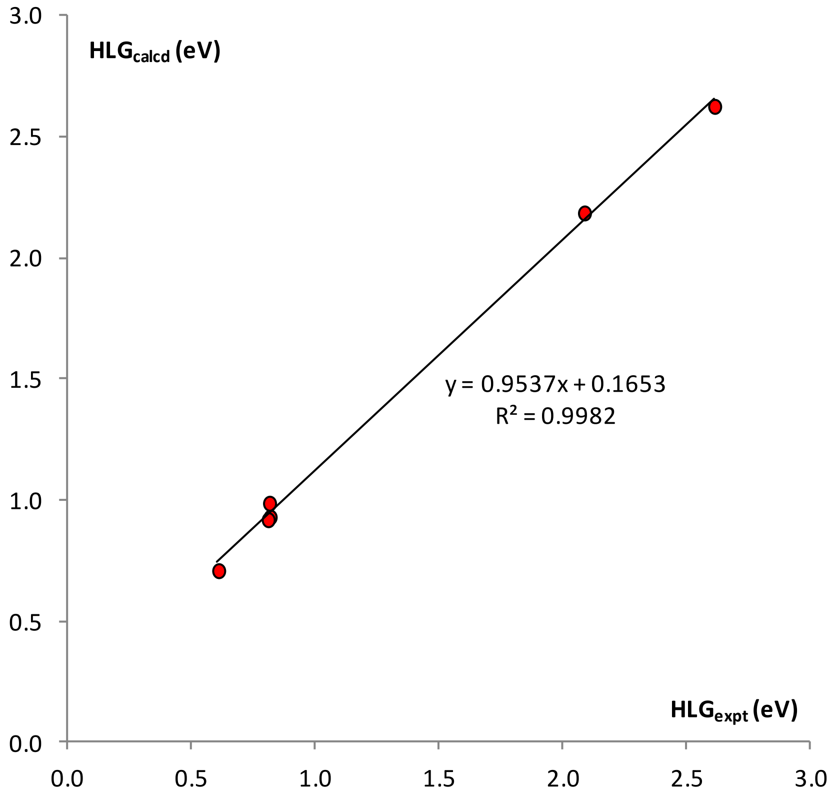

Figure S29. Correlation between experimental and calculated optical HOMO–LUMO gaps for differently oxidized forms **5a**$^{n+}$ and **9b**$^{n+}$ (n = 0, 1, 2).

Table S1. Geometry optimization data at the PCM($CHCl_3$)/B3LYP/6-31G(d,p) level of theory.

Name[a]	Stoichiometry	SCF Energy (a.u.)	ZPV[b] (a.u.)	lowest freq.[c] (cm^{-1})
**1_0**	$C_{30}H_{12}N_6$	−1478.9706145	0.349952	74.99
**1_1+**	$C_{30}H_{12}N_6^{(1+,2)}$	−1478.8061709	0.350104	63.49
**1_2+**	$C_{30}H_{12}N_6^{(2+)}$	−1478.6037587	0.351904	76.88
**5a_0**	$C_{95}H_{46}Cl_{10}N_6$	−8578.4874058	1.134450	7.61
**5a_1+**	$C_{95}H_{46}Cl_{10}N_6^{(1+,2)}$	−8578.3175813	1.134923	9.60
**5a_2+**	$C_{95}H_{46}Cl_{10}N_6^{(2+)}$	−8578.1153147	1.136652	10.29
**5b_0**	$C_{101}H_{50}Cl_{10}N_6$	−8809.5429461	1.215660	7.28
**5b_1+**	$C_{101}H_{50}Cl_{10}N_6^{(1+,2)}$	−8809.3727816	1.216073	8.81
**5b_2+**	$C_{101}H_{50}Cl_{10}N_6^{(2+)}$	−8809.1703262	1.217851	9.62
**5_0**	$C_{34}H_{14}N_6$	−1632.6604588	0.397410	56.09
**5_1+**	$C_{34}H_{14}N_6^{(1+,2)}$	−1632.5005459	0.398365	57.09
**5_2+**	$C_{34}H_{14}N_6^{(2+)}$	−1632.3013584	0.399962	47.21
**9b_0**	$C_{101}H_{54}Cl_{10}N_6$	−8811.9224089	1.258358	8.20
**9b_1+**	$C_{101}H_{54}Cl_{10}N_6^{(1+,2)}$	−8811.7486029	1.259208	8.81
**9b_2+**	$C_{101}H_{54}Cl_{10}N_6^{(2+)}$	−8811.5406504	1.261299	10.95
**9_0**	$C_{34}H_{18}N_6$	−1635.0202284	0.439748	22.91
**9_1+**	$C_{34}H_{18}N_6^{(1+,2)}$	−1634.8591046	0.440991	26.76
**9_2+**	$C_{34}H_{18}N_6^{(2+)}$	−1634.6547707	0.442785	31.73

[a] Cartesian coordinates for optimized geometries available as Name_*.pdb files. [b] Zero-point virbational energy. [c] Lowest vibrational frequency.

Table S2. Electronic transitions calculated for **5a** using the PCM(CH_2Cl_2)/TD-B3LYP/6-31G(d,p) level of theory.

No.	Energy (cm^{-1})	λ (nm)	f[a]	Major excitations[b]
1	17627	567.3	0.009	HOMO»LUMO (98%)
2	20624	484.9	0.002	HOMO»L+1 (96%)
3	22569	443.1	0.040	H–1»LUMO (68%)<br>HOMO»L+2 (25%)
4	22974	435.3	0.085	H–1»LUMO (22%)<br>HOMO»L+2 (66%)
5	24572	407.0	0.026	H–2»LUMO (22%)<br>H–1»L+1 (23%)<br>HOMO»L+3 (41%)
6	25475	392.5	0.496	H–2»LUMO (15%)<br>H–1»L+1 (28%)<br>HOMO»L+3 (45%)
7	25853	386.8	0.129	H–2»LUMO (48%)<br>H–1»L+1 (34%)
8	26620	375.7	0.158	H–1»L+2 (12%)<br>HOMO»L+4 (75%)
9	26978	370.7	0.112	H–2»L+1 (11%)<br>H–1»L+2 (54%)
10	27329	365.9	0.028	HOMO»L+5 (90%)
11	27692	361.1	0.071	HOMO»L+6 (72%)
12	27711	360.9	0.471	H–3»LUMO (40%)<br>H–2»L+1 (20%)<br>HOMO»L+6 (14%)
13	27887	358.6	0.187	H–3»LUMO (20%)<br>H–2»L+1 (44%)<br>H–1»L+2 (15%)
14	27967	357.6	0.070	HOMO»L+7 (54%)<br>HOMO»L+9 (12%)
15	28121	355.6	0.003	HOMO»L+8 (85%)
16	28200	354.6	0.031	HOMO»L+7 (13%)<br>HOMO»L+9 (77%)
17	28456	351.4	0.003	HOMO»L+10 (40%)<br>HOMO»L+11 (31%)<br>HOMO»L+12 (18%)
18	28629	349.3	0.046	HOMO»L+10 (44%)<br>HOMO»L+11 (41%)
19	28829	346.9	0.002	HOMO»L+11 (17%)<br>HOMO»L+12 (66%)
20	29160	342.9	0.009	HOMO»L+13 (73%)
21	29320	341.1	0.006	H–1»L+3 (29%)<br>HOMO»L+14 (48%)
22	29484	339.2	0.043	H–1»L+3 (26%)<br>HOMO»L+13 (11%)<br>HOMO»L+14 (27%)<br>HOMO»L+15 (20%)
23	29775	335.9	0.008	H–2»L+2 (31%)<br>HOMO»L+15 (31%)
24	29859	334.9	0.065	H–2»L+2 (21%)<br>H–1»L+3 (14%)<br>HOMO»L+15 (19%)<br>HOMO»L+16 (23%)
25	29960	333.8	0.034	HOMO»L+15 (14%)<br>HOMO»L+16 (64%)
26	30182	331.3	0.046	H–4»LUMO (10%)<br>HOMO»L+17 (73%)
27	30608	326.7	0.196	H–4»LUMO (23%)<br>H–2»L+2 (18%)<br>H–1»L+4 (28%)
28	30741	325.3	0.545	H–4»LUMO (27%)<br>H–3»L+1 (18%)<br>HOMO»L+19 (10%)
29	31145	321.1	0.047	H–3»L+1 (11%)<br>H–1»L+4 (42%)<br>HOMO»L+20 (18%)
30	31148	321.1	0.046	HOMO»L+18 (86%)
31	31405	318.4	0.081	HOMO»L+19 (54%)<br>HOMO»L+20 (17%)
32	31778	314.7	0.662	H–3»L+1 (11%)<br>HOMO»L+19 (19%)<br>HOMO»L+20 (42%)
33	31894	313.5	0.052	H–2»L+3 (70%)
34	32012	312.4	0.226	HOMO»L+21 (79%)
35	32297	309.6	0.017	H–1»L+5 (65%)<br>HOMO»L+24 (12%)
36	32318	309.4	0.070	H–3»L+2 (21%)<br>H–1»L+5 (20%)<br>HOMO»L+24 (32%)
37	32386	308.8	0.012	HOMO»L+22 (29%)<br>HOMO»L+23 (50%)
38	32450	308.2	0.126	H–5»LUMO (57%)<br>HOMO»L+22 (15%)
39	32538	307.3	0.184	H–1»L+7 (27%)<br>HOMO»L+22 (25%)<br>HOMO»L+23 (13%)
40	32659	306.2	0.196	H–1»L+6 (44%)<br>HOMO»L+23 (18%)
41	32816	304.7	0.133	H–4»L+1 (12%)<br>H–3»L+2 (23%)<br>H–1»L+6 (18%)<br>HOMO»L+24 (30%)
42	32864	304.3	0.080	H–1»L+6 (19%)<br>H–1»L+7 (44%)
43	33127	301.9	0.052	H–1»L+8 (64%)<br>H–1»L+11 (15%)
44	33196	301.2	0.051	H–1»L+9 (69%)
45	33319	300.1	0.001	H–1»L+8 (15%)<br>H–1»L+11 (55%)
46	33369	299.7	0.008	H–1»L+10 (29%)<br>H–1»L+12 (57%)
47	33442	299.0	0.301	H–4»L+1 (40%)<br>H–1»L+10 (18%)
48	33536	298.2	0.069	H–1»L+10 (36%)<br>H–1»L+11 (17%)<br>H–1»L+12 (13%)
49	33621	297.4	0.054	H–6»LUMO (72%)
50	33808	295.8	0.047	H–2»L+4 (53%)

[a] Oscillator strength. [b] Contributions smaller than 10% are not included. H = HOMO, L = LUMO. Orbitals are numbered consecutively regardless of possible degeneracies.

Table S3. Electronic transitions calculated for **5a**$^{+}$ using the PCM(CH_2Cl_2)/TD-B3LYP/6-31G(d,p) level of theory.

No.	Energy (cm^{-1})	λ (nm)	*f*[a]	Major excitations[b]
1	5720	1748.2	0.106	HOMO(B)»LUMO(B) (96%)
2	8183	1222.1	0.185	H–1(B)»LUMO(B) (95%)
3	10809	925.2	0.020	H–2(B)»LUMO(B) (95%)
4	13333	750.0	0.142	H–3(B)»LUMO(B) (90%)
5	15066	663.8	0.006	HOMO(A)»LUMO(A) (89%)
6	15418	648.6	0.030	H–5(B)»LUMO(B) (28%)<br>H–4(B)»LUMO(B) (55%)
7	15934	627.6	0.020	H–6(B)»LUMO(B) (23%)<br>H–5(B)»LUMO(B) (32%)<br>H–4(B)»LUMO(B) (38%)
8	16249	615.4	0.004	H–6(B)»LUMO(B) (65%)<br>H–5(B)»LUMO(B) (33%)
9	16811	594.8	0.019	H–7(B)»LUMO(B) (87%)
10	17134	583.6	0.011	H–8(B)»LUMO(B) (46%)<br>HOMO(B)»L+1(B) (19%)
11	17529	570.5	0.015	H–1(A)»LUMO(A) (16%)<br>H–8(B)»LUMO(B) (38%)<br>HOMO(B)»L+1(B) (32%)
12	17853	560.1	0.002	H–9(B)»LUMO(B) (88%)
13	18349	545.0	0.005	H–11(B)»LUMO(B) (86%)
14	18416	543.0	0.008	H–10(B)»LUMO(B) (34%)<br>H–1(B)»L+1(B) (14%)
15	18593	537.8	0.004	HOMO(A)»L+1(A) (12%)<br>H–10(B)»LUMO(B) (44%)<br>H–1(B)»L+1(B) (14%)
16	18874	529.8	0.005	H–12(B)»LUMO(B) (96%)
17	19326	517.4	0.002	HOMO(A)»L+1(A) (19%)<br>H–13(B)»LUMO(B) (71%)
18	19503	512.7	0.008	HOMO(A)»L+1(A) (38%)<br>H–13(B)»LUMO(B) (20%)
19	19936	501.6	0.003	H–14(B)»LUMO(B) (80%)
20	20580	485.9	0.003	H–21(B)»LUMO(B) (13%)<br>H–15(B)»LUMO(B) (63%)

[a] Oscillator strength. [b] Contributions smaller than 10% are not included. H = HOMO, L = LUMO. Orbitals are numbered consecutively regardless of possible degeneracies.

Table S4. Electronic transitions calculated for **5a**$^{2+}$ using the PCM(CH_2Cl_2)/TD-B3LYP/6-31G(d,p) level of theory.

No.	Energy (cm^{-1})	λ (nm)	f[a]	Major excitations[b]
1	7507	1332.2	0.156	HOMO»LUMO (89%)
2	9780	1022.5	0.335	H–1»LUMO (82%)
3	11193	893.4	0.046	H–2»LUMO (89%)
4	13183	758.5	0.107	H–4»LUMO (14%)<br>H–3»LUMO (83%)
5	13599	735.3	0.012	H–5»LUMO (91%)
6	13626	733.9	0.037	H–4»LUMO (78%)<br>H–3»LUMO (12%)
7	14779	676.6	0.029	H–7»LUMO (24%)<br>H–6»LUMO (64%)
8	15022	665.7	0.072	H–7»LUMO (61%)<br>H–6»LUMO (32%)
9	15391	649.7	0.136	H–8»LUMO (82%)
10	15875	629.9	0.027	H–9»LUMO (83%)<br>H–8»LUMO (11%)
11	16029	623.9	0.022	H–12»LUMO (13%)<br>H–11»LUMO (12%)<br>H–10»LUMO (68%)
12	16134	619.8	0.010	H–11»LUMO (82%)<br>H–10»LUMO (11%)
13	16247	615.5	0.005	H–12»LUMO (82%)<br>H–10»LUMO (14%)
14	17441	573.4	0.037	H–13»LUMO (92%)
15	17884	559.2	0.035	H–14»LUMO (86%)
16	18212	549.1	0.005	H–16»LUMO (71%)<br>H–15»LUMO (23%)
17	18235	548.4	0.012	H–16»LUMO (27%)<br>H–15»LUMO (62%)
18	18745	533.5	0.005	H–18»LUMO (59%)<br>H–17»LUMO (25%)
19	19035	525.4	0.000	H–18»LUMO (27%)<br>H–17»LUMO (66%)
20	19395	515.6	0.000	H–19»LUMO (93%)
21	19990	500.3	0.017	H–21»LUMO (11%)<br>H–20»LUMO (78%)
22	20145	496.4	0.103	H–21»LUMO (30%)<br>H–20»LUMO (15%)<br>HOMO»L+1 (37%)
23	20273	493.3	0.106	H–21»LUMO (57%)<br>HOMO»L+1 (28%)
24	20843	479.8	0.001	H–22»LUMO (95%)
25	21032	475.5	0.016	H–25»LUMO (12%)<br>H–24»LUMO (65%)<br>H–23»LUMO (11%)
26	21234	470.9	0.002	H–23»LUMO (87%)
27	21611	462.7	0.224	H–1»L+1 (60%)<br>HOMO»L+1 (10%)<br>HOMO»L+2 (13%)

No.	Energy (cm^{-1})	λ (nm)	f[a]	Major excitations[b]
28	22265	449.1	0.015	H–25»LUMO (75%)<br>H–24»LUMO (14%)
29	24144	414.2	0.121	H–2»L+1 (23%)<br>HOMO»L+2 (59%)
30	24364	410.4	0.002	H–26»LUMO (88%)
31	25163	397.4	0.279	H–2»L+1 (42%)<br>H–1»L+2 (36%)
32	25430	393.2	0.685	H–2»L+1 (27%)<br>H–1»L+2 (49%)
33	26053	383.8	0.036	HOMO»L+3 (75%)
34	26563	376.5	0.012	H–27»LUMO (91%)
35	26759	373.7	0.365	H–3»L+1 (87%)
36	27216	367.4	0.006	H–4»L+1 (92%)
37	27481	363.9	0.013	H–5»L+1 (93%)
38	27538	363.1	0.014	H–28»LUMO (92%)
39	27898	358.4	0.099	H–6»L+1 (23%)<br>H–1»L+3 (57%)
40	28025	356.8	0.118	H–6»L+1 (66%)<br>H–1»L+3 (21%)
41	28505	350.8	0.052	H–7»L+1 (79%)
42	28836	346.8	0.047	H–8»L+1 (74%)<br>HOMO»L+4 (12%)
43	29053	344.2	0.094	H–39»LUMO (11%)<br>H–8»L+1 (17%)<br>HOMO»L+4 (38%)
44	29118	343.4	0.030	H–39»LUMO (19%)<br>H–29»LUMO (14%)<br>H–2»L+2 (13%)<br>HOMO»L+4 (13%)<br>HOMO»L+5 (12%)
45	29285	341.5	0.036	H–9»L+1 (71%)<br>HOMO»L+5 (12%)
46	29426	339.8	0.063	H–9»L+1 (16%)<br>HOMO»L+5 (57%)
47	29602	337.8	0.008	H–10»L+1 (71%)
48	29896	334.5	0.060	H–12»L+1 (27%)<br>H–11»L+1 (36%)<br>H–2»L+2 (10%)
49	29940	334.0	0.008	H–12»L+1 (33%)<br>H–11»L+1 (52%)
50	29973	333.6	0.001	H–31»LUMO (56%)<br>H–30»LUMO (26%)<br>H–29»LUMO (11%)

[a] Oscillator strength. [b] Contributions smaller than 10% are not included. H = HOMO, L = LUMO. Orbitals are numbered consecutively regardless of possible degeneracies.

Table S5. Electronic transitions calculated for **9b** using the PCM(CH_2Cl_2)/TD-B3LYP/6-31G(d,p) level of theory.

No.	Energy (cm^{-1})	λ (nm)	*f*[a]	Major excitations[b]
1	21172	472.3	0.006	HOMO»LUMO (98%)
2	23570	424.3	0.311	HOMO»L+1 (95%)
3	23720	421.6	0.055	HOMO»L+2 (96%)
4	27227	367.3	0.194	H–1»LUMO (83%)
5	27743	360.4	0.257	HOMO»L+3 (78%)
6	28363	352.6	0.056	H–2»LUMO (27%)<br>H–1»L+2 (44%)<br>HOMO»L+4 (22%)
7	28610	349.5	0.019	HOMO»L+5 (27%)<br>HOMO»L+6 (62%)
8	28710	348.3	0.005	HOMO»L+4 (20%)<br>HOMO»L+7 (64%)
9	28828	346.9	0.227	H–2»LUMO (20%)<br>HOMO»L+4 (37%)<br>HOMO»L+7 (23%)<br>HOMO»L+8 (12%)
10	29041	344.3	0.007	HOMO»L+5 (53%)<br>HOMO»L+6 (25%)
11	29242	342.0	0.017	H–1»L+1 (68%)<br>HOMO»L+5 (12%)
12	29326	341.0	0.349	H–2»LUMO (19%)<br>H–1»L+2 (25%)<br>HOMO»L+8 (33%)<br>HOMO»L+10 (11%)
13	29529	338.7	0.412	H–2»LUMO (13%)<br>H–1»L+2 (21%)<br>HOMO»L+4 (10%)<br>HOMO»L+8 (20%)<br>HOMO»L+10 (23%)
14	29598	337.9	0.003	HOMO»L+11 (62%)
15	29713	336.6	0.026	HOMO»L+8 (25%)<br>HOMO»L+10 (50%)
16	29823	335.3	0.011	HOMO»L+9 (68%)<br>HOMO»L+11 (14%)
17	30065	332.6	0.008	HOMO»L+12 (75%)
18	30305	330.0	0.019	HOMO»L+13 (10%)<br>HOMO»L+14 (77%)
19	30437	328.5	0.238	H–3»LUMO (81%)
20	30952	323.1	0.002	HOMO»L+13 (72%)<br>HOMO»L+14 (11%)
21	31049	322.1	0.013	HOMO»L+15 (70%)
22	31195	320.6	0.008	H–2»L+1 (66%)<br>HOMO»L+17 (17%)
23	31379	318.7	0.259	H–4»LUMO (12%)<br>H–2»L+2 (30%)<br>HOMO»L+15 (15%)<br>HOMO»L+16 (26%)
24	31482	317.6	0.072	H–4»LUMO (22%)<br>H–2»L+2 (12%)<br>HOMO»L+16 (48%)
25	31596	316.5	0.027	H–2»L+1 (20%)<br>HOMO»L+17 (52%)<br>HOMO»L+18 (12%)

[a] Oscillator strength. [b] Contributions smaller than 10% are not included. H = HOMO, L = LUMO. Orbitals are numbered consecutively regardless of possible degeneracies.

Table S6. Electronic transitions calculated for **9b**$^+$ using the PCM(CH_2Cl_2)/TD-B3LYP/6-31G(d,p) level of theory.

No.	Energy (cm^{-1})	λ (nm)	*f*[a]	Major excitations[b]
1	7420	1347.8	0.282	HOMO(B)»LUMO(B) (98%)
2	9756	1025.0	0.001	H–2(B)»LUMO(B) (68%)<br>H–1(B)»LUMO(B) (28%)
3	10903	917.2	0.037	H–2(B)»LUMO(B) (28%)<br>H–1(B)»LUMO(B) (72%)
4	12152	822.9	0.064	H–4(B)»LUMO(B) (20%)<br>H–3(B)»LUMO(B) (64%)
5	13965	716.1	0.162	H–4(B)»LUMO(B) (67%)<br>H–3(B)»LUMO(B) (22%)
6	14630	683.5	0.000	H–5(B)»LUMO(B) (86%)
7	15214	657.3	0.044	H–6(B)»LUMO(B) (86%)
8	15642	639.3	0.004	H–9(B)»LUMO(B) (15%)<br>H–8(B)»LUMO(B) (48%)<br>H–7(B)»LUMO(B) (32%)
9	15664	638.4	0.005	H–9(B)»LUMO(B) (15%)<br>H–8(B)»LUMO(B) (20%)<br>H–7(B)»LUMO(B) (61%)
10	15980	625.8	0.003	H–9(B)»LUMO(B) (66%)<br>H–8(B)»LUMO(B) (25%)
11	17089	585.2	0.027	H–11(B)»LUMO(B) (29%)<br>H–10(B)»LUMO(B) (48%)
12	17274	578.9	0.017	H–11(B)»LUMO(B) (46%)<br>H–10(B)»LUMO(B) (38%)
13	17552	569.7	0.003	H–12(B)»LUMO(B) (86%)
14	18322	545.8	0.004	H–15(B)»LUMO(B) (62%)<br>H–14(B)»LUMO(B) (31%)
15	18453	541.9	0.071	HOMO(A)»LUMO(A) (86%)
16	18929	528.3	0.001	H–16(B)»LUMO(B) (72%)<br>H–13(B)»LUMO(B) (14%)
17	19161	521.9	0.000	H–16(B)»LUMO(B) (11%)<br>H–13(B)»LUMO(B) (84%)
18	19512	512.5	0.001	H–15(B)»LUMO(B) (31%)<br>H–14(B)»LUMO(B) (66%)
19	19917	502.1	0.006	H–17(B)»LUMO(B) (79%)
20	20304	492.5	0.000	H–21(B)»LUMO(B) (28%)<br>H–18(B)»LUMO(B) (62%)
21	20462	488.7	0.007	H–1(A)»LUMO(A) (21%)<br>HOMO(B)»L+1(B) (47%)
22	20636	484.6	0.007	H–20(B)»LUMO(B) (18%)<br>H–19(B)»LUMO(B) (59%)
23	20668	483.8	0.036	HOMO(A)»L+1(A) (40%)<br>H–21(B)»LUMO(B) (25%)<br>H–18(B)»LUMO(B) (12%)
24	20966	477.0	0.001	H–23(B)»LUMO(B) (28%)<br>H–20(B)»LUMO(B) (52%)
25	20981	476.6	0.080	HOMO(A)»L+1(A) (38%)<br>H–21(B)»LUMO(B) (38%)

[a] Oscillator strength. [b] Contributions smaller than 10% are not included. H = HOMO, L = LUMO. Orbitals are numbered consecutively regardless of possible degeneracies.

Table S7. Electronic transitions calculated for **9b**$^{2+}$ using the PCM(CH_2Cl_2)/TD-B3LYP/6-31G(d,p) level of theory.

No.	Energy (cm^{-1})	λ (nm)	*f*[a]	Major excitations[b]
1	7970	1254.6	0.018	H–1»LUMO (98%)
2	9470	1056.0	0.468	HOMO»LUMO (93%)
3	10203	980.1	0.031	H–2»LUMO (90%)
4	11570	864.3	0.034	H–3»LUMO (95%)
5	12144	823.4	0.106	H–7»LUMO (13%)<br>H–5»LUMO (72%)
6	12557	796.3	0.034	H–5»LUMO (17%)<br>H–4»LUMO (76%)
7	12979	770.5	0.009	H–8»LUMO (82%)<br>H–6»LUMO (15%)
8	13474	742.1	0.137	H–8»LUMO (12%)<br>H–6»LUMO (72%)
9	13610	734.8	0.108	H–7»LUMO (75%)
10	14814	675.0	0.093	H–12»LUMO (44%)<br>H–11»LUMO (19%)<br>H–9»LUMO (26%)
11	15151	660.0	0.102	H–14»LUMO (14%)<br>H–12»LUMO (25%)<br>H–11»LUMO (45%)
12	15173	659.1	0.001	H–12»LUMO (20%)<br>H–9»LUMO (71%)
13	15333	652.2	0.009	H–13»LUMO (13%)<br>H–11»LUMO (14%)<br>H–10»LUMO (65%)
14	15478	646.1	0.015	H–15»LUMO (53%)<br>H–14»LUMO (14%)<br>H–13»LUMO (14%)<br>H–10»LUMO (17%)
15	15605	640.8	0.000	H–15»LUMO (36%)<br>H–14»LUMO (42%)
16	15670	638.2	0.004	H–14»LUMO (27%)<br>H–13»LUMO (56%)
17	16891	592.0	0.033	H–18»LUMO (27%)<br>H–16»LUMO (64%)
18	17335	576.9	0.029	H–19»LUMO (12%)<br>H–17»LUMO (78%)
19	17471	572.4	0.000	H–21»LUMO (17%)<br>H–18»LUMO (55%)<br>H–16»LUMO (22%)
20	17935	557.6	0.011	H–20»LUMO (83%)<br>H–17»LUMO (10%)
21	18081	553.1	0.000	H–22»LUMO (18%)<br>H–20»LUMO (10%)<br>H–19»LUMO (62%)
22	18349	545.0	0.000	H–23»LUMO (11%)<br>H–21»LUMO (68%)<br>H–18»LUMO (14%)
23	18760	533.1	0.001	H–22»LUMO (75%)<br>H–19»LUMO (17%)
24	18913	528.7	0.001	H–24»LUMO (49%)<br>H–23»LUMO (37%)
25	19236	519.9	0.003	H–24»LUMO (44%)<br>H–23»LUMO (48%)

[a] Oscillator strength. [b] Contributions smaller than 10% are not included. H = HOMO, L = LUMO. Orbitals are numbered consecutively regardless of possible degeneracies.

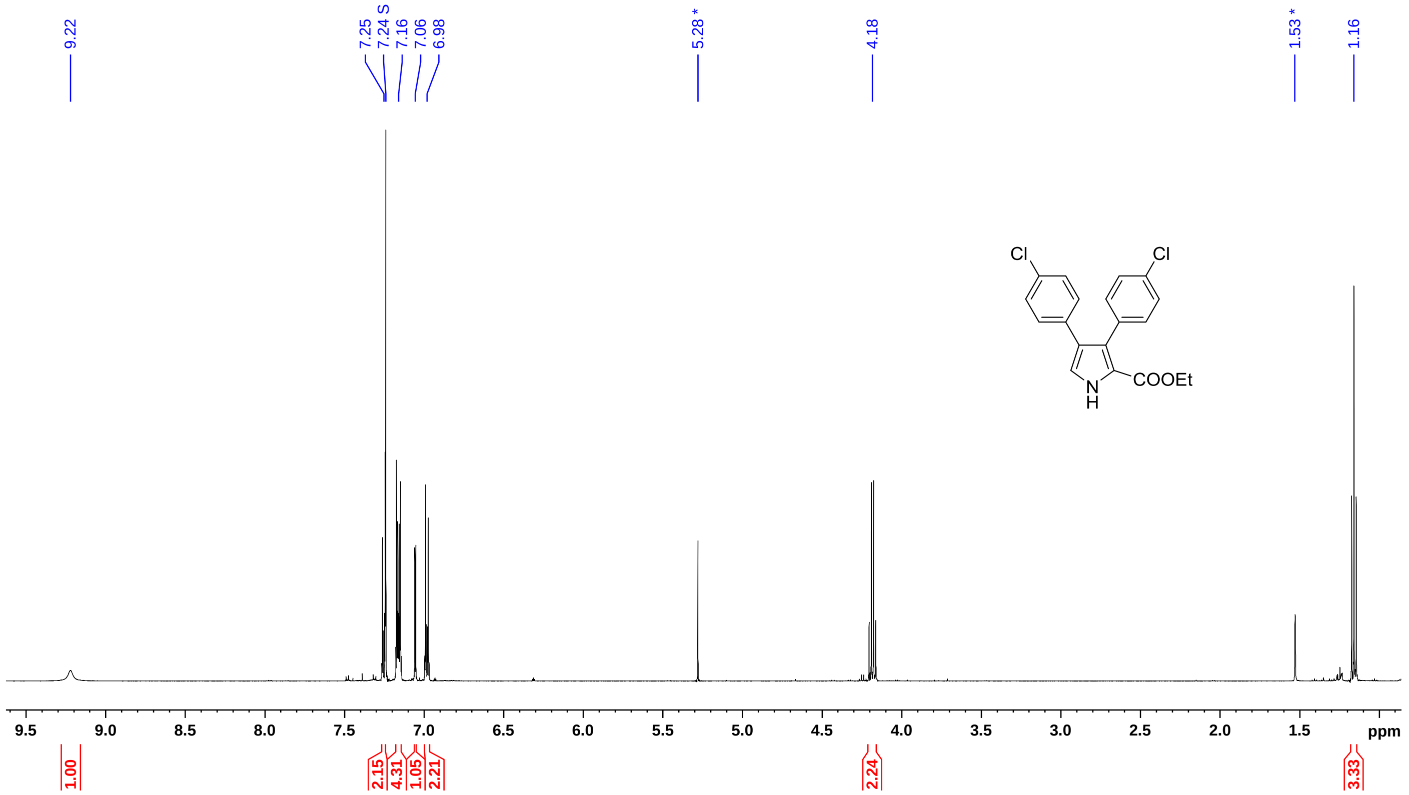

Figure S30. ^{1}H NMR spectrum of **S1** (chloroform-*d*, 500 MHz, 300 K).

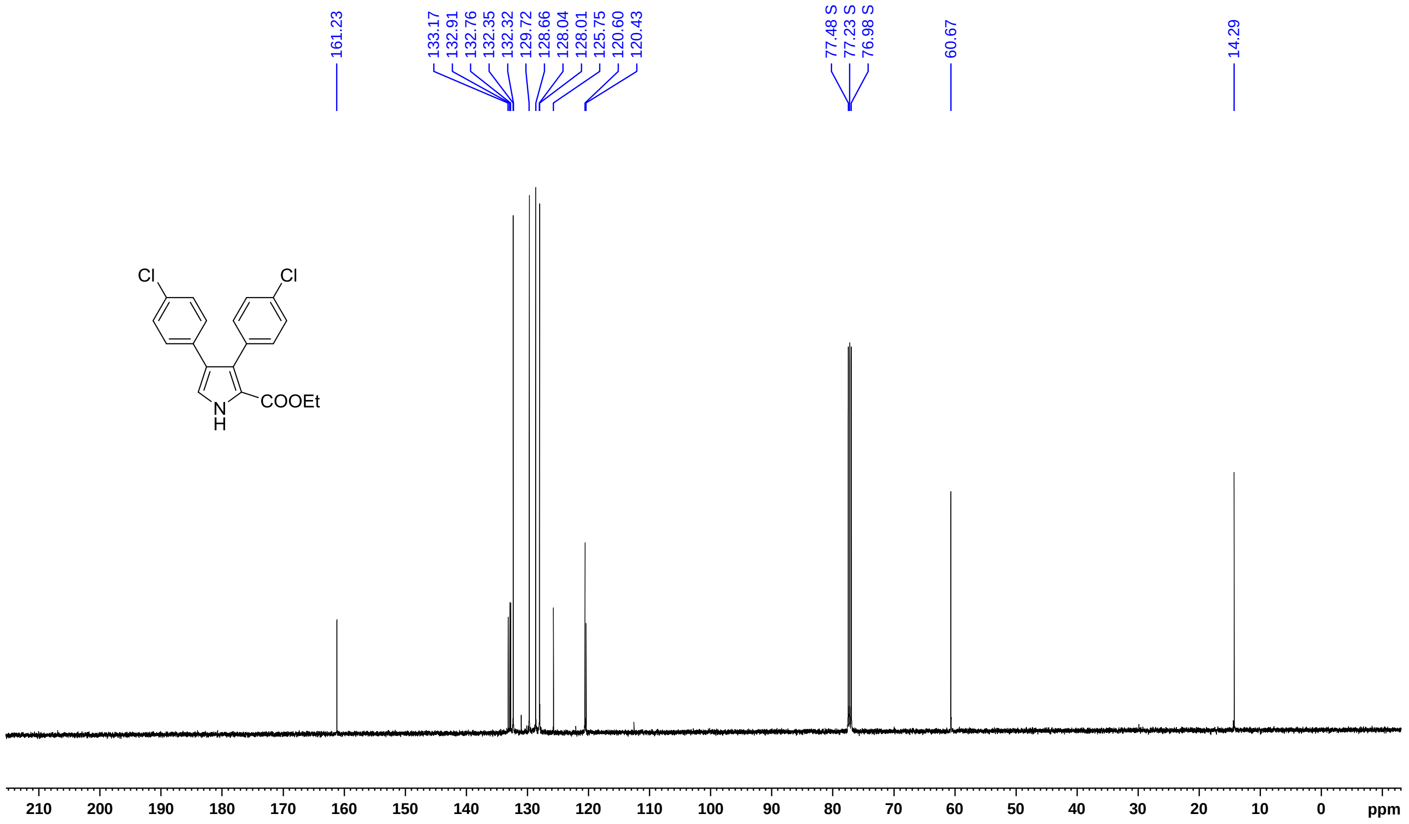

Figure S31. ^{13}C NMR spectrum of **S1** (chloroform-*d*, 125 MHz, 300 K).

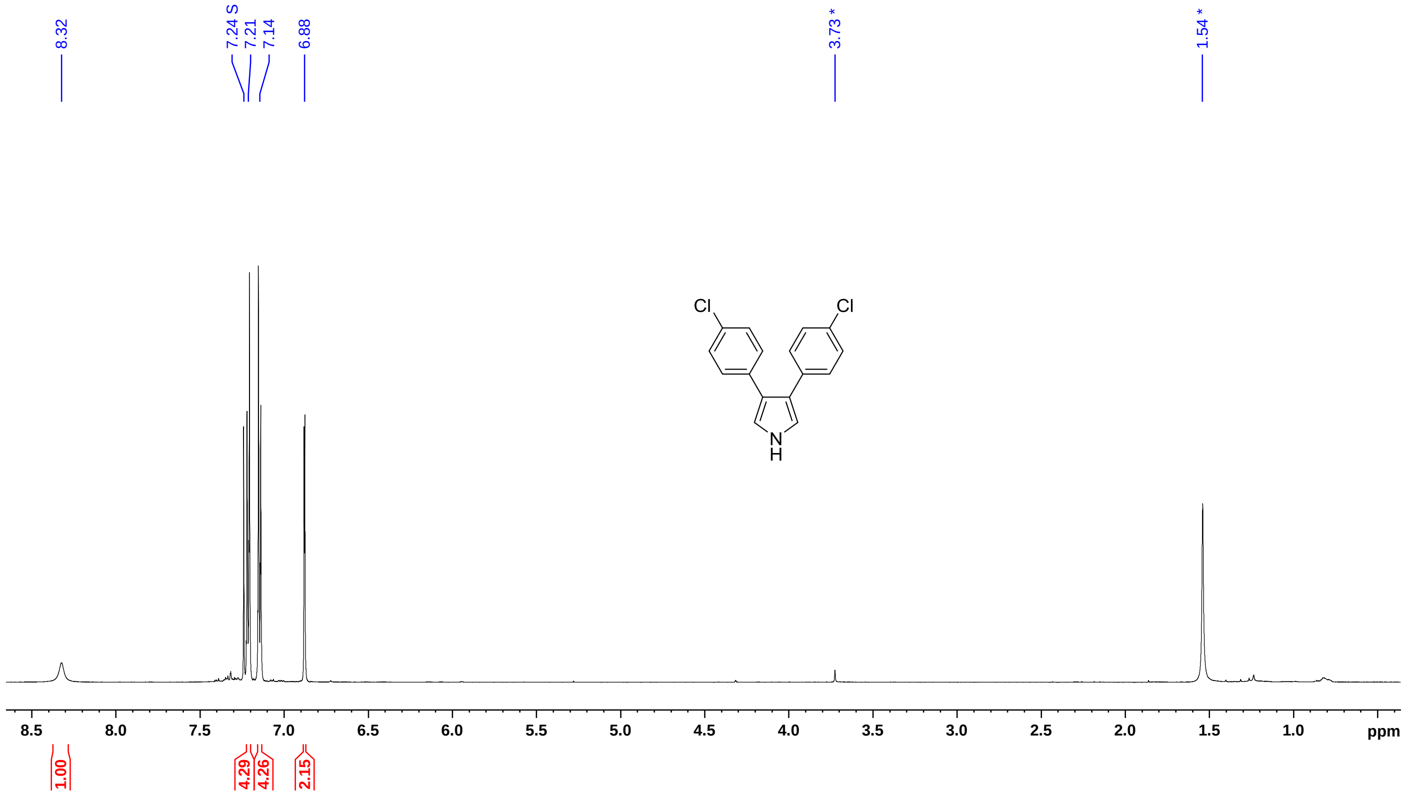

Figure S32. ^{1}H NMR spectrum of **S2** (chloroform-*d*, 600 MHz, 300 K).

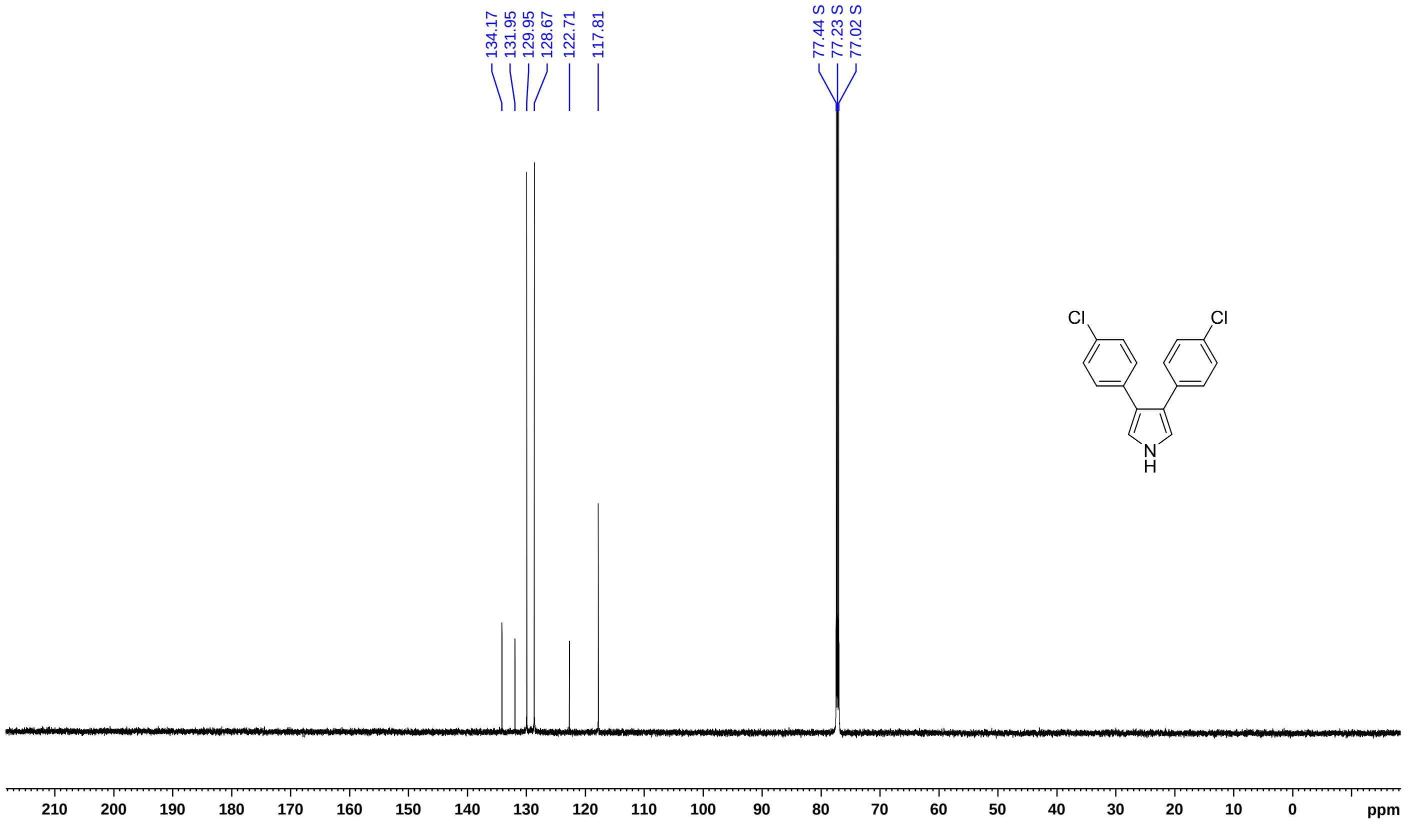

Figure S33. ^{13}C NMR spectrum of **S2** (chloroform-*d*, 151 MHz, 300 K).

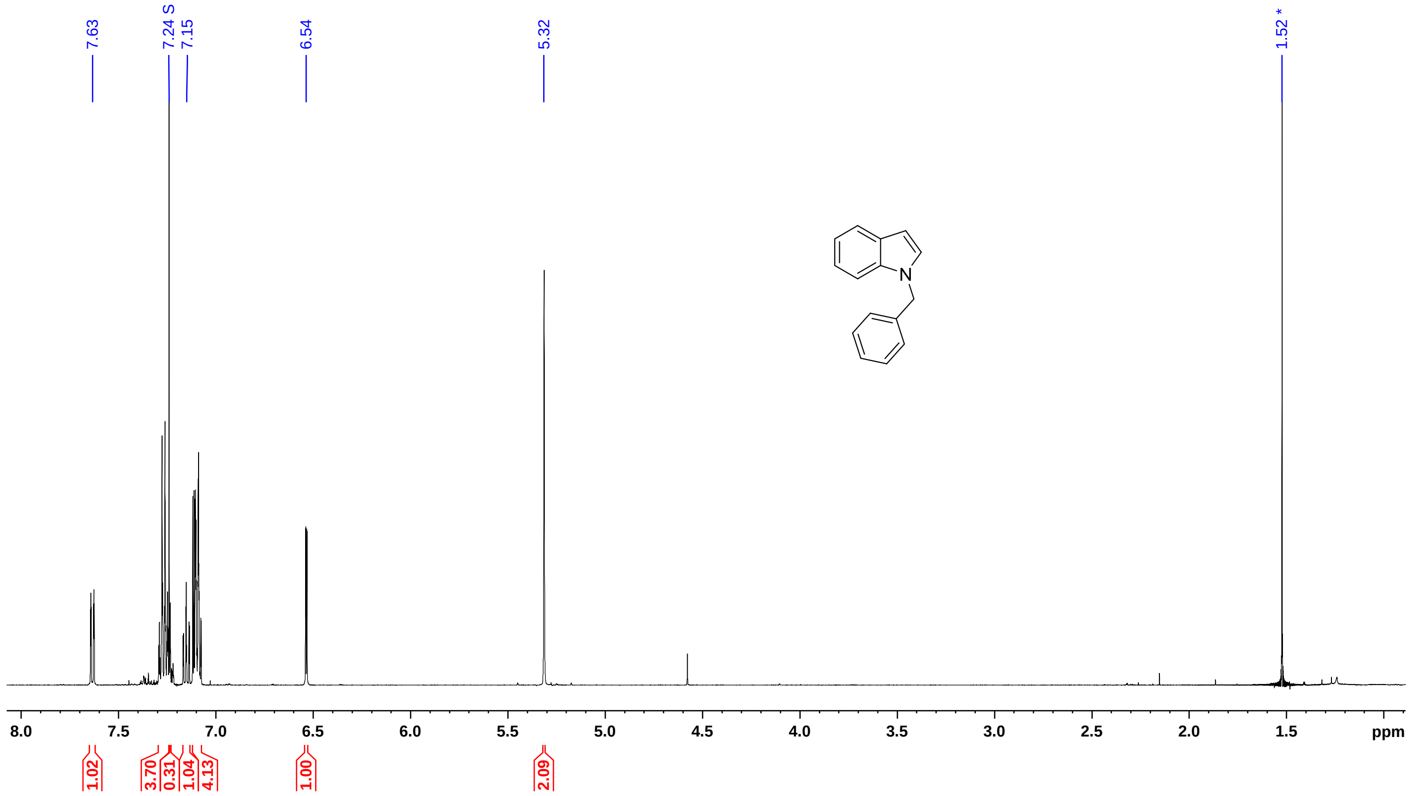

Figure S34. ^{1}H NMR spectrum of **6b** (chloroform-*d*, 500 MHz, 298 K).

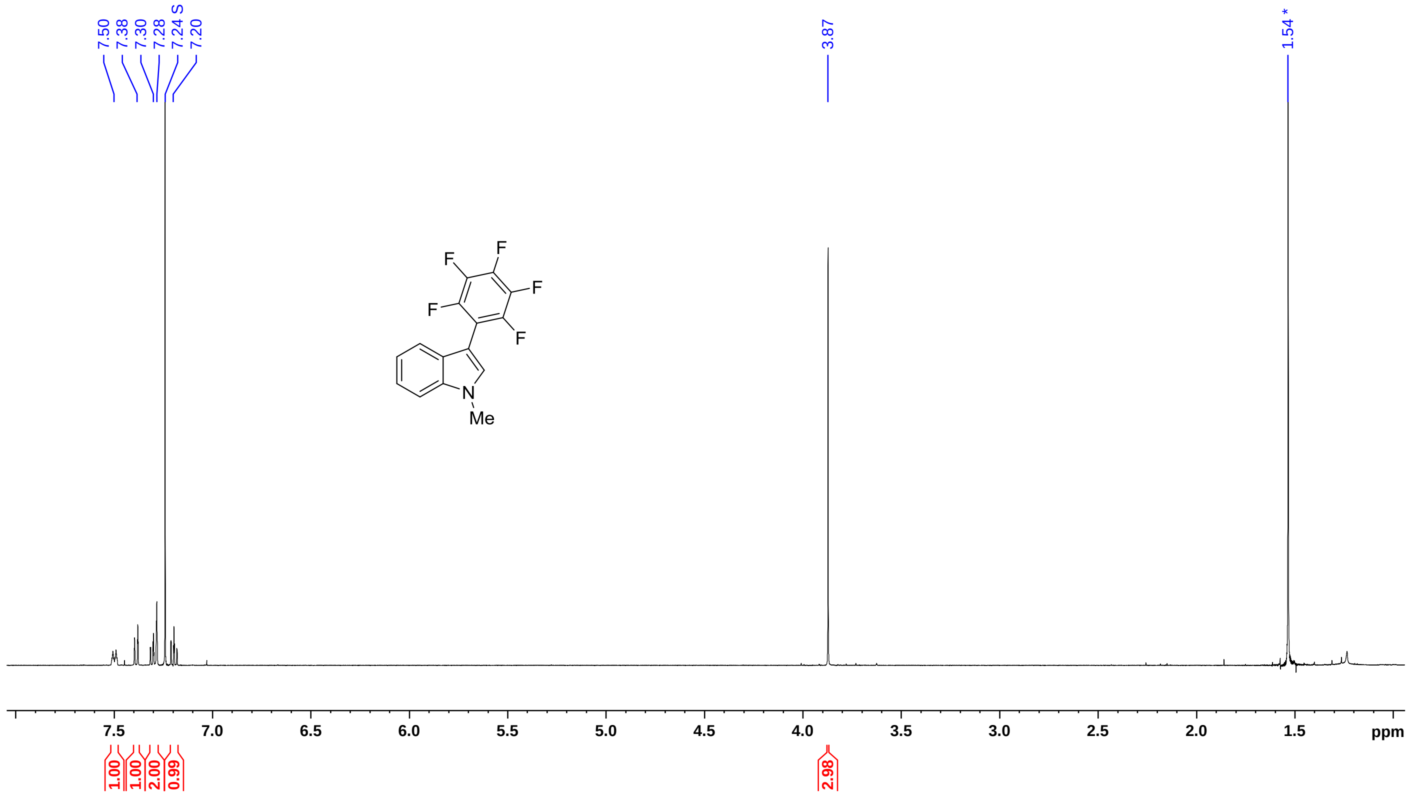

Figure S35. ^{1}H NMR spectrum of **7a** (chloroform-*d*, 500 MHz, 298 K).

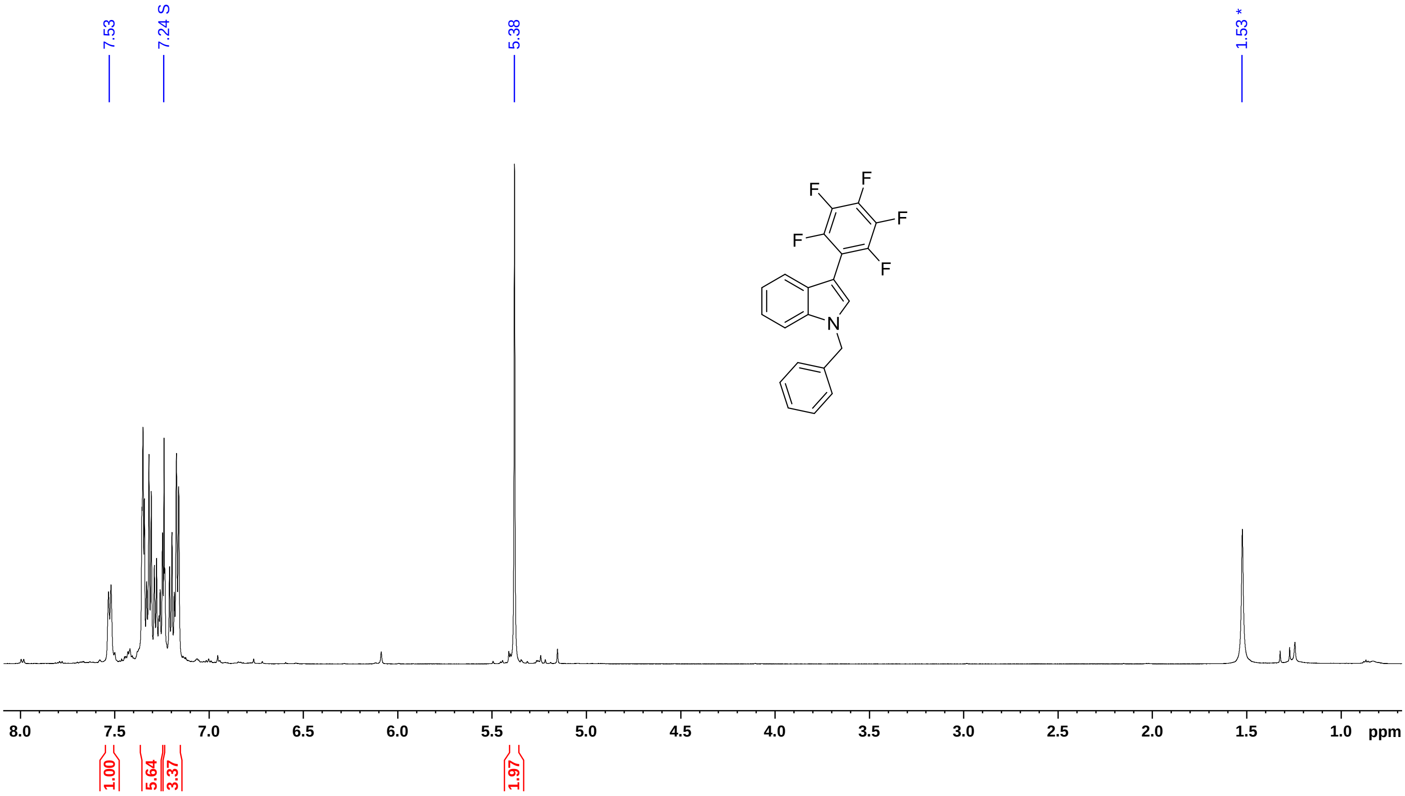

Figure S36. ^{1}H NMR spectrum of **7b** (chloroform-*d*, 600 MHz, 300 K).

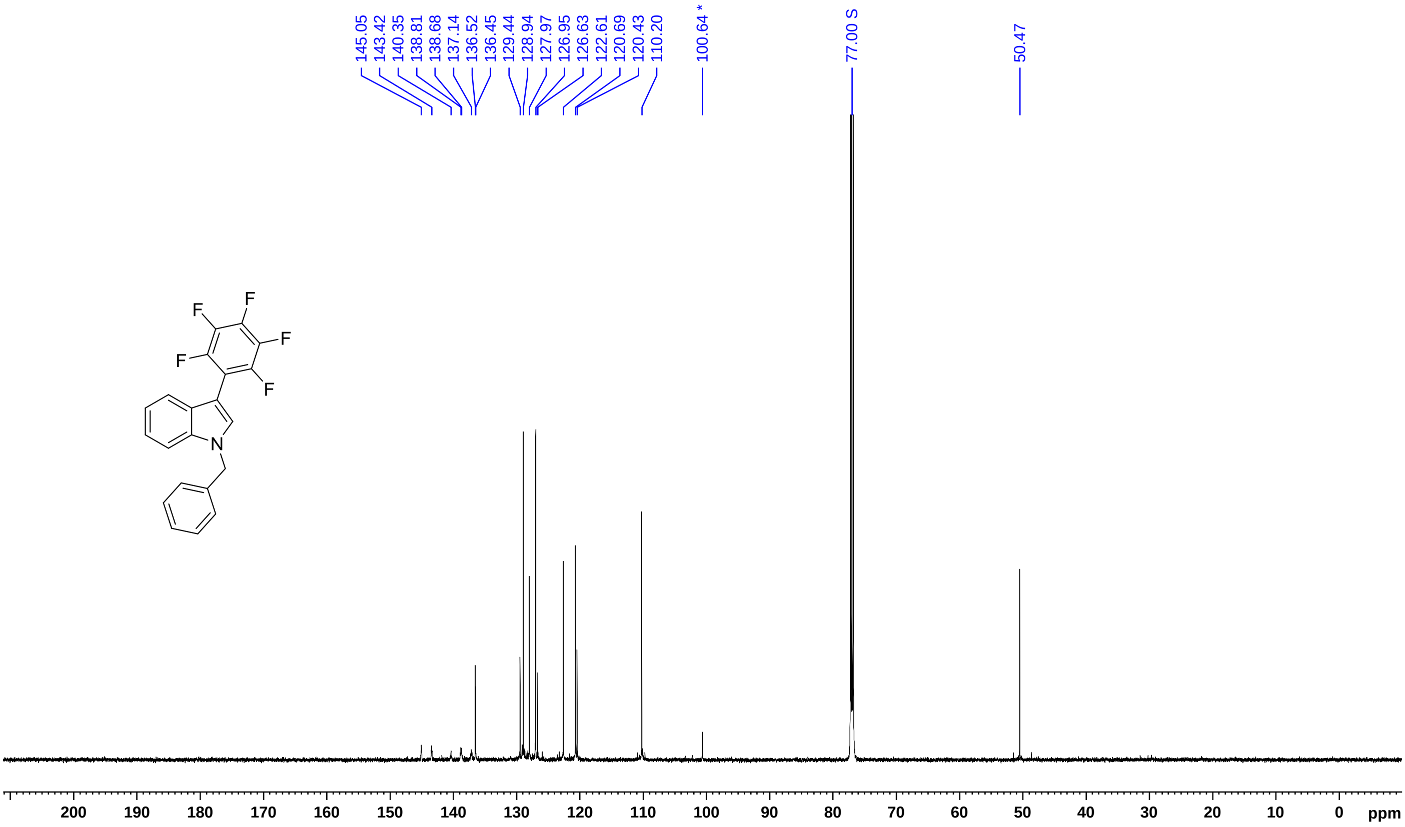

Figure S37. ^{13}C NMR spectrum of **7b** (chloroform-*d*, 151 MHz, 300 K).

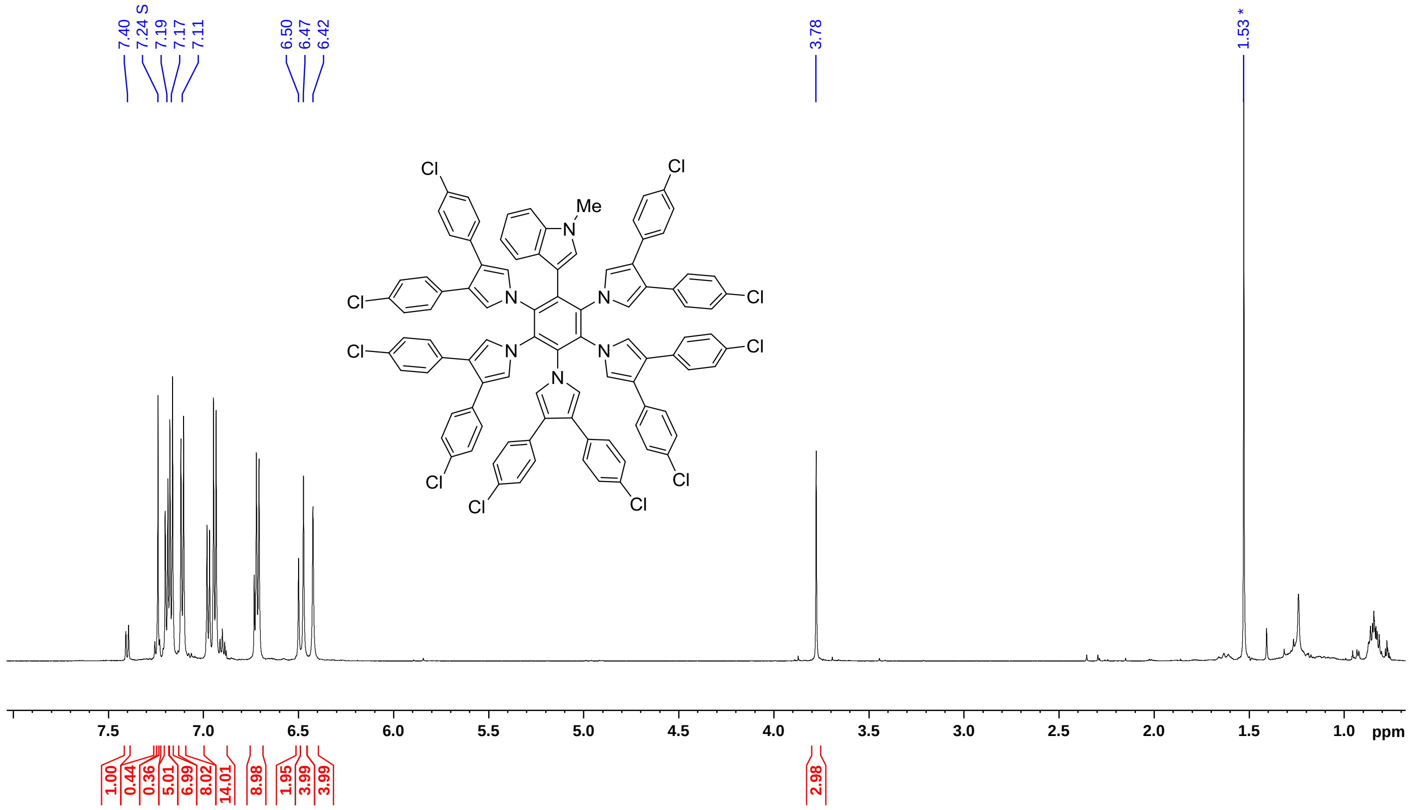

Figure S38. ^{1}H NMR spectrum of **8a** (chloroform-*d*, 600 MHz, 300 K).

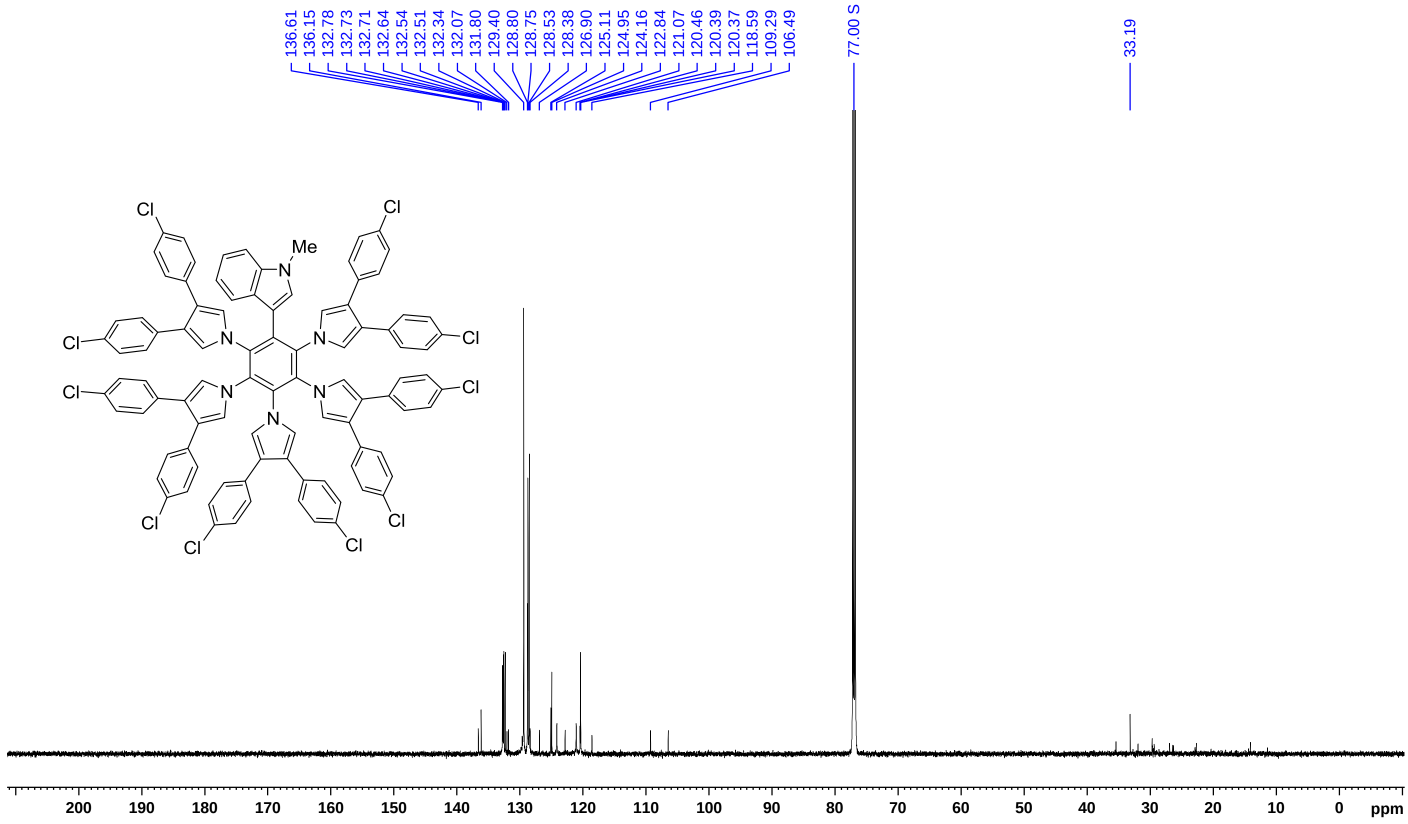

Figure S39. ^{13}C NMR spectrum of **8a** (chloroform-*d*, 151 MHz, 300 K).

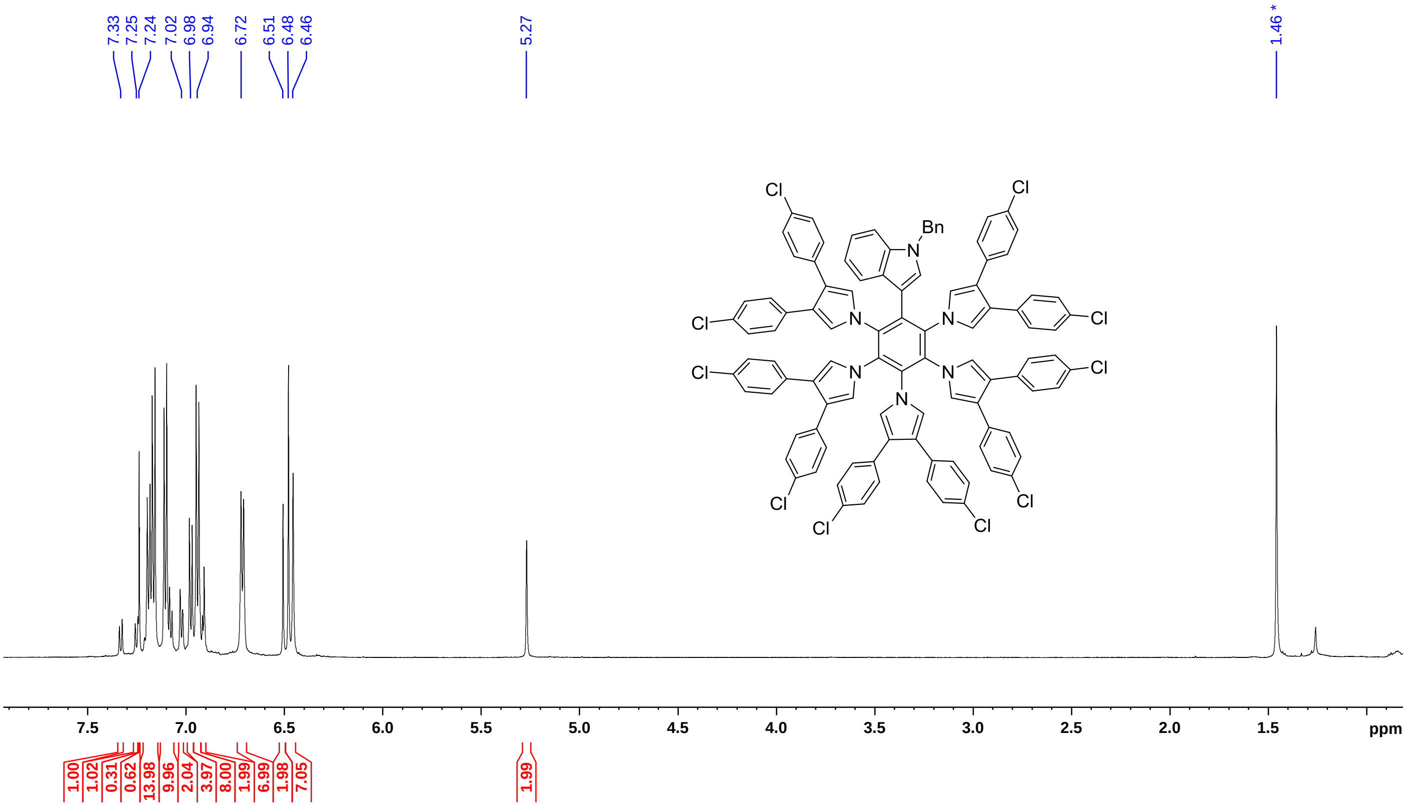

Figure S40. ^{1}H NMR spectrum of **8b** (chloroform-*d*, 600 MHz, 300 K).

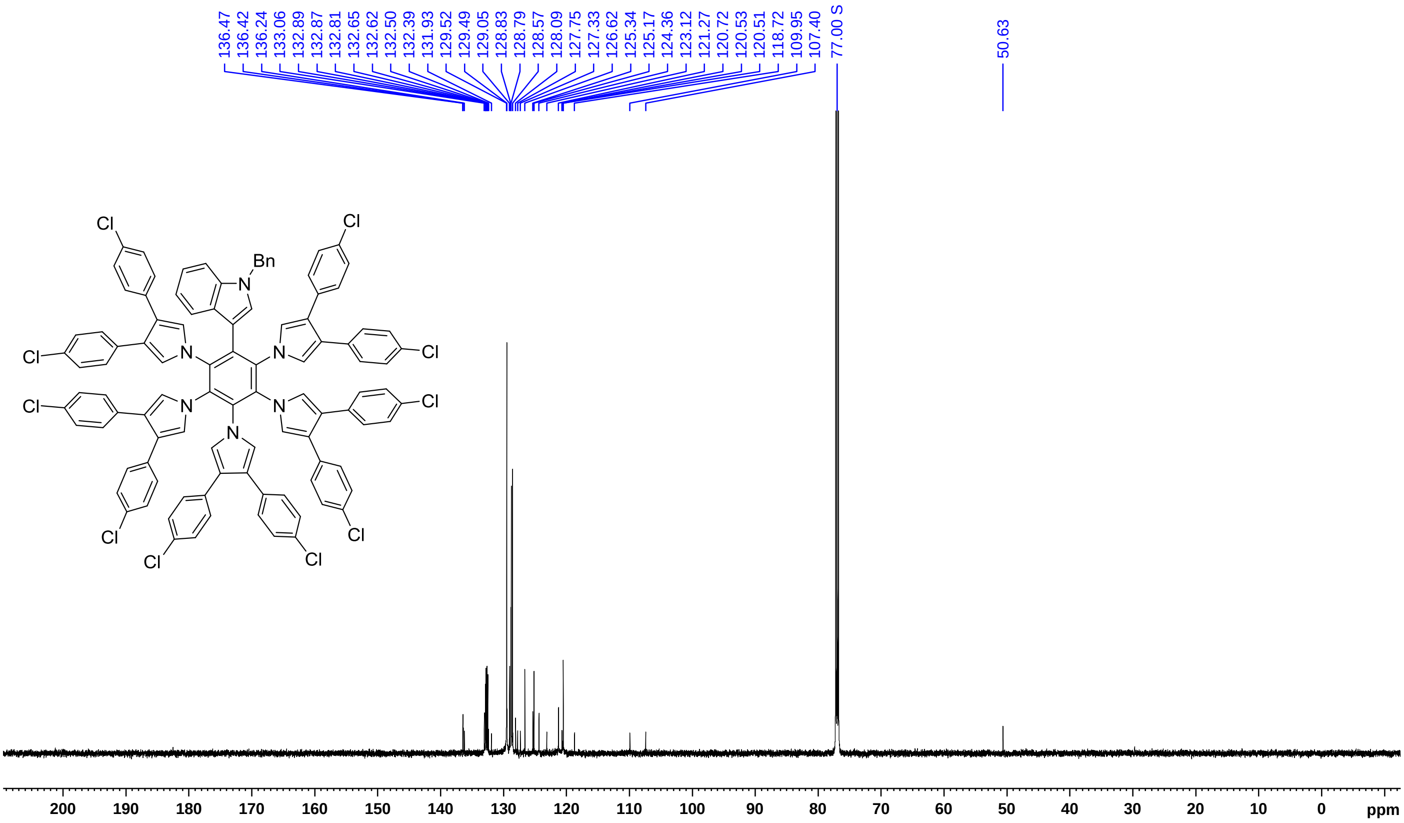

Figure S41. ^{13}C NMR spectrum of **8b** (chloroform-*d*, 151 MHz, 300 K).

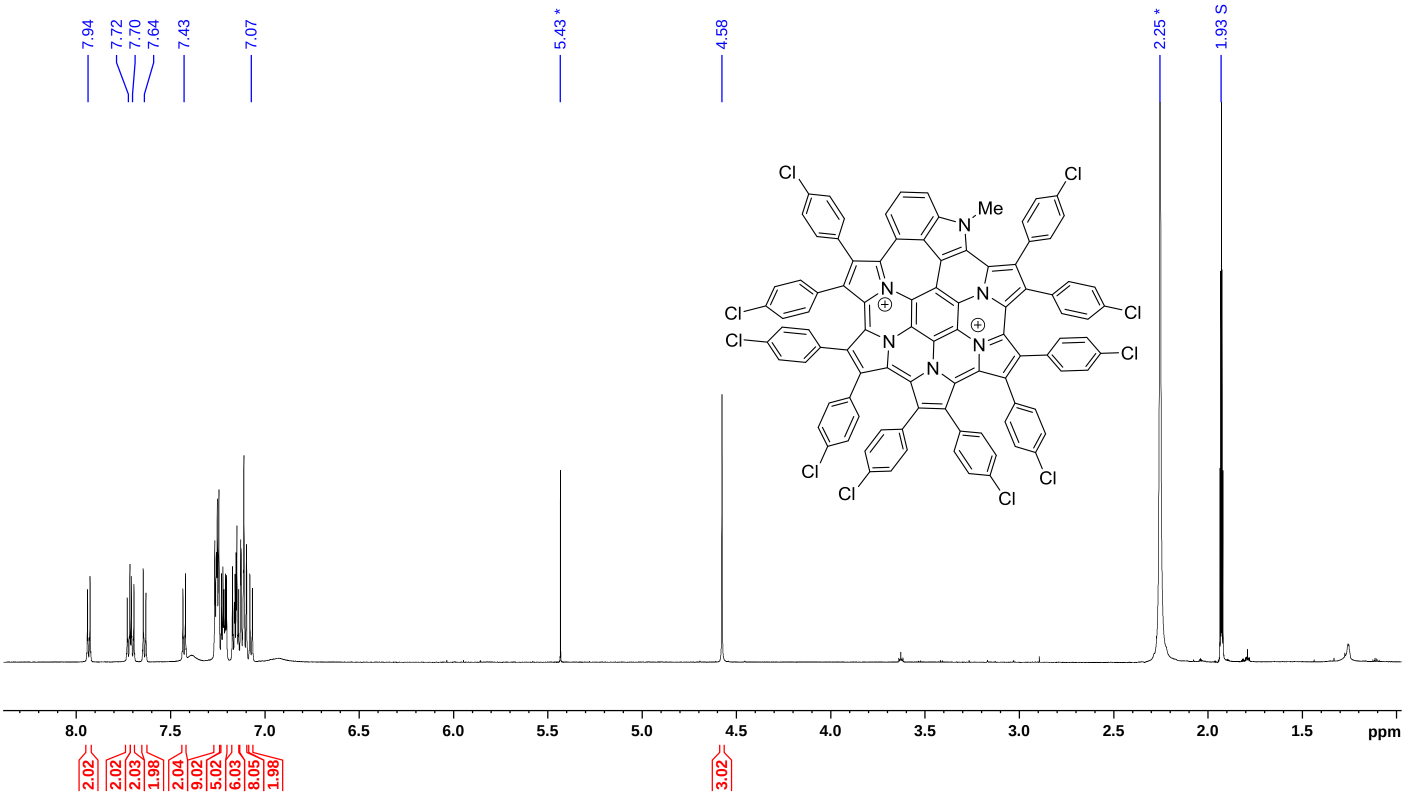

Figure S42. ^{1}H NMR spectrum of **5a**$^{2+}$ (acetonitrile-d_3, 600 MHz, 300 K).

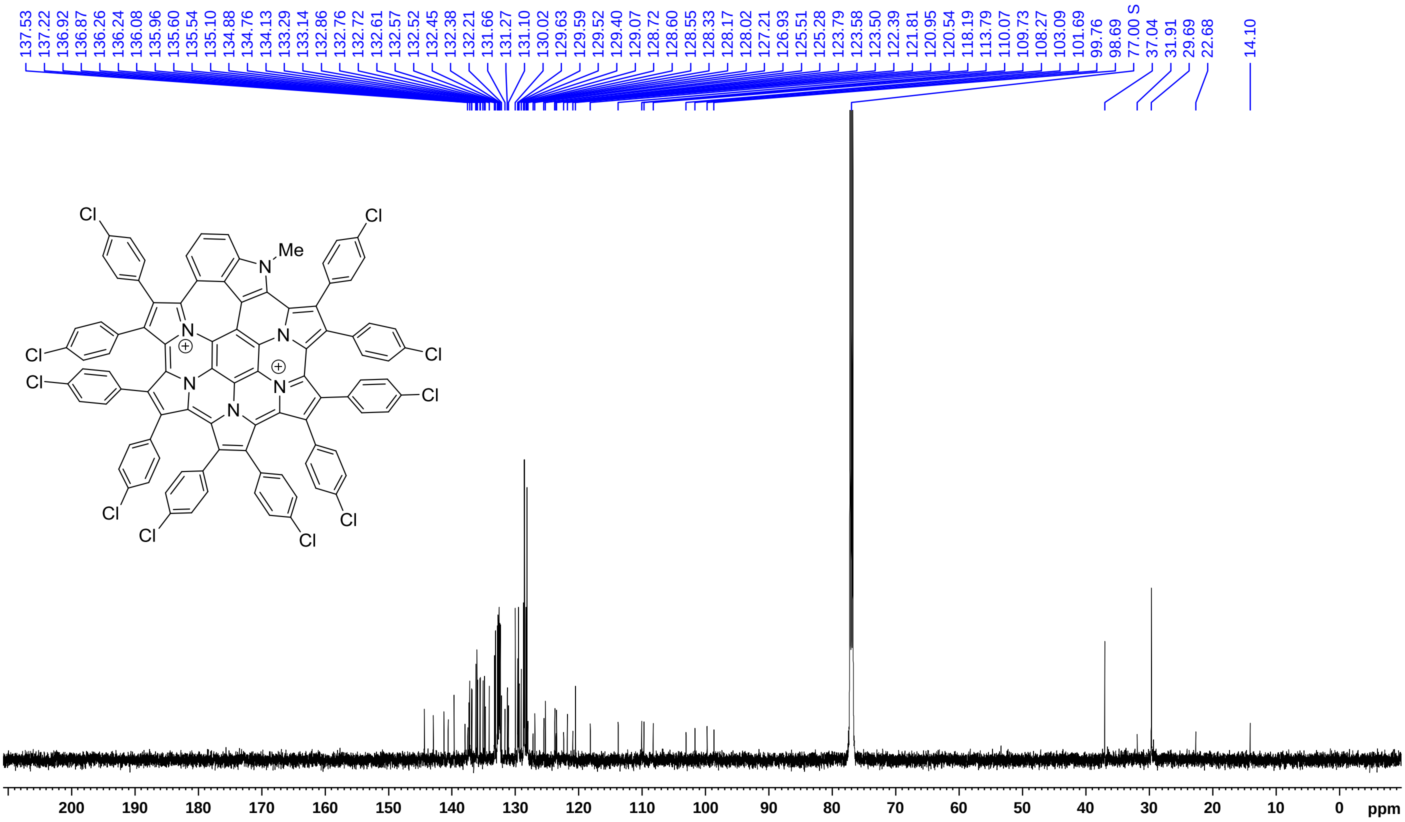

Figure S43. ^{13}C NMR spectrum of **5a**$^{2+}$ (chloroform-d, 151 MHz, 300 K).

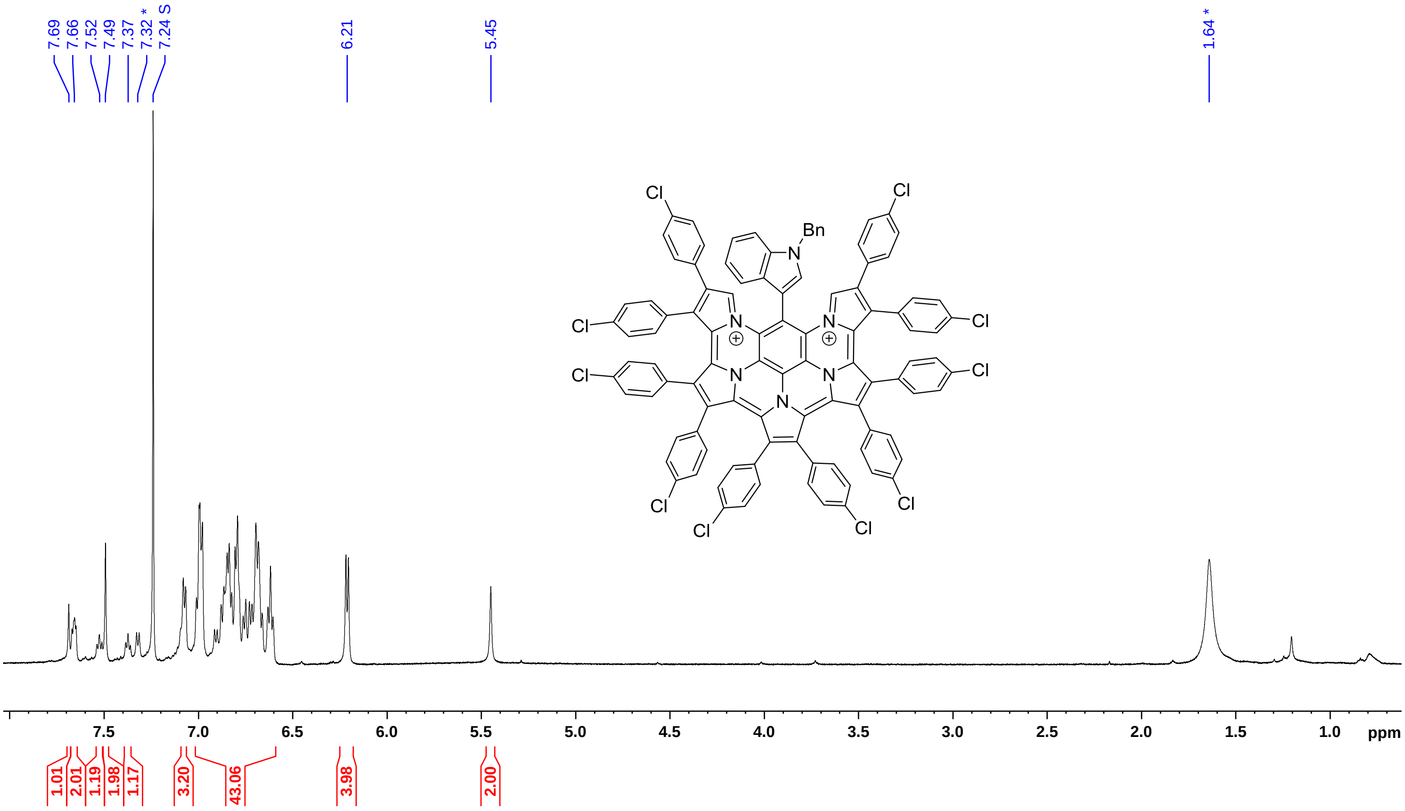

Figure S44. ^{1}H NMR spectrum of **9b^{2+}** (chloroform-*d*, 600 MHz, 260 K).

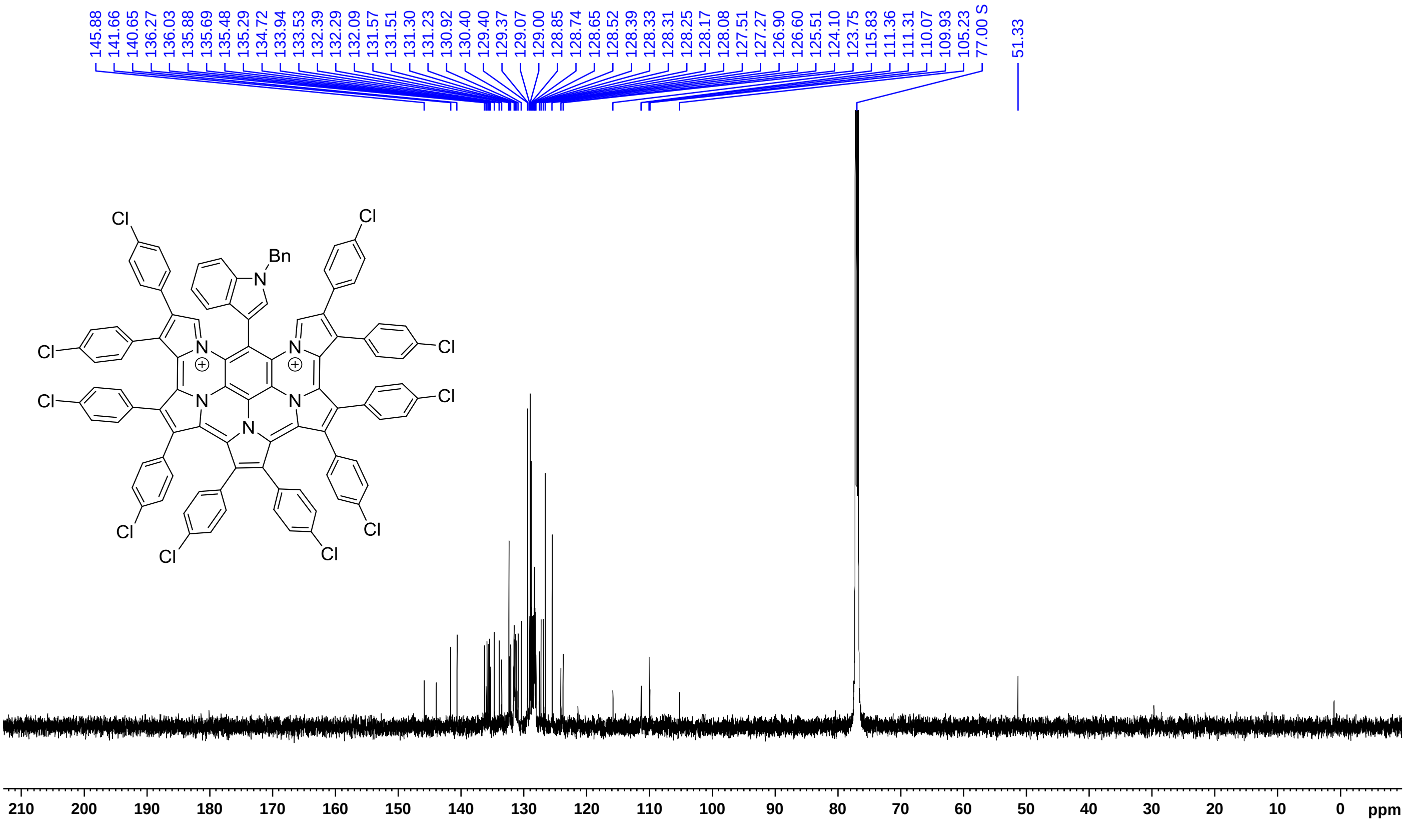

Figure S45. ^{13}C NMR spectrum of **9b^{2+}** (chloroform-*d*, 151 MHz, 260 K).

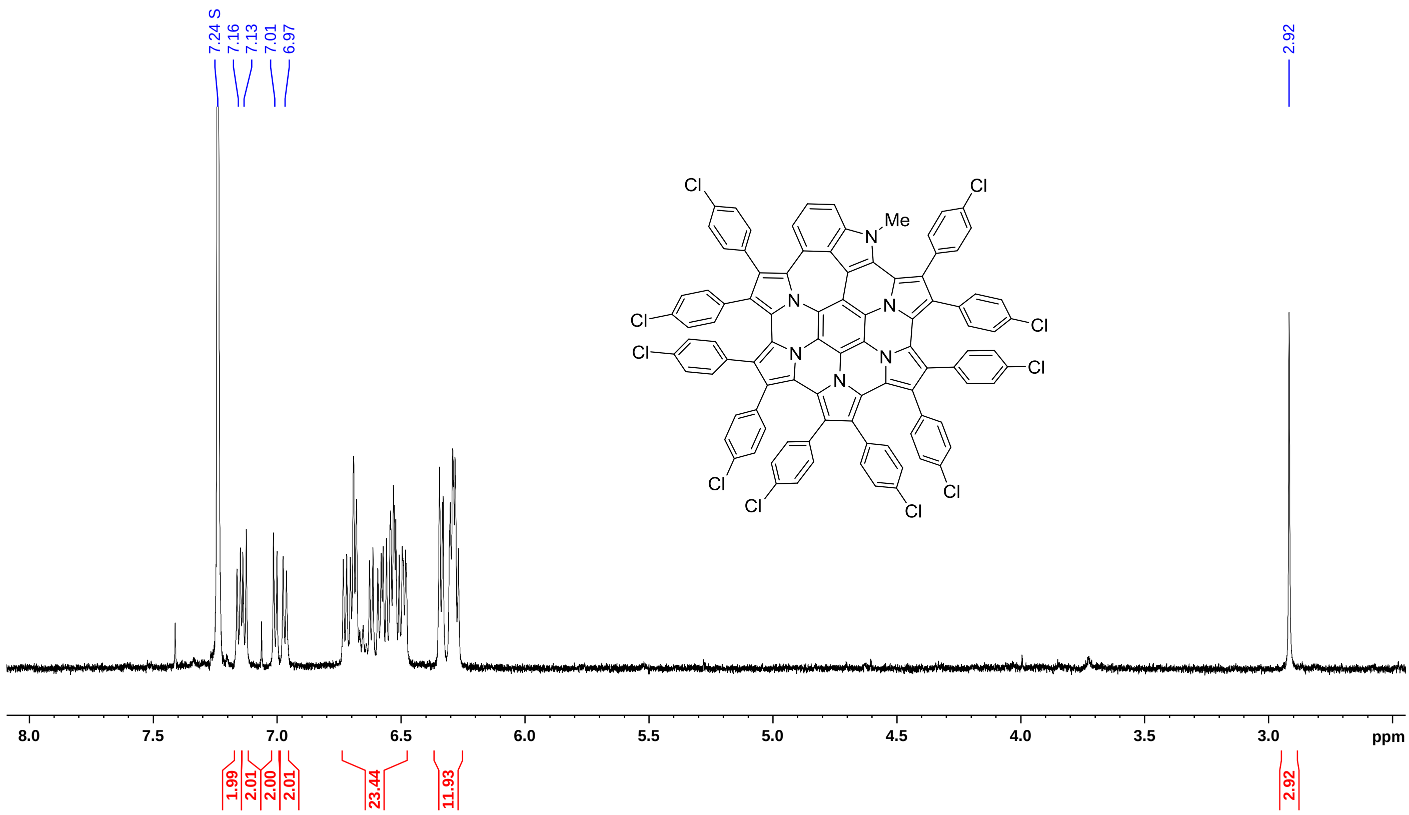

Figure S46. ^{1}H NMR spectrum of **5a** (chloroform-*d*, 600 MHz, 300 K).

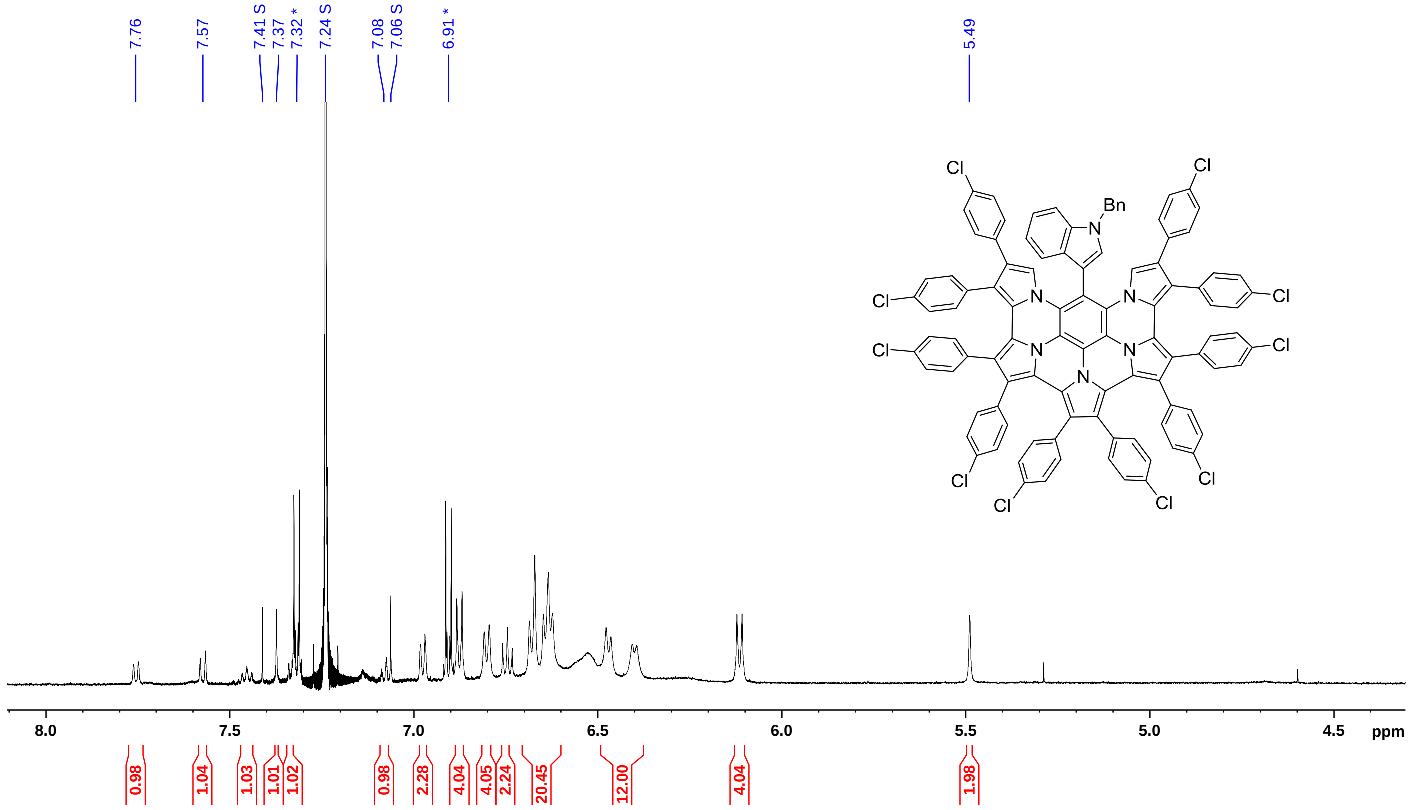

Figure S47. ^{1}H NMR spectrum of **9b** (chloroform-*d*, 600 MHz, 260 K).

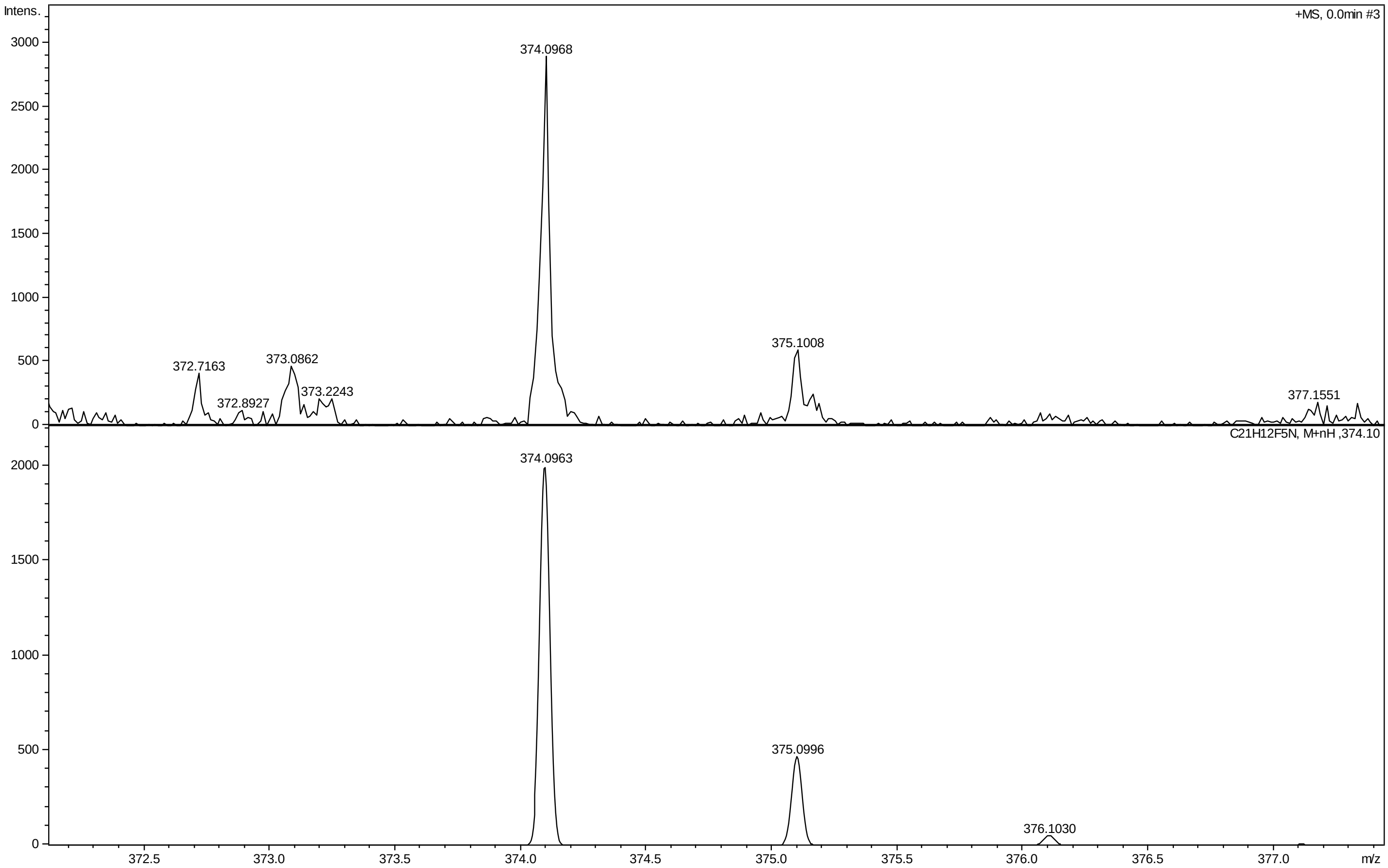

Figure S48. High resolution mass spectrum of **6b** (ESI+, top: experimental, bottom: simulated).

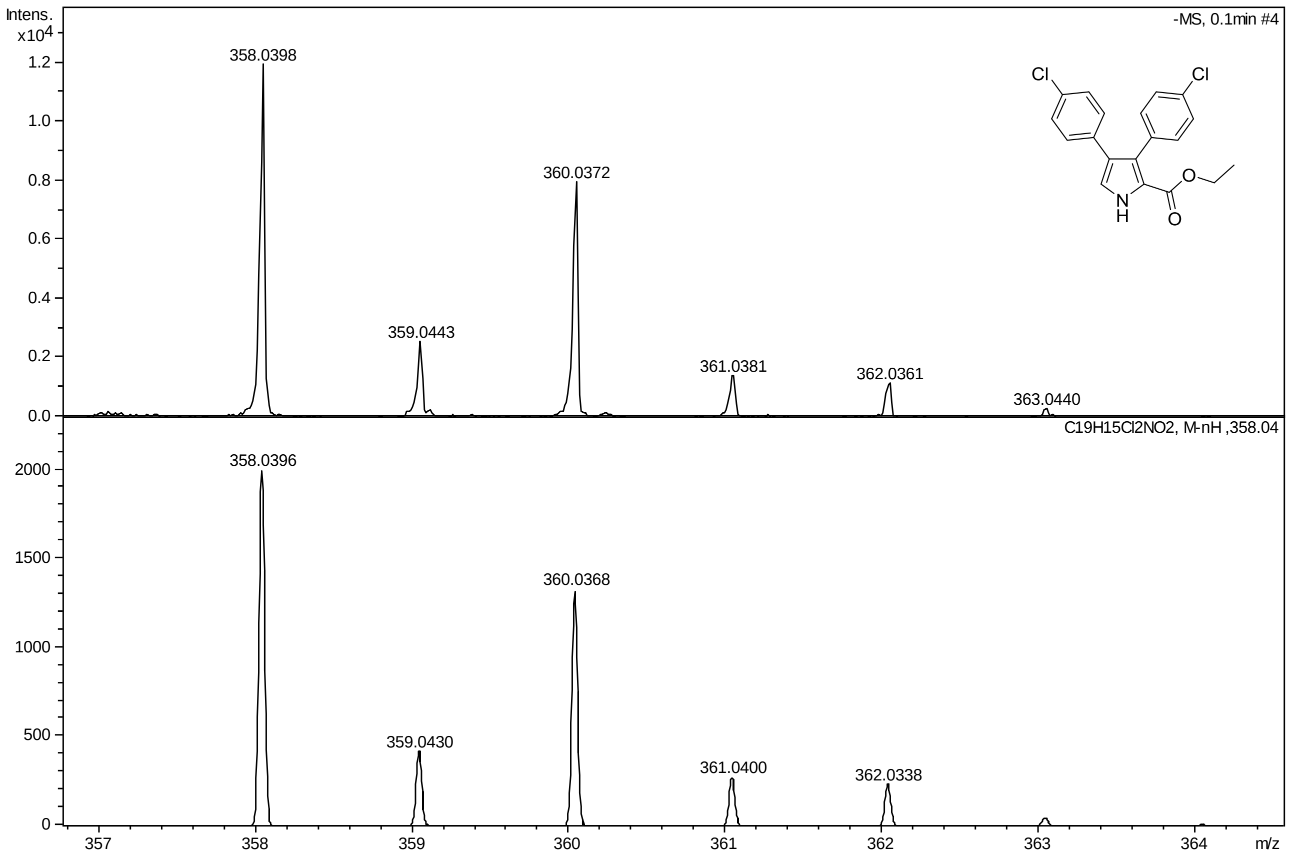

Figure S49. High resolution mass spectrum of **S2** (ESI+, top: experimental, bottom: simulated).

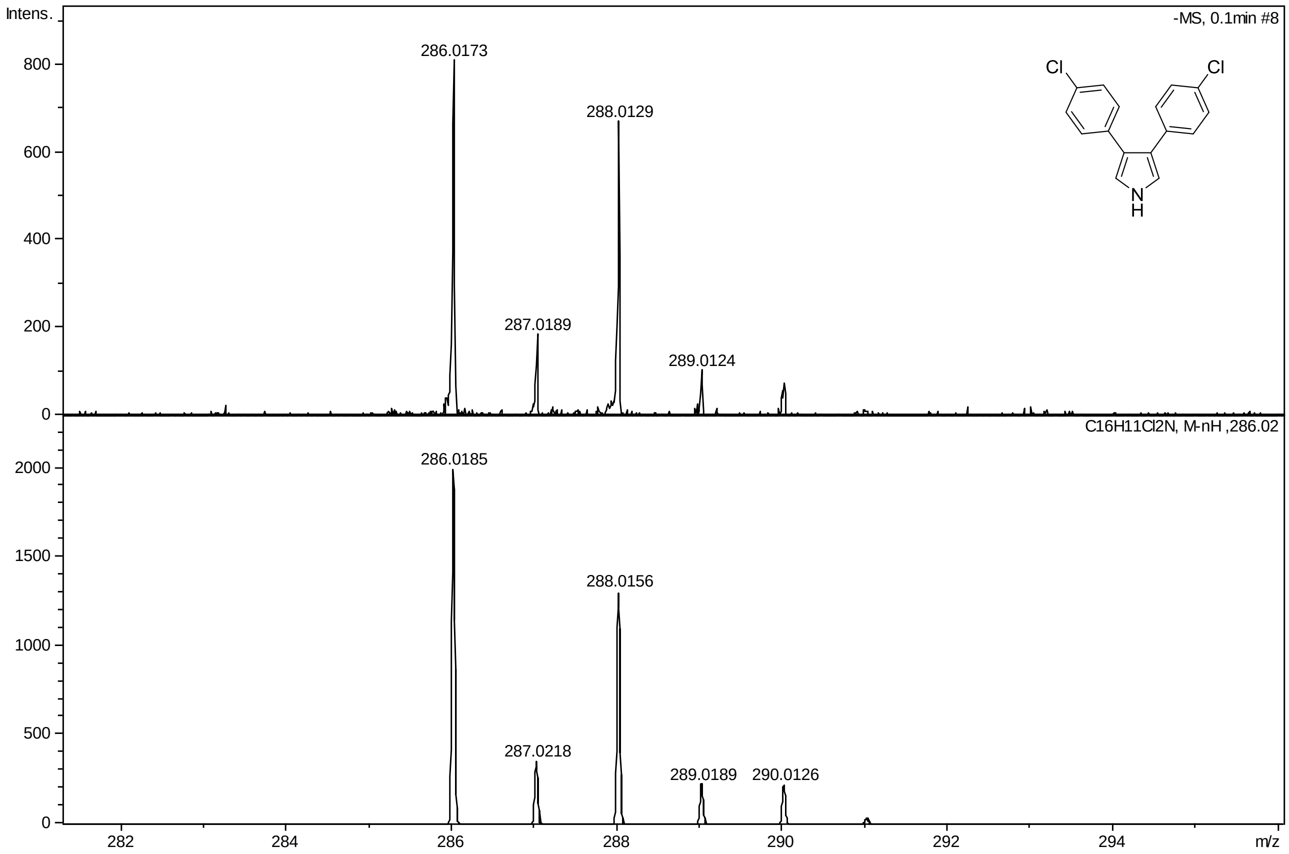

Figure S50. High resolution mass spectrum of **S3** (ESI+, top: experimental, bottom: simulated).

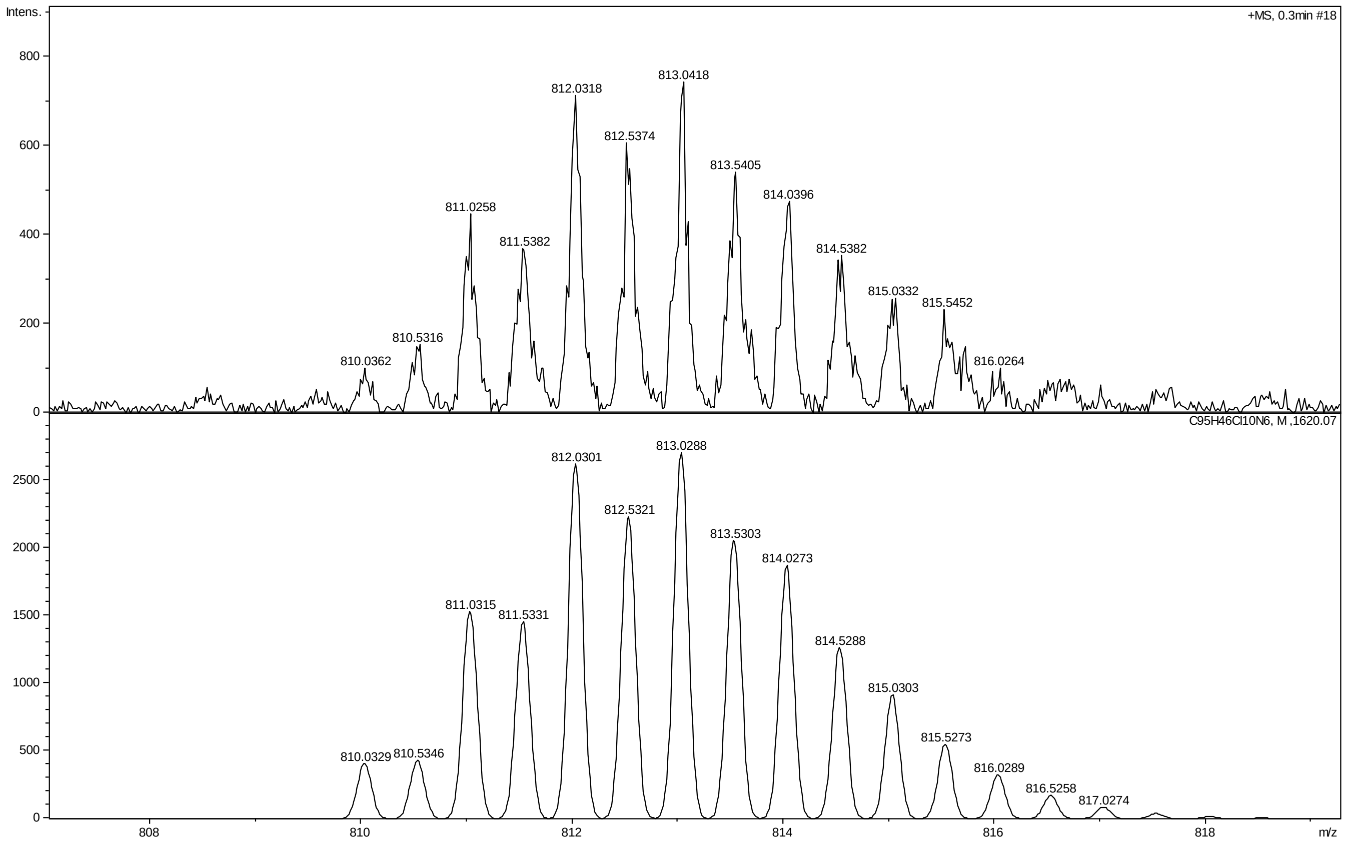

Figure S51. High resolution mass spectrum of **5a**$^{2+}$ (ESI+, top: experimental, bottom: simulated).

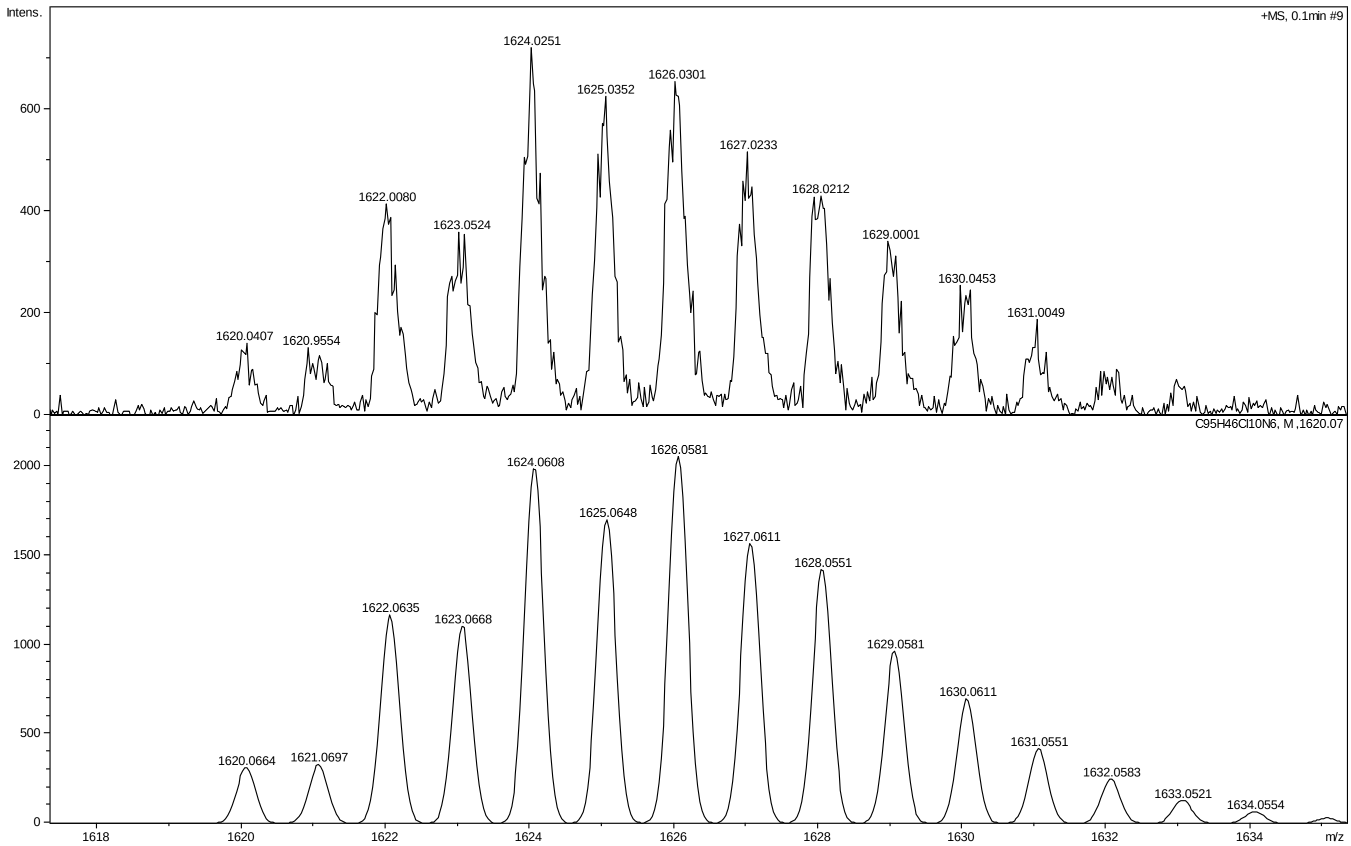

Figure S52. High resolution mass spectrum of **5a**$^{+}$ (ESI+, top: experimental, bottom: simulated).

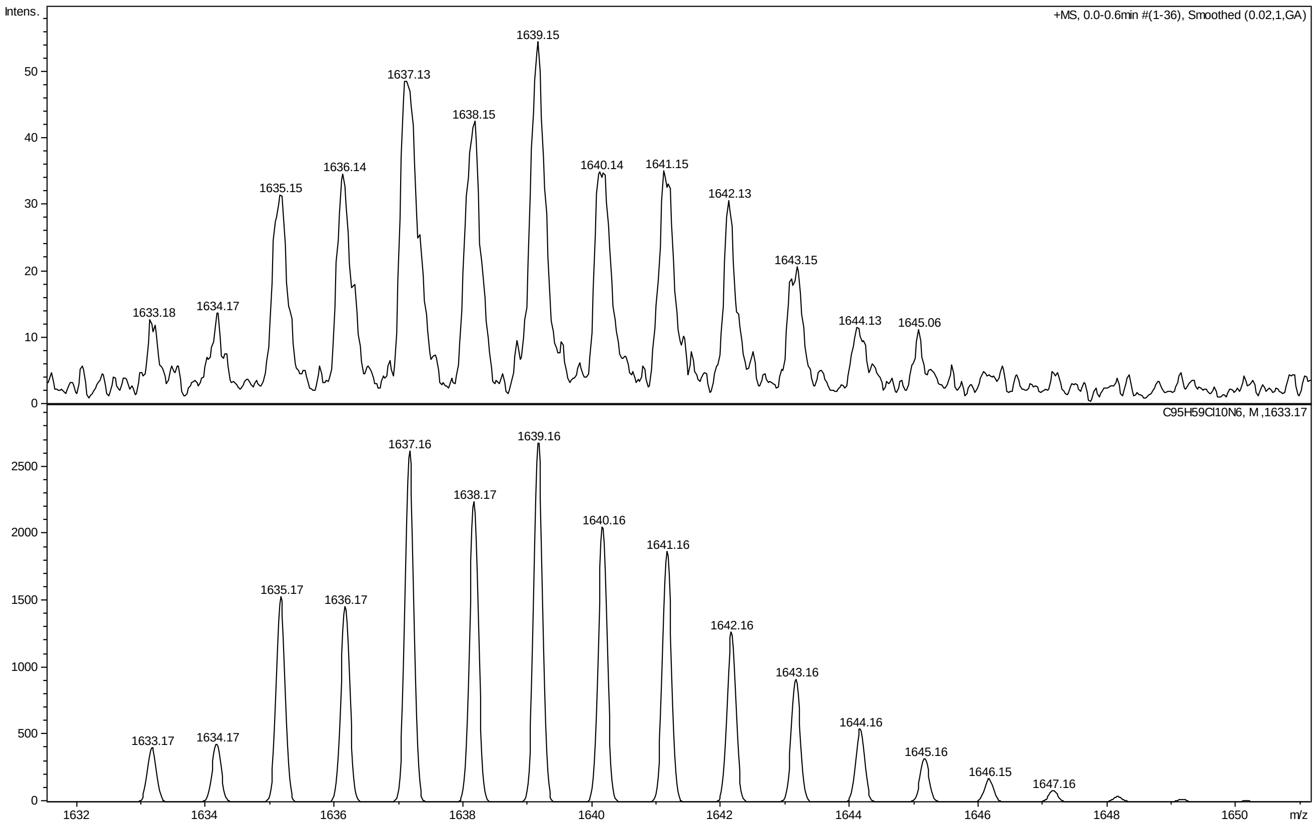

Figure S53. High resolution mass spectrum of **8a** (ESI+, top: experimental, bottom: simulated).

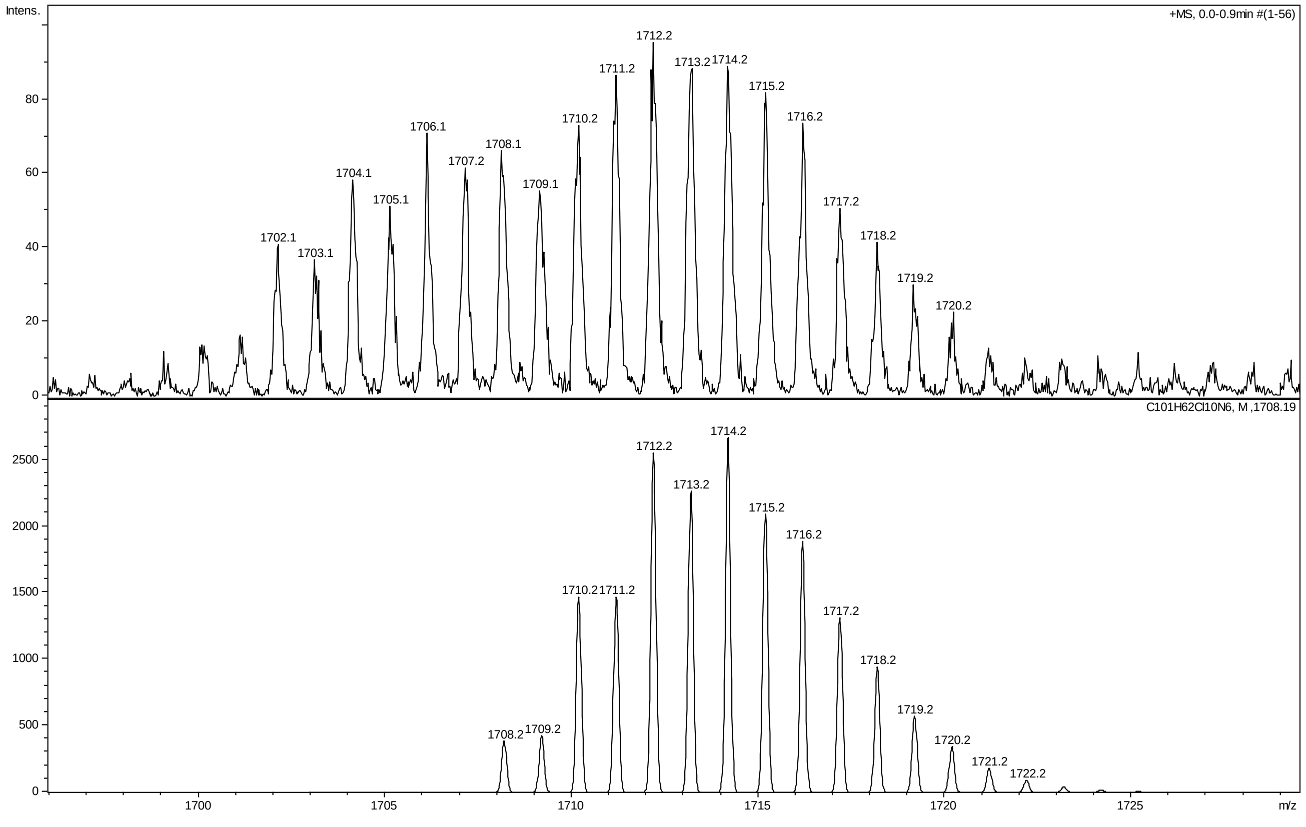

Figure S54. High resolution mass spectrum of **8b** (ESI+, top: experimental, bottom: simulated). Partial dehydrogenation in the source is observed.

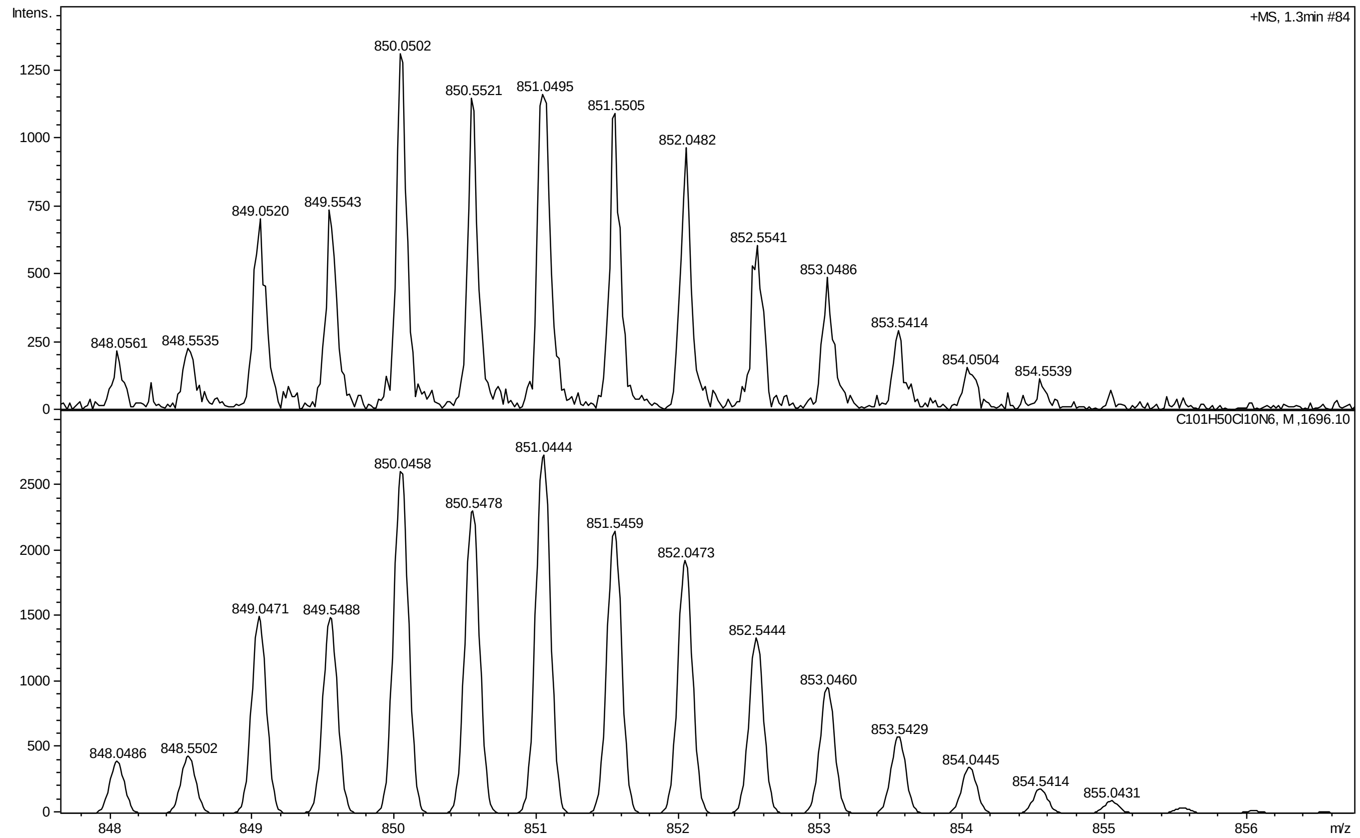

Figure S55. High resolution mass spectrum of **5b**$^{2+}$ (ESI+, top: experimental, bottom: simulated).

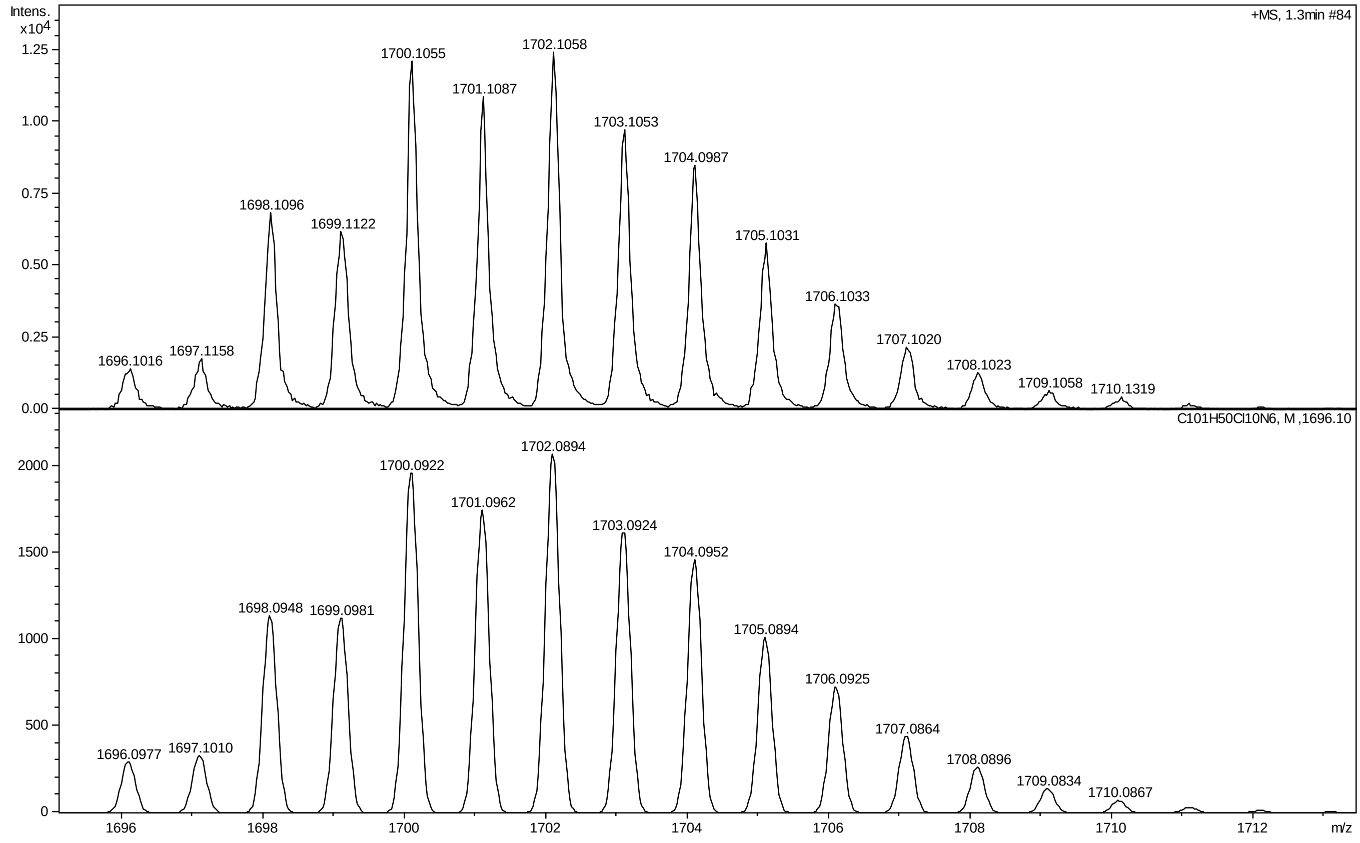

Figure S56. High resolution mass spectrum of **5b**$^{+}$ (ESI+, top: experimental, bottom: simulated).

References

(1) Frisch, M. J.; Trucks, G. W.; Schlegel, H. B.; Scuseria, G. E.; Robb, M. A.; Cheeseman, J. R.; Scalmani, G.; Barone, V.; Mennucci, B.; Petersson, G. A.; Nakatsuji, H.; Caricato, M.; Li, X.; Hratchian, H. P.; Izmaylov, A. F.; Bloino, J.; Zheng, G.; Sonnenberg, J. L.; Hada, M.; Ehara, M.; Toyota, K.; Fukuda, R.; Hasegawa, J.; Ishida, M.; Nakajima, T.; Honda, Y.; Kitao, O.; Nakai, H.; Vreven, T.; Montgomery, Jr., J. A.; Peralta, J. E.; Ogliaro, F.; Bearpark, M.; Heyd, J. J.; Brothers, E.; Kudin, K. N.; Staroverov, V. N.; Kobayashi, R.; Normand, J.; Raghavachari, K.; Rendell, A.; Burant, J. C.; Iyengar, S. S.; Tomasi, J.; Cossi, M.; Rega, N.; Millam, J. M.; Klene, M.; Knox, J. E.; Cross, J. B.; Bakken, V.; Adamo, C.; Jaramillo, J.; Gomperts, R.; Stratmann, R. E.; Yazyev, O.; Austin, A. J.; Cammi, R.; Pomelli, C.; Ochterski, J. W.; Martin, R. L.; Morokuma, K.; Zakrzewski, V. G.; Voth, G. A.; Salvador, P.; Dannenberg, J. J.; Dapprich, S.; Daniels, A. D.; Farkas, Ö.; Foresman, J. B.; Ortiz, J. V.; Cioslowski, J.; Fox, D. J. *Gaussian 09 Revision D.01*.

(2) Becke, A. D. *Phys. Rev. A* **1988**, *38* (6), 3098–3100.

(3) Becke, A. D. *J. Chem. Phys.* **1993**, *98* (7), 5648–5652.

(4) Lee, C.; Yang, W.; Parr, R. G. *Phys. Rev. B* **1988**, *37* (2), 785–789.

(5) Tomasi, J.; Mennucci, B.; Cammi, R. *Chem. Rev.* **2005**, *105* (8), 2999–3094.

(6) Kulp, S. S.; Caldwell, C. B. *J. Org. Chem.* **1980**, *45* (1), 171–173.

(7) Xu, S.; Huang, X.; Hong, X.; Xu, B. *Org. Lett.* **2012**, *14* (17), 4614–4617.

(8) He, C.-Y.; Fan, S.; Zhang, X. *J. Am. Chem. Soc.* **2010**, *132* (37), 12850–12852.
